# Melatonin and health: an umbrella review of health outcomes and biological mechanisms of action

**DOI:** 10.1186/s12916-017-1000-8

**Published:** 2018-02-05

**Authors:** Pawel P. Posadzki, Ram Bajpai, Bhone Myint Kyaw, Nicola J. Roberts, Amnon Brzezinski, George I. Christopoulos, Ushashree Divakar, Shweta Bajpai, Michael Soljak, Gerard Dunleavy, Krister Jarbrink, Ei Ei Khaing Nang, Chee Kiong Soh, Josip Car

**Affiliations:** 1 Centre for Population Health Sciences, 11 Mandalay Road, Level 18 Clinical Sciences Building, Lee Kong Chian School of Medicine, Novena Campus, Nanyang Technological University , Singapore, 308232 Singapore; 2 School of Health and Life Sciences, Glasgow Caledonian University, Glasgow, G4 0BA UK; 3 The Hebrew University Medical School, Hadassah Hebrew University Medical Center, 91120 Jerusalem, Israel; 4 Nanyang Business School, Division of Strategy Management and Organisation, Nanyang Technological University, Singapore, 639798 Singapore; 5 School of Civil and Environmental Engineering, College of Engineering, Nanyang Technological University, Singapore, 639798 Singapore; 6 Global eHealth Unit, School of Public Health, Imperial College London, London, W6 8RP UK

**Keywords:** Melatonin, Health, Effectiveness, Umbrella review, Systematic reviews, Meta-analyses

## Abstract

**Background:**

Our aims were to evaluate critically the evidence from systematic reviews as well as narrative reviews of the effects of melatonin (MLT) on health and to identify the potential mechanisms of action involved.

**Methods:**

An umbrella review of the evidence across systematic reviews and narrative reviews of endogenous and exogenous (supplementation) MLT was undertaken. The Oxman checklist for assessing the methodological quality of the included systematic reviews was utilised. The following databases were searched: MEDLINE, EMBASE, Web of Science, CENTRAL, PsycINFO and CINAHL. In addition, reference lists were screened. We included reviews of the effects of MLT on any type of health-related outcome measure.

**Results:**

Altogether, 195 reviews met the inclusion criteria. Most were of low methodological quality (mean -4.5, standard deviation 6.7). Of those, 164 did not pool the data and were synthesised narratively (qualitatively) whereas the remaining 31 used meta-analytic techniques and were synthesised quantitatively. Seven meta-analyses were significant with *P* values less than 0.001 under the random-effects model. These pertained to sleep latency, pre-operative anxiety, prevention of agitation and risk of breast cancer.

**Conclusions:**

There is an abundance of reviews evaluating the effects of exogenous and endogenous MLT on health. In general, MLT has been shown to be associated with a wide variety of health outcomes in clinically and methodologically heterogeneous populations. Many reviews stressed the need for more high-quality randomised clinical trials to reduce the existing uncertainties.

**Electronic supplementary material:**

The online version of this article (10.1186/s12916-017-1000-8) contains supplementary material, which is available to authorized users.

## Background

Circadian rhythms are biological processes that display endogenous, entrainable oscillation cycles that last approximately 24 hours (owing to the Earth’s rotation around its own axis) [1]. These rhythms tune internal physiology, behaviour and metabolism to external conditions and are considered to be a feature of most living cells and organisms [1].

At the epicentre of circadian rhythms is melatonin (MLT) or *N*-acetyl-5-methoxy tryptamine, an indoleamine primarily produced by the pineal gland and secreted into the blood [2, 3]. The indoleamine can be administered exogenously, i.e. orally, as capsules, tablets or liquids, sublingually, or as transdermal patches. It is available without prescription (over-the-counter) in many countries for the treatment of insomnia and depression. MLT synchronises the internal hormonal environment to the light–dark cycle of the external environment and controls circadian rhythms [4, 5]. Unfortunately, at night, artificial lighting such as light-emitting diodes (LED) continues to activate the suprachiasmatic nucleus of the brain, suppressing the natural release of MLT and potentially causing health problems [6]. Previous studies have provided evidence of the role of MLT on the regulation of circadian rhythms as well as its connection with the development of various cancers (breast, prostate, endometrial, ovary, colorectal and skin), cardiovascular diseases, gastrointestinal and digestive problems, diabetes, obesity, depression, sleep deprivation, premature ageing and cognitive impairment [7–16].

A comprehensive, informed and up-to-date review of the current knowledge on the effects of MLT on health is not only timely but urgent, given the technological and lifestyle changes, e.g. chronodisruption, following the overwhelming use of the LEDs omnipresent in computers, smartphones and tablets.

Therefore, the objectives of this umbrella review were to evaluate the evidence for the effects of MLT on health from the published literature, specifically systematic reviews (SRs) and narrative reviews (NRs), to investigate the potential mechanisms of action and to identify which health outcomes are associated with the production and/or supplementation of MLT.

## Methods

The Cochrane Handbook for Systematic Reviews of interventions and the Preferred Reporting Items for Systematic Reviews and Meta-Analyses (PRISMA) guidelines [17] were adhered to while writing and reporting this review (Prospero registration number: CRD42016039840; available at www.crd.york.ac.uk/PROSPERO) [18].

### Literature search and eligibility criteria

For the electronic search, the following databases were searched for entries from January 1996 until July 2017: MEDLINE (via Ovid), EMBASE (via Ovid), Web of Science, CENTRAL (Wiley), PsycINFO (Ovid) and CINAHL (via EBSCO). We hypothesised that any significant reviews or studies would have been captured by reviews conducted since January 1996 (our search start date). A detailed search strategy for MEDLINE is presented in the Appendix. In addition to the electronic searches, the reference lists of all eligible articles were reviewed for further potentially relevant studies. Only data from the published papers were used; the study authors were not contacted.

We included SRs (defined as research articles with a replicable methods section, e.g. searches, eligibility criteria and critical appraisal of primary studies) [19] or NRs (defined as articles without a replicable methods section) [20] of studies involving both healthy and ill individuals of any age and gender using both endogenous and exogenous MLT and MLT agonists. Reviews that relied on data from animal, human or/and in vitro studies with any type of health-related outcome measures were eligible. All SRs and NRs that are for the same associations throughout the search period regardless of the amount and level of overlap, i.e. one primary study included in two or more reviews and/or two or more identified reviews on the same topic, were eligible. We excluded reviews of plants, abstracts or review protocols and reviews not published in English.

### Study selection

The data screening and selection process were performed by the first reviewer (PP) and verified and validated by a second reviewer (BMK). All identified references were imported into EndNote (X7.7.1). The search results from all the bibliographic searches were merged and duplicate records removed.

### Data extraction

Working in groups of two, four authors (BMK, UD, GD and SB) independently extracted relevant information from the studies included using a custom-made data extraction form. The data were subsequently validated by a fifth author (PP). The following information was extracted from the reviews included: first authors’ names and publication date, total number of primary studies, total number of patients included, quality of SRs (Oxman checklist score), quality of primary studies (low, moderate or high as determined by the authors of the reviews), subject/condition/indication, administration of MLT (dose, route, frequency and duration), details of any meta-analyses (MAs), health outcomes/effects/overall results, confounders, and any additional comments. Any disagreements were resolved by discussion between the authors.

### Quality assessment

The methodological quality of SRs was independently evaluated by five reviewers using the Oxman checklist [21]. This validated tool assesses the quality of review articles across nine domains: (1) reporting of search strategy, (2) comprehensiveness of searches, (3) repeatable eligibility criteria, (4) avoidance of selection bias, (5) presence of a validity assessment tool, (6) use of the validity assessment tool, (7) robustness of data analysis, (8) appropriateness of data analysis and (9) supportiveness of conclusions. Each question was scored as 1 (fulfilled), 0 (partially fulfilled) or -1 (not fulfilled). A score of 1 or below indicates extensive flaws, 2–3 indicates the presence of major flaws, 4–5 means minor flaws and 6–9 indicates minimal or no flaws. Again, any disagreements (*N* = 6) were resolved by discussion between the authors.

### Statistical analysis

The results from NRs or SRs that did not pool data quantitatively (*N* = 164) are presented narratively using descriptive tables. Sub-group analyses were conducted for the subset of 31 SRs that had pooled their data quantitatively. For that purpose, the approach by Bellou et al. [22] was used. For each health outcome, we calculated the number of participants and original studies involved in the MA, summary effect sizes [with 95% confidence intervals (CI) and *P* values] using both random- and fixed-effects models. The 95% prediction interval (PI) was calculated, which further accounts for between-study heterogeneity and estimates the uncertainty around the effect that would be anticipated in a new study evaluating that same association. Between-study heterogeneity was measured with the *I*^2^ statistic. An *I*^2^ value of 50% or more is considered to represent a substantial level of heterogeneity, whereas values exceeding 75% are considered to represent considerable heterogeneity. These values also need to be interpreted in light of the size and direction of effects and the strength of the evidence for heterogeneity, based on the *P* value from Cochran’s Q test [18]. The evidence of small-study effects (i.e. the tendency of smaller studies to produce substantially larger effect size estimates compared to larger studies) was evaluated by Egger’s regression asymmetry test [23]. In a more conservative way, a *P* value less than 0.10 from Egger’s test was considered to be evidence of small-study effects. Wherever possible, we extracted the estimate of the largest study (with least standard error) of each MA from a random-effect model to interpret the direction and magnitude of the effect size. We characterised the convincing associations if they met the following criteria: had significance according to a random-effects meta-analysis of less than 0.001, were based on greater than 1000 participants, had between-study heterogeneity (*I*^2^) < 50% and a 95% PI excluded the null value, and had no evidence of small-study effects and excess significance bias. MAs where the required information was not available were excluded from mainstream analyses and presented in a separate table. The statistical analyses were done with open-source R software (version 3.3.1) for Windows using the Meta package. The Pieper et al. formula [24] was used for calculating the amount of overlap (as a percentage) of primary trials in the included SRs (i.e., corrected covered area). A corrected covered area within the range 0–5% indicates a slight overlap, 6–10% indicates a moderate overlap, 11–15% indicates a high overlap and > 15% indicates a very high amount of overlap.

## Results

**Fig. 1:**
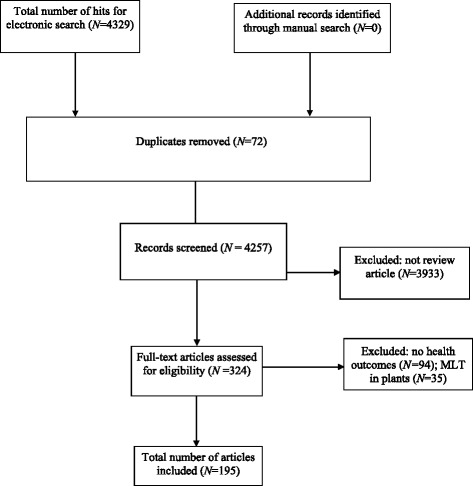
Flow diagram for studies included. MLT melatonin

**Fig. 2:**
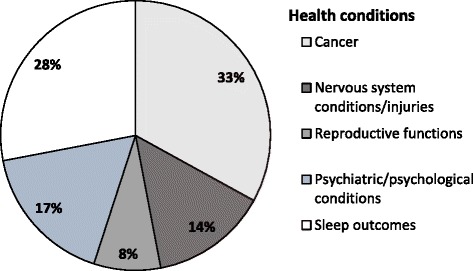
Health conditions with more than ten systematic reviews

**Fig. 3:**
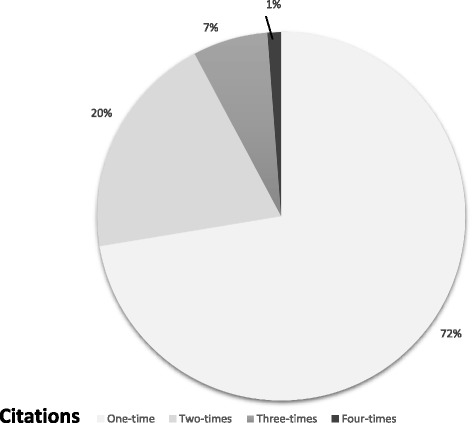
Distribution of citations of different RCTs in the subset of 31 SRs and MAs included. MA meta-analysis, RCT randomised controlled trial, SR systematic review

Our searches identified a total of 4329 records; 195 review articles met the inclusion criteria (Fig. 1). Table 1 presents the biological mechanisms of action involved. Tables 2 and 3 summarise MAs of MLT for health with and without sufficient data for quantitative synthesis, respectively. Table 4 summarises reviews with overlapping conditions (Fig. 2). The key data from the included SRs or NRs are summarised in Additional file 1: Table S1 and Additional file 2: Table S2. Additional file 3: Table S3 gives the methodological quality of the papers included. Additional file 4: Table S4 lists all randomised controlled trials (RCTs) covered in the subset of 31 SRs and indicates the amount of overlap (Fig. 3). Additional file 5: Table S5 lists adverse effects (AEs) reported in SRs. Altogether, 31 reviews were synthesised quantitatively, whereas the remaining 164 reviews were synthesised narratively.

**Table 1:** Biological functions and processes that may be affected by MLT and suggested mechanisms of action in various models

Function or process	Effects	Suggested mechanisms	Type of evidence (references)
Cancer	Tumour regression; activation of tumour-suppressive signalling network; oncostatic activity; modulation of oestrogen and androgen; immunomodulation or neuroimmunomodulation; cytoskeletal modulation; modulation of water transport; resynchronisation of the intracellular clock network; modulation of cellular redox status; haematopoiesis; reduced cardiotoxicity; enhanced mitochondrial function; anti-oestrogen; epigenetic regulation; radioprotection	Reduction of cellular proliferation; free radical scavenging; inhibition of the uptake of linoleic acid; stimulation of glutathione production (γ-glutamylcysteine synthase and reduced reactants such as hydroxyl radical, hydrogen peroxide, hypochlorous acid, singlet oxygen, the peroxynitrite anion and peroxynitrous acid); blocking cell-cycle progression from the G phase to the S phase and by increasing p53, p21 and p27^Kip1^ gene and protein expression (via increased expression of E-cadherin and β1-integrin proteins); stimulation of lymphocytes, monocytes, granulocytes, macrophages, T-helpers (Th1 and Th2), T and B lymphocytes and thrombocytes; NK cell activity; platelet generation; enhancement of the production of cytokines IL-1, IL-2, IL-4, IL-6, IL-10, IL-12, IL-24, IFN-γ and TNF-α; co-activation of protein kinase C and protein kinase A, and phospholipase C; inhibition of angiogenesis (via inhibition of endothelin-converting enzyme-1 and insulin-like growth factor 1); cell apoptosis; inhibition of 17β-oestradiol; stimulation of biopterins; microfilament modulation; switching microfilament phenotypes; improving oxidative phosphorylation and increasing ATP generation; reduced electron leakage and mPTP opening; decrease in gonadal steroids; downregulation of the expression of oestrogen α receptors; potentiation of cytostatic anti-oestrogen sensitivity of chemotherapeutic agents; inhibition of DNA methyltransferase; inhibition of telomerase; inhibition of metastasis; mutations in the melatonin receptors (MLT1 and MLT2); alterations of arylalkylamine *N*-acetyltransferase; reduced thrombocytopenia; inhibition of prostaglandin E2; inactivation of calmodulin	In vitro, animal and clinical studies, RCTs, epidemiological studies, SRs [31, 37, 44, 63, 68, 69, 72–108]
Metabolic and cardiovascular disorders	Anti-oxidative; anti-inflammatory; anti-hypertensive; regulation of lipid and glucose metabolism; reduction of nephrotoxicity	Free radical scavenging; inhibition of pro-inflammatory mediator; iNOS/i-mtNOS; optimisation of nNOS/c-mtNOS; reduction of factor 1-α and NF-κB; downregulation of Bcl-2 and activation of p53 and CD95; increase in catalase activity and reduction in thiobarbituric acid reactive substrates; reduction in lipid peroxidation, creatinine, uric acid and blood urea nitrogen levels	In vitro, animal studies, placebo-controlled RCTs [80, 90, 92, 106, 109–114]
Gastrointestinal conditions	Anti-oxidative; anti-inflammatory	Free radical scavenging; inhibition of pro-inflammatory cytokines, cell adhesion molecules, NO production, COX-2 expression, NF-κ activation; regulation of macrophage activity	Animal studies, RCTs [50, 79–81, 92, 114, 115]
Neonatology and paediatrics	Anti-inflammatory; anti-oxidative; sedative	Reduction of pro-inflammatory cytokines (IL-6, IL-8 and TNF-α) and nitrite/nitrate levels; inflammatory-derived activation of phospholipase A2, lipoxygenase and cyclooxygenases; increased glutathione peroxidase activity; reduction of C-reactive protein	Animal and human studies, RCTs, open-label [116–119]
Neurodegenerative disorders	Protection against neurodegeneration caused by mitochondrial dysfunction and oxidative/nitrosative stress; apoptosis; prevention of vasoconstriction of cerebral arteries	Activations of mitochondrial cell survival pathways; regulation of apoptosis; silencing of the Rip2/Caspase-1 pathway; reduced mitochondrial inducible NO synthase; increased activity of respiratory complexes I, III and IV; increased activity and expression of antioxidant enzymes; high lipophilicity	Animal and human studies, SRs [46, 49, 79, 81, 90, 94, 100, 106, 120–124]
Mental disorders	Anti-inflammatory; anti-nociceptive; anxiolytic; drug detoxification	Regulating cytokine production of immunocompetent cells; reducing adhesion molecules and pro-inflammatory cytokines including IL-6, IL-8 and TNF; modifying serum inflammatory parameters; neutralising free radicals and non-radical oxygen-based reactants	Animal and human studies [34, 66, 123, 125–130]
Pain syndromes	Anti-nociceptive, antiallodynic and analgesic effects; synchronisation of biological rhythms	Activation of melatoninergic MLT1/MLT2 receptors; release of opioid peptides (β-endorphins); interaction with opioid, γ-aminobutyric acid or *N*-methyl-daspartate receptors; NO-arginine pathway; antioxidant and anti-inflammatory effect; regulation of endoplasmic reticulum and mitochondrial activity	Animal and human studies [33, 117, 131, 132]
Reproductive functions	Antioxidant, anti-inflammatory, anti-apoptotic, cytoprotective and neuroprotective effects; reduced risk of complications; increased homeostasis; gonadotropin secretion; higher rate of mature oocytes and quality embryos	Activation of melatoninergic MLT1/MLT2 receptors; inhibition of adenyl cyclase activity; forskolin-induced cAMP formation with subsequent reduction in activated protein kinase; alteration of granulosa cell steroidogenesis and folliculogenesis; corpus luteum function; inhibition of prostaglandins, oxytocin, cortisol production and LDL peroxidation; activation of prolactin secretion; free hydroxyl radicals scavenging; prevention against DNA damage; activation of superoxide dismutase, glutathione peroxidase, glutathione reductase and glucose-6-phosphate dehydrogenase; inhibition of NO synthase; deferred apoptosis of villous cytotrophoblasts and protection of syncytiotrophoblasts; improved haemodynamics and nutrient transfer at the placental-uterine interface	In vitro, animal and human studies [62, 95, 115, 119, 133–139]
Sleep disorders	Sleep enhancer; shifted circadian rhythms; reduced duration of jet lag	Activation of alpha-2 noradrenergic receptor agonist clonidine; lowered core body temperature; opening of the sleep gate and facilitation of re-entrainment to suprachiasmatic nuclei; potentiation of GABA on GABA_A_ receptors; inactivation of calmodulin	RCTs [26, 29, 39, 42, 64, 67, 70, 80, 81, 90, 92, 94, 103, 105–107, 124, 140–161]
Traumatic CNS injury	Attenuation of neural damage; neuroprotective effects; inhibition of necrosis, apoptosis; immunomodulation; protection of nuclear and mitochondrial DNA; anti-oxidative effects	Free radical scavenging (including the hydroxyl radical, hydrogen peroxide, singlet oxygen, NO, peroxynitrite anion and peroxynitrous acid); inhibition of pro-inflammatory cytokines or quinone reductase 2, calcium ion-mediated toxicity, proxidative enzymes NO synthase, lipoxygenase and phospholipase A2; activation of the tumour necrosis factor receptors; increased efficiency of oxidative phosphorylation; reduction of NF-κB or TNF expression; modulation of angiogenesis; stimulation of superoxide dismutase, glutathione peroxidase, glutathione reductase, catalase and glutathione; induction of γ-glutamylcysteine synthetase; activation of glucose-6-phosphate dehydrogenase	In vitro, animal and human studies [27, 32, 46, 61, 94, 106, 162–168]

*ATP* adenosine triphosphate, *cANP* cyclic adenosine monophosphate, *c-mtNOS* constitutive mitochondrial nitric oxide synthase, *CNS* central nervous system, *COX-2* cyclooxygenase 2, *GABA* gamma-aminobutyric acid, *iNOS* inducible nitric oxide synthase, *i-mtNOS* inducible mitochondrial nitric oxide synthase, *LDL* low-density lipoproteins, *MLT* melatonin, *NF-κB* nuclear factor kappa-light-chain-enhancer of activated B cells, *NK* natural killer, *nNOS* neuronal nitric oxide synthase, NO nitric oxide, *mPTP* mitochondrial permeability transition pore, *RCT* randomised controlled trial, *SR* systematic review, *TNFα* tumour necrosis factor α

**Table 2:** Characteristics and quantitative synthesis of the eligible MAs of MLT for health

First author (year) [reference]	Health outcome	No of participants included in MA	No of primary studies included in MA	Reported effect size	Random-effects summary effect size (95% CI)	*P* random	Fixed-effects summary effect size (95% CI)	*P* fixed	95% PI	*I*^2^ (%)	Estimate of the study with lowest SE in MA (95% CI)^1^	Small-study effects/excess statistical significance
Addiction												
Wright^2^ (2015) [36]	Withdrawal symptoms	244	4	OR	1.39 (0.42, 4.65)	0.594	1.11 (0.65, 1.90)	0.694	0.01, 277.67	75.9	1.00 (0.37, 2.67)	No/No
Cancer												
Basler (2014) [68]	Risk of breast cancer	1650	5	RR	0.82 (0.68, 0.99)	0.043	0.82 (0.71, 0.95)	0.01	0.50, 1.34	31.7	0.81 (0.64, 1.02)	No/No
Wang (2012) [31]		761	8	RR	1.95 (1.49, 2.54)	0.0001	1.96 (1.50, 2.56)	0.0001	1.40, 2.71	0	2.25 (1.39, 3.64)	No/No
Wang (2012) [31]		590	5	RR	1.90 (1.28, 2.83)	0.0002	1.82 (1.49, 2.24)	0.0001	0.56, 6.47	61.9	1.26 (0.97, 1.65)	No/No
Delirium												
Chen (2015) [35]	Incidence of delirium	669	4	RR	0.41 (0.15, 1.13)	0.084	0.73 (0.55, 0.95)	0.021	0.01, 35.8	83.8	1.16 (0.83, 1.61)	Yes/No
Dementia												
Jansen^3^ (2009) [30]	Cognition	121	2	MD	–2.64 (–5.99, 0.71)	0.123	–2.12 (–3.82, 0.42)	0.015	–	68.6	–1.15 (–3.16, 0.86)	–/No
Jansen (2009) [30]	Mood and behaviour	150	3	MD	0.18 (–0.73, 1.10)	0.698	0.18 (–0.73, 1.10)	0.698	–5.76, –6.12	0	–0.01 (–1.08, 1.06)	No/No
Depression/mood disorders												
De Crescenzo (2017) [127]	Mood disorders	181	3	SMD	0.37 (–0.05, 0.78)	0.087	0.39 (0.08, 0.70)	0.013	–3.74, 4.47	43	0.32 (–0.14, 0.78)	No/No
Guaiana (2013) [34]	Response to treatment	3826	10	RR	1.01 (0.95, 1.08)	0.749	1.00 (0.95, 1.06)	0.881	0.87. 1.17	31.4	1.04 (0.93, 1.15)	No/Yes
Huang (2014) [65]		1871	6	RR	1.07 (1.02, 1.13)	0.01	1.08 (1.03, 1.15)	0.005	0.99, 1.16	0	1.04 (0.93, 1.15)	No/No
Guaiana (2013) [34]	Remission	3826	10	RR	0.83 (0.68, 1.02)	0.069	0.87 (0.80, 0.94)	0.0007	0.43, 1.59	77.8	0.95 (0.85, 1.06)	No/No
Huang (2014) [65]		1742	5	RR	1.11 (1.01, 1.23)	0.035	1.12 (1.01, 1.24)	0.038	0.95, 1.31	0	1.09 (0.93, 1.27)	No/No
Hansen^3^ (2014) [65]	Hospital Anxiety and Depression Scale	74	2	MD	0.97 (–0.84, 2.78)	0.293	0.93 (–0.42, 2.28)	0.178	–	44	0.10 (–1.72, 1.92)	–/No
Hansen^3^ (2014) [65]	Beck Depression Inventory	91	2	MD	–1.09 (–2.60, 0.42)	0.157	–1.09 (–2.60, 0.42)	0.157	–	0	–1.00 (–2.54, 0.54)	–/No
Infertility												
Seko (2014) [25]	Pregnancy rate	680	5	RR	1.21 (0.98, 1.49)	0.071	1.21 (0.98, 1.50)	0.071	0.86, –1.70	0	1.13 (0.85, 1.51)	No/No
Seko (2014) [25]	Oocytes retrieved	680	5	MD	0.57 (–0.22, 1.35)	0.155	0.23 (–0.12, 0.8)	0.2	–1.91, –3.04	68.7	–0.07 (–0.57, 0.43)	Yes/No
Pre- and post-operative care												
Andersen (2014) [33]	Pre-operative anxiety	761	11	SMD	–0.88 (–1.33, –0.44)	<0.0001	–0.91 (–1.07, –0.75)	<0.0001	–2.53, –0.76	86.7	0.00 (–0.33, 0.33)	No/No
Hansen^3^ (2015) [169]		122	2	MD	–1.18 (–2.59, 0.23)	0.1	–1.18 (–2.59, 0.23)	0.1	–	0	–1.30 (–2.76, 0.16)	–/No
Hansen^3^ (2015) [169]	Post-operative anxiety	73	2	MD	–5.31 (–8.78, –1.84)	0.003	–5.31 (–8.78, –1.84)	0.003	–	0	–5.40 (–10.12, –0.68)	–/No
Andersen (2014) [33]	Post-operative pain	524	8	SMD	–1.06 (–1.89, –0.24)	0.012	–0.12 (–0.30, 0.07)	0.205	–3.97, 1.85	94.2	0.43 (0.09, 0.77)	Yes/No
Mihara (2015) [41]	Prevention of agitation	170	3	RR	0.31 (0.16, 0.60)	<0.0001	0.29 (0.15, 0.56)	<0.0001	0.00, 23.06	0	0.40 (0.18, 0.89)	No/Yes
Safety												
Liu (2012) [40]	Adverse effects	2912	7	RR	1.10 (1.02, 1.20)	0.009	1.11 (1.03, 1.20)	0.006	1.02, 1.20	0	1.13 (0.97, 1.31)	No/Yes
Primary sleep disorders												
Liu^3^ (2012) [40]	Sleep latency	405	1 (with six subgroups)	MD	–14.26 (–18.54, –9.98)	<0.0001	–14.26 (–18.54, –9.98)	<0.0001	–20.32, –8.19	0	–16.70 (–26.82, –6.58)	–/NE
Kuriyama (2014) [39]		5781	12	WMD	–4.15 (–6.82, –1.47)	0.002	–3.30 (–4.88, –1.71)	<0.0001	–11.69, 3.39	52.2	–2.40 (–5.28, 0.48)	No/No
Liira (2014) [38]		148	5	MD	–0.15 (–2.48, 2.18)	0.899	–0.41 (–2.32, 1.50)	0.674	–5.64, 5.34	21.6	–1.10 (–3.83, 1.63)	Yes/No
Liira (2014) [38]		266	7	MD	24.30 (9.80, 38.80)	0.001	24.30 (9.80, 38.80)	0.001	5.29, 43.32	0	23.00 (–3.13, 49.13)	No/No
Kuriyama (2014) [39]	Sleep quality	5812	13	SMD	–0.08 (–0.13, –0.03)	0.003	–0.08 (–0.13, –0.03)	0.003	–0.14, –0.02	0	–0.15 (–0.27, –0.02)	No/No
Zhang^3^ (2016) [157]		18	1	MD	4.20 (0.92, 7.48)	0.012	4.20 (0.92, 7.48)	0.012	–	–	4.20 (0.92, 7.48)	–/No
Animal studies												
Yang (2016) [32]	Spinal cord injury	90	6	MD	1.52 (0.06, 2.98)	0.041	1.29 (0.82, 1.77)	<0.0001	–3.69, 6.73	89	0.16 (–0.88, 1.20)	Yes/No

*CI* confidence interval, *MA* meta-analysis, *MD* mean difference, *MLT* melatonin, *NE* not estimable, *OR* odds ratio, *PI* prediction interval, *RR* risk ratio, *SE* standard error, *SMD* standardised mean differences, *WMD* weighted mean differences

^1^Estimate of the largest study with lowest SE from random-effect model

^2^Estimates did not match with forest plot in the article

^3^The 95% prediction interval and the evidence of small-study effects were calculated for those MAs where ≥3 studies combined (it cannot be calculated for less than three studies as degrees of freedom will be zero for two studies and negative for one study)

**Table 3:** Characteristics of the eligible MAs of MLT for health (with insufficient data for quantitative synthesis)

First author (year) [reference]	Health outcome	No of participants included in MA	No of primary studies included in MA	Reported effect size	Random-effects summary effect size (95% CI)	*P* random	Fixed-effects summary effect size (95% CI)	*P* fixed	95% PI	*I*^2^ (%)	Estimate of the study with lowest SE in MA (95% CI)^1^	Small-study effects/excess statistical significance
Cancer												
Yang (2014) [70]	Risk of breast cancer	4550	5	RR	0.86 (0.78, 0.95)	–	–	–	–	46.4	–	–
Mills (2005) [68]	Risk of death at 1 year	643	10	RR	0.66 (0.59, 0.73)	–	–	–	–	0.0	0.64 (0.52, 0.78)	No
Seely^2^ (2012) [37]		–	13	RR	0.63 (0.53, 0.74)	<0.001	–	–	–	78.0		
Seely^2^ (2012) [37]	Complete response	–	12	RR	2.33 (1.29, 4.20)	–	–	–	–	–	–	–
Seely^2^ (2012) [37]	Partial response/remission	–	16	RR	1.90 (1.43, 2.51)	–	–	–	–	–	–	–
Seely^2^ (2012) [37]	Stable disease	–	12	RR	1.51 (1.08, 2.12)	–	–	–	–	–	–	–
Nocturnal hypertension												
Grossman (2011) [57]	SBP	72	3	MD	–6.10 (–10.69, 1.50)	0.009	–	–	–	–	–8.00 (–15.02, 0.97)	–
Grossman (2011) [57]	DBP	72	3	MD	–3.51 (–6.14, 0.86)	0.009	–	–	–	–	–3.90 (–7.68, 0.11)	–
Primary sleep disorders												
Braam (2009) [169]	Sleep latency	170	7	MD	–33.8 (–42.97, –24.70)	*p* < 0.01	–	–	–	–	–32.7 (–47.55, –17.85)	–
Brzezinski (2005) [28]		177	11	MD	–	–	4.0 (2.5, 5.4)	–	–	–	–	–
Buscemi (2005) [154]		279	14	WMD	–11.72 (–18.24, –5.20)	0.0004	–	–	–	81.6	0.30 (–0.70, 1.30)	–
Ferracioli-Oda (2013) [29]		1468	15	WMD	10.18 (6.1, 14.27)	<0.001	7.06 (4.37, 9.75)	<0.001		56.0	1.00 (–3.57, 5.57)	–
Van Geijlswijk (2010) [161]		317	9	MD	–23.27 (–41.72, –4.83)	0.013	–	–	–	–	–	–
Braam (2009) [169]	Total sleep Time/duration	183	9	MD	0.83 (0.57, 1.08)	*p* < 0.01	–	–	–	–	1.09 (0.70, 1.48)	–
Brzezinski (2005) [28]		112	8	MD			12.8 (2.9, 22.8)	–	–	–	–	–
Ferracioli-Oda (2013) [29]		1016	13	WMD	8.48 (–4.02, 20.98)	0.184	8.25 (1.75, 14.75)	0.013		44.0	7.80 (–0.69, 16.29)	–
Rossignol^3^ (2011) [143]		–	5	Hedge’s *g*	1.97 (1.10, 2.84)	<0.001	–	–	–	–	–	–
Van Geijlswijk (2010) [161]		304	9	MD	–0.67 (–0.89, –0.45)	<0.0001	–	–	–	–	–	–
Braam (2009) [169]	Number of wakes per night	183	9	MD	–0.16 (–0.30, 0.02)	0.024	–	–	–	–	–0.18 (0.35, –0.01)	–
Brzezinski (2005) [28]	Sleep efficiency	126	7	MD	–	–	2.2 (0.2, 4.2)	–	–	–	–	–
Van Geijlswijk (2010) [161]	Dim-light melatonin onset	238	6	MD	–1.18 (–1.48, –0.89)	<0.0001	–	–	–	–	–0.87 (–1.37, –0.37)	–
Van Geijlswijk (2010) [161]	Wake-up time	195	5	MD	–0.28 (–0.66, 0.09)	0.135	–	–	–	–	–0.20 (–0.45, 0.06)	–
Secondary sleep disorders												
Buscemi (2006) [154]	Sleep onset latency	163	6	MD	–13.22 (–27.33, 0.89)	0.070	2.30 (–0.13, 6.12)	0.060	–	79.2	5.8 (2.47, 9.13)	–
Buscemi (2006) [154]	Sleep restriction	508	9	MD	–0.97 (–2.26, 0.33)	0.140	–0.89 (–1.98, 0.20)	0.110	–	4.0	–1.05 (2.30, 0.20)	–
Miscellaneous												
Marrin (2013) [170]	Core temperature	193	16	MD	–0.21 (–0.24, –0.18)	<0.001	–	–	–	–	–	–
Animal studies												
Macleod (2004) [27]	Ischaemic stroke	432	13	ES	0.43 (0.39, 0.64)	<0.0001	–	–	–	–	–	–

A dash indicates the data are not estimable or extractable from SRs

*CI* confidence interval, *ES* effect size, *MA* meta-analysis, *MD* mean difference, *MLT* melatonin, *OR* odds ratio, *PI* prediction interval, *RR* risk ratio, *SE* standard error, *SMD* standardised mean differences, *WMD* weighted mean differences, *SBP* systolic blood pressure, *DBP* diastolic blood pressure

^1^Estimate of the largest study with lowest standard error from random-effect model

^2^Number of participants is not extractable in the article

**Table 4:** Reviews with overlapping conditions

Subjects/condition/health outcome/indication	Number of systematic reviews (*N*)
Ageing	5
Cancer	43
Cardiovascular	9
Delirium	2
Epilepsy	2
Excretory/renal functions	2
Gastrointestinal function/conditions	7
Healthy adults	6
Infections (various)	6
Inflammatory conditions	10
Menopause (symptoms)	2
Musculoskeletal system	3
Neonates, infants and children (various conditions)	9
Nervous system (central and peripheral) conditions/injuries	18
Neurodegenerative disorders/dementias	10
Obesity/metabolic diseases	10
Other (miscellaneous)	6
Oral cavity diseases	3
Pain syndromes	5
Pregnancy/reproductive functions/infertility	11
Pre-operative, peri-operative or post-operative care (anxiety, prevention of agitation)	4
Protection against radiation/metal toxicity	4
Psychiatric/psychological conditions	22
Sleep outcomes/insomnia	37
Various clinical conditions	10

### Characteristics of studies included (*N* = 195)

The number of primary studies in each SR ranged from 0 to 68 (mean 6.5 ± 10.78). The total number of participants was inestimable due to overlapping studies (optional range 61 to 5812). In 117 of the reviews (60%), either the number of primary studies or the number of participants was not available. None of the included SRs or MAs had access to individual participant data and all relied on summary-level data from the published literature. Eighteen SRs relied on continuous data for their respective MAs [standardised mean difference (SMD), mean difference (MD) and weighted mean difference (WMD)]; and 12 (6.1%) used dichotomous data for pooling [odds ratio (OR) and risk ratio (RR)]; with only one MA using both types of data and analyses (RR and MD) [25]. Three MAs used effect sizes for presenting the overall estimates [26–28].

Various conditions were evaluated, ranging from acute coronary syndrome to various cancers, with insomnia/sleep disorders being the most frequent (*N* = 50; 25.6%). Of these, 26 focused on insomnia/primary sleep disorders only, whereas the remaining 24 evaluated other health conditions with underlying (secondary) sleep disorders. Four reviews (2%) included healthy individuals; and six (3%) evaluated a mixture of healthy and unhealthy patients. Human studies varied from case studies (*N* = 4), case series (*N* = 4), case control (*N* = 2), cohort (*N* = 1), open-label (*N* = 13) and uncontrolled before–after (*N* = 2) to RCTs of parallel and cross-over design with or without the use of a placebo (*N* = 71).

Administration routes varied from oral and intravenous to sublingual; and MLT preparations included patches, pills, capsules and solutions. In total, 99 reviews (50.7%) included animal/in vivo studies and 55 reviews (28.2%) also included in vitro studies, whereas 84 reviews (43%) included humans only. Confounding factors were not mentioned in 82 reviews (42%). In the remaining 113 reviews, both exogenous and endogenous MLT levels were influenced by a range of genetic, epigenetic and environmental factors including age, gender, menopausal status, parity, oestrogen levels, lifestyle (alcohol use, body mass index, body posture, caffeine, diet, supplements, drug use, night-shift work, artificial light at night, physical activity, psychological stress and sleep hygiene) and others, including individual chronotypes, sessional variations and time, dose and route of MLT administration. In medically compromised patients, e.g. those with cancer, MLT was frequently used as an adjunct to usual care or conventional treatment such as chemotherapy, radiotherapy, supportive care and palliative care.

The most commonly cited effects of MLT were its anti-oxidative, anti-inflammatory and immunomodulatory properties (Table 1). In neoplastic diseases, the most common mechanisms of action included free radical scavenging (hydroxyl radical, hydrogen peroxide, hypochlorous acid, singlet oxygen, the peroxynitrite anion and peroxynitrous acid); stimulation of immune system; improvement of oxidative phosphorylation and ATP generation; co-activating protein kinase enzymes; reduction of cellular proliferation; inhibition of angiogenesis; prostaglandin E2 or 17β-oestradiol; the uptake of linoleic acid, DNA methyltransferase or telomerase.

### Evaluation of the evidence

Four MAs [25, 29–31] had large levels of heterogeneity (*I*^2^ ≥ 50% and ≤ 75%) and six SRs [32–37] had very large levels of heterogeneity (*I*^2^ > 75%). The median number of studies per MA was 5 (IQR = 4.75) with a median of 557 participants (IQR = 1561). In each of the 13 MAs, more than 1000 cases were analysed. For sleep latency, pre-operative anxiety, prevention of agitation or risk of breast cancer, ten (32%) of 31 MAs reported effects that were significant at *P* values less than 0.05 under the random-effects model, and seven (23%) were significant at *P* values less than 0.001 under the random-effects model [31, 33, 38–41]. For eight MAs (25.8%), we were unable to calculate 95% PIs. The remaining 23 MAs had a 95% PI that included the null value, meaning that, although on average MLT improves various health outcomes, this might depend on dose, duration, intensity, age, gender or underlying co-morbidities. Evidence for small-study effects was noted in three MAs (9.6%). These MAs pertained to the incidence of delirium [35], spinal cord injury [32] or post-operative pain [33] (Table 2).

Only one review [39] for the association of MLT and sleep quality met our predefined convincing association criterion. It highlighted that ramelteon can improve sleep quality in insomnia (SMD = -0.08, 95% CI = -0.13 to -0.03). If we reduced the minimum number of participants in an MA to ≥500, then one more review [31] would satisfy the inclusion criterion. It highlighted that melatonin therapy can improve the partial and complete remission of solid tumour cancers (RR = 1.95, 95% CI = 1.49 to 2.54).

### Quality of SRs

The quality of the reviews as measured with the Oxman checklist was typically low (range = -9 to 9; mean = -4.5, SD = 6.7) (Additional file 3: Table S3). Of the reviews included, 153 (153/195; 78.4%) did not use appropriate methods for combining studies and hence were scored as -1.

### Quality (and number) of primary studies

Altogether 154 reviews (78.9%) did not evaluate the methodological quality of the primary studies (no validity assessments). In 41 reviews (21.1%) that did undertake this, the methodological quality of the primary data ranged from poor (*N* = 5) to high (*N* = 13), with moderate being most commonly reported (*N* = 18), as assessed by the Cochrane Risk of Bias Tool or the Jadad Scale. The median number of primary studies included was *N* = 9 (when possible to estimate).

### Melatonin receptor agonists

Melatonin receptor agonists, such as Circadin® (prolonged-release MLT), ramelteon, agomelatine or tasimelteon, bind to and activate the MLT receptors 1 and 2 [42]. These analogues of MLT are believed to have the same mechanisms of action as MLT and are typically used for the treatment of sleep disorders and depression [43]. Two reviews of Circadin (prolonged-release MLT), four of ramelteon, two of agomelatine and one of tasimelteon were included. The duration, intensity and frequency varied across the reviews, with 8 mg being most commonly used in ramelteon studies, 2 mg for Circadin; 25–50 mg for agomelatine and 1–50 mg for tasimelteon.

### Endogenous vs. exogenous MLT

In total, 31 reviews (15.8%) evaluated both exogenous and endogenous MLT. However, it was often difficult to ascertain the number of studies looking at exogenous MLT vs. endogenous MLT only. The exogenous vs. endogenous MLT doses are also incomparable, as the routes of administration and types of studies differed considerably (optional range 0.003 mg to 3 g).

## Discussion

This umbrella review aimed to summarise and critically evaluate the evidence from SRs and NRs of the effects of MLT on health and to identify the biological mechanisms of action involved. In total, 195 reviews were included (96% of the reviews were published after 2000). Of the reviews, 99 included evidence from in vitro or animal experiments, which highlights the still experimental phase of some MLT research and the translational potential for human trials.

There was a considerable clinical and methodological heterogeneity in terms of populations evaluated (from neonates to elderly), doses, excipients, quality or purity of MLT preparations, comparators, outcome measures, study designs, lengths of follow-ups, settings, etc. Despite that, the present review does lend support to the notion that endogenous and exogenous MLT is associated with improved health outcomes. However, caution is advised for the use or supplementation of MLT in some autoimmune conditions, such as rheumatoid arthritis, asthma or organ transplantation as MLT has been reported to stimulate the function of the immune system via the production of interleukins (IL-1, IL-2, IL-6 and IL-12), interferon γ (IFN-γ), T_h_ cells, cytotoxic T cells, and B- and T-cell precursors [44].

Overall, though it seems that the connection between MLT and health is well founded, there is less evidence connecting MLT with specific diseases in a systematic way. The physiological role of MLT, as uncovered by various experimental studies, does, quite robustly, point to a direct relation between MLT and critical elements of health. However, the connection with specific conditions needs to be researched comprehensively. Thus, we suggest the need for high-quality primary data and we underline the importance of targeted studies on specific conditions, such Alzheimer’s or cardiovascular diseases.

### Mechanisms of action

Some of the effects of MLT are via anti-oxidative (e.g. [45–49]), anti-inflammatory (e.g. [50–52]), anti-apoptotic (e.g. [53, 54]), anti-nociceptive (e.g. [33, 55]), anti-hypertensive (e.g. [56–58]), cytoprotective, neuroprotective, cardioprotective or nephroprotective effects (e.g. [59–64]), and by enhancing mitochondrial function and protecting nuclear and mitochondrial DNA or regulating homeostasis (e.g. [53, 65]; Table 1). Even though some of the mechanisms of action are well established, the relative absence of the exact role of confounding factors such as diet, exercise, sleep and genetics on the role of MLT to health limits the generalisability of the results. We here identify three important factors that can be taken into account by future researchers. Firstly, the climatic conditions – and especially latitude – could bias the physiological response. Secondly, the urban environment of cities and the presence of LED light could disrupt circadian rhythms and suppress the production of MLT. Finally, the overall cultural background could also have a significant impact, as this affects nutrition and clothing.

### Safety

AEs of exogenous MLT and MLT analogues were reported in 11 (5.6%) of the included reviews. Two reviews pooled the safety data [40, 66]. In Liu and Wang [40], there were more subjective reports of at least one AE after treatment with ramelteon compared to placebo (RR = 1.11, 1.03 to 1.20, *P* < 0.01; seven studies). In Huang et al. [66], however, agomelatine revealed a lower rate of discontinuation due to AEs compared with selective serotonin reuptake inhibitors or serotonin–norepinephrine reuptake inhibitors (RR = 0.38, 95% CI = 0.25 to 0.57). AEs were typically mild and included worsening of symptoms (seizures, asthma or headaches), transient headaches and dizziness, abdominal pain, pharyngitis, back pain and asthenia, somnolence, fatigue, nasopharyngitis, upper respiratory infection, nausea, dizziness, diarrhoea, dyspepsia, dysmenorrhoea, diarrhoea, dry mouth, increased alanine aminotransferase, nightmares, morning drowsiness, enuresis, rash and hypothermia (Additional file 5: Table S5). Given the overwhelming benefits of MLT treatment and the existence of very few and mild AEs (also for long-term use), the risk–benefit ratio favours MLT.

### Cost-effectiveness

Only two reviews undertook any health economic analysis of MLT. One review stated that the cost of a 30-tablet pack of 2 mg of Circadin was £15.39 [67], whereas Liira et al. [38] ‘did not find evidence on the cost-effectiveness of the drugs in the included trials’. More cost-effectiveness or cost-benefit analyses would be required to confirm the economic benefits of MLT and to inform various stakeholders and policymakers.

### Quality (and quantity) of primary data

In 154 (78.9%) of the reviews, the quality of the primary data was not evaluated. In the 41 reviews (21%) that did evaluate it, the quality of the primary data ranged from poor to high (average = moderate), as judged by the authors of the included reviews, primarily using the Cochrane Risk of Bias Tool. The relatively low number of primary studies (median 9) included in the SRs or NRs might be of potential concern, and signals the need for more research into a wide range of conditions and clinical areas including oncology, emergency medicine, neurology, metabolic diseases, cardiovascular medicine, gynaecology, paediatrics, psychiatry, mental health, gastrointestinal diseases and pain management.

### Review quality

The methodological quality of the included SRs was frequently poor (Additional file 3: Table S3). Most of the articles that scored poorly on the Oxman checklist (quality rating scale) were NRs, which are often of poorer quality compared to SRs. As these articles do contribute relevant information, we decided to include them in our study. Of the reviews, however, 36 (18.4%) scored 6–9 on the Oxman checklist, meaning they had minimal or no flaws.

### Strengths and weaknesses

This umbrella review has important strengths, such as the inclusion and critical appraisal of 195 review articles, identification of gaps and uncertainties in the evidence base, and categorisation of significant health-related effects and associated mechanisms of action. However, this umbrella review of both SRs and NRs has several limitations that ought to be kept in mind when interpreting its results. First and foremost, even though comprehensive searches were employed, there is no guarantee that all relevant SRs of MLT were included. The searches were restricted to the past 21 years, thereby omitting some potentially older and potentially important reviews, as well as reviews published in languages other than English.

Secondly, one of the limitations of our overview is that many SRs often analysed the same primary studies. This overlap between SRs is important when interpreting results of this overview (Additional file 4: Table S4, Fig. 2). For instance, due to the double counting of the patient data resulting from the overlapping studies, the total number of patients included in our analyses is inestimable. Also, in the subset of 31 MAs, 238 RCTs were included. These RCTs were frequently used in more than one MA (range = 1–4, mean = 1.4, SD = 0.66), meaning that there were overlapping studies and double counting of the data (Fig. 2). To further illustrate this, three [31, 37, 68] of five MAs [31, 37, 68–70] evaluating MLT for cancers relied on the same data from the same four primary trials (Lissoni 1996, 1997, 1999, 2003). However, the amount of overlap was calculated (corrected covered area) and found to be 1.2%, which is 'slight' according to Pieper's formula.

Thirdly, although, four SRs were methodologically sound (Oxman checklist score ≥ 6), they were based on poor-quality primary data, which (logically) might seem contradictory.

Fourthly, we did not evaluate whether there was evidence for small-study effects using funnel plot asymmetry [23] (publication bias) because of insufficient data.

Fifthly, reviewing SRs might abandon the nuances that may be embedded in the original data, such as conflicts of interest, sources of funding, validity, generalisability etc.

Sixthly, various animal, human and in vitro models; different modes of administration; and exogenous and endogenous MLT were frequently analysed together, thereby giving limited understanding of how the results vary depending on the health outcomes evaluated.

Lastly, there is no commonly accepted cut-off point differentiating NRs vs. SRs using the Oxman scoring system. For example, a review that arbitrarily scored 2–3 on the scale (indicating the presence of major flaws) may be arbitrarily assigned as an NR as well as an SR (the definition being arbitrary too). In another example, reviews that could be arbitrarily judged as narrative with extensive flaws (a score of 1 or below), e.g. De Jonghe et al. [71], may include information about the number of primary studies and total sample size, i.e. 9/330. On the other hand, reviews that had no flaws (a score of 6–9) may not have that information, e.g. Liira et al. [38]. Taken together, these limitations reduce the conclusiveness of our findings, making them prone to criticism.

## Conclusions

Despite the abundance of evidence, more systematic research is needed to understand and establish the connection between MLT and specific aspects of health, potentially as a function of important lifestyle choices.

## Additional files

Additional file 1: Table S1. Summary of studies on the effects of exogenous melatonin on health outcomes (*N* = 120). (DOCX 174 kb)

Additional file 2: Table S2. Summary of studies on the effects of endogenous melatonin on health outcomes (*N* = 75). (DOCX 111 kb)

Additional file 3: Table S3. Quality ratings for included systematic reviews of melatonin for health. (DOCX 254 kb)

Additional file 4: Table S4. List of randomised trials covered in the systematic reviews. (DOCX 58 kb)

Additional file 5: Table S5. Summary of the adverse effects of MLT reported in the studies included (*N* = 11). (DOCX 24 kb)

## Appendix

The search strategy for MEDLINE (via Ovid)

1. Melatonin.mp.
2. exp Melatonin/
3. urinary 6-sulfatoxymelatonin.mp.
4. 1-3/or
5. Health.mp.
6. exp Health/
7. 5-6/or
8. Review.ti,ab.
9. 4 AND 7 AND 8

## Abbreviations

AE: Adverse effect
CI: confidence interval
DNA: Deoxyribonucleic acid
LED: Light-emitting diode
MA: Meta-analysis
MD: Mean difference
MLT: Melatonin
NR: Narrative review
OR: Odds ratio
PI: Prediction interval
PRISMA: Preferred Reporting Items for Systematic Reviews and Meta-Analyses
RCT: Randomised controlled trial
RR: Risk ratio
SD: Standard deviation
SMD: Standardised mean difference
SR: Systematic review
WMD: Weighted mean difference
