# Supplementary material for: Melatonin and health: an umbrella review of health outcomes and biological mechanisms of action
Source: BMC Med. 2018 Feb 5;16:18. doi: 10.1186/s12916-017-1000-8 (PMC5798185; doi:10.1186/s12916-017-1000-8)
Supplement: Supplementary file 1 — Summary of studies on the effects of exogenous melatonin on health outcomes (N = 120). (DOCX 174 kb) [file 12916_2017_1000_MOESM1_ESM.docx]

**Additional file 1: Table S1. Summary of studies of the effects of exogenous MLT on health outcomes (N=120)**

| **Study (year) [Reference]** | **Number of primary studies/total N** | **QR/QPS** | **Subjects/condition/indication** | **Administration of melatonin (dose, route, frequency, and duration)** | **MAs (y/n)** | **Effects*/health outcomes/ overall result** | **Confounders** | **Comment** |
| --- | --- | --- | --- | --- | --- | --- | --- | --- |
| Andersen (2014) [[1](#_ENREF_1)] | 24/1794 | 8/Low | Peri-operative care | Range: 3 mg – 10 mg (orally, sublingually, intravenously) | Yes | Reduced anxiety SMD=0.88 (95% CI 0.44–1.33); and post-operative pain SMD=1.06 (95% CI 0.23–1.88) | General and regional anaesthesia | Significant heterogeneity was detected *I*^2^=87% for anxiety and 94% for pain |
| Anderson (2012) [[2](#_ENREF_2)] | Inestimable | -3/Not evaluated | Schizophrenia | Range: 2 mg – 10 mg/day | No | Reduction of tardive dyskinesia at 10 mg dose (and not 2 mg) | Vitamin D | EX and EN MLT; mainly animal models, limited data in humans |
| Armour (2004) [[3](#_ENREF_3)] | 10/292 | -7/Not evaluated | Children and adolescents with insomnia | Range: 0.5-25 mg | No | Short term treatment of sleep onset insomnia. | n/a | Five placebo-controlled RCTs and 5 case studies |
| Arora (2016) [[4](#_ENREF_4)] | 8/116 | 8/Moderate | Oral-implantology | Range: 1.2 mg – 50 mg/kg/day | No | Positive effect of MLT on bone formation around implants | n/a | Animal models only. 37.5% of  studies mentioned the translational  value of a study for humans |
| Bellon (2006) [[5](#_ENREF_5)] | 20/598 | 1/Not evaluated | Insomnia | Range: 2.5 mg to 100 mg | No | Shortened sleep onset, improved sleep quality, increased sleep time | Caffeine, alcohol intake, hypnotics use, or dementia | Heterogeneity of populations; subjective and objective outcome measures; MLT agonist ramelteon was evaluated too |
| Bendz (2010) [[6](#_ENREF_6)] | 5/251## | -5/Not evaluated | Paediatric patients with ADHD | Range: 3 to 6 mg | No | Improved sleep onset, sleep duration and sleep latency. | Caffeine and naps | Safety studies, long-term follow-up studies,  retrospective analyses, meta-analyses, review articles, and letters |
| Biran (2014) [[7](#_ENREF_7)] | 6/185& | -9/Not evaluated | Neuroprotection in preterm infants | 10 mg/kg orally or infusion | No | Neuro-prevention against brain lesions; reduced complications and mortality | n/a | Open comparative studies, RCTs; animal models |
| Bonnefont-Rousselot (2010) [[8](#_ENREF_8)] | Inestimable | -9/Not evaluated | Healthy individuals and various clinical conditions | Range: 0.1 mg – 300 mg (orally) | No | Preventing cell damage in acute and chronic conditions | MLT dietary intake | EX and EN MLT; for the prevention and treatment |
| Braam (2009) [[9](#_ENREF_9)] | 9/183 | 6/High | Intellectual disabilities | Range: 0.5 mg–9 mg (up to 4 weeks) | Yes | MD=-33.8, (95% CI 42.97-24.70) for sleep latency; MD=0.83, (95% CI 0.57-1.08) for total sleep time; MD=-0.16 (95% CI -0.30-0.02) for number of wakes per night | n/a | Carry-over effects due to short wash-out period |
| Brigo (2016) [[10](#_ENREF_10)] | 4/102 | 9/Low | Epilepsy | Range: 3 mg - 10 mg | No | No effect on seizure frequency; or quality of life. | n/a | One RCT used 5mg vs 10mg of MLT; 3 RCTs used placebo-controlled design |
| Brzezinski (1998) [[11](#_ENREF_11)] | Inestimable | -9/Not evaluated | Postmenopausal women | Range: 20 - 40 mg | No | Protection against aging and degenerative and neoplastic diseases | Aging, estrogen deprivation, vasomotor symptoms, depression, anxiety, sleep apnea syndrome, obesity | EX and EN MLT; in vitro, animal and human studies; RCTs |
| Brzezinski (2005) [[12](#_ENREF_12)] | 17/284 | 7/Not evaluated | Sleep disorders | Range: 0.3 mg – 10 mg | Yes | ES =4.0 (95% CI 2.5-5.4) for sleep onset latency; 2.2% (95% CI 0.2, 4.2) for sleep efficiency; 12.8 (95% CI 2.9, 22.8) total sleep duration | n/a | 15 out of the 17 studies were of healthy participants (only with insomnia); Cochrane Q was significant for two analyses |
| Bubenik (1998) [[13](#_ENREF_13)] | Inestimable | -9/Not evaluated | Healthy individuals and various clinical conditions | Range: 0.1 mg – 300 mg | No | Multiple health effects | MLT in conjunction with antineoplastic drugs | EX and EN MLT; in vitro, animal and human studies |
| Buscemi (2005) [[14](#_ENREF_14)] | 14/279 | 9/Moderate to high | Sleep disorders | Range: <1 mg to 5 mg | Yes | WMD=-11.7 (95% CI -18.2 to -5.2) for sleep onset latency | Quality of MLT formulations, age, dosage, timing, treatment duration, and primary diagnosis | Substantial heterogeneity among the studies (*I*^2^=81.6%); sleep onset latency was decreased to a greater extent in people with delayed sleep phase syndrome |
| Buscemi (2006) [[15](#_ENREF_15)] | 9/279 | 9/High | Secondary sleep  disorders and sleep disorders accompanying sleep restriction | Range: <1 mg-20mg | Yes | MD=-13.22 (95% CI -27.33 to 0.89) for sleep onset latency in secondary sleep disorders; MD= -0.97 (95% CI -2.26 to 0.33) in sleep disorders accompanying sleep restriction | Duration, content, quality, and formulation of the MLT | *I*^2^=79.2% for sleep onset latency in people with secondary sleep disorders |
| Cardinali (2015) [[16](#_ENREF_16)] | 19/ Inestimable | -5/Not evaluated | Insomnia patients abusing benzodiazepines | Range: 1 mg -24 mg | No | Melatonin promotes sleep by amplifying day/night differences in alertness and sleep quality while displaying a modest sleep inducing effect | n/a | Post-marketing surveillance, retrospective, or open-label study, case report, RCTs of various designs |
| Carlomagno (2011) [[17](#_ENREF_17)] | Inestimable | -9/Not evaluated | Male and female reproductive functions | ≤200 mg/kg daily | No | Beneficial effects on gametogenesis and embryo development | Diet/nutrition | Animal models; in vitro |
| Carpentieri (2012) [[18](#_ENREF_18)] | Inestimable | -9/Not evaluated | Various clinical conditions | Range: 2 mg-50 mg | No | Sleep and immune enhancer, antineoplastic, neuroprotective, antioxidant | MLT in conjunction with various drugs | EX and EN MLT |
| Carrillo-Vico (2005) [[19](#_ENREF_19)] | Inestimable | -9/Not evaluated | Infectious, inflammatory and autoimmune diseases, cancer | Range: 10^-12^ to 10^-6^ M | No | Conflicting evidence regarding inflammation in RA, antitumor efficiency; better well-being of cancer patients | Concomitant administration with IL-2 | In vitro, animal and human studies |
| Cervantes (2008) [[20](#_ENREF_20)] | 11/ Inestimable | -9/Not evaluated | Traumatic CNS injury | Range: 1.5 to 50 mg/kg | No | Improved  morphologic parameters; reduced neurologic deficits and behavioural and cognitive alterations | n/a | Positive impact on neural repair |
| Chaplin (2008) [[21](#_ENREF_21)] | 2/524 | -9/Not evaluated | Elderly insomniacs | 2 mg for 3 weeks | No | OR= 1.97 (95% CI 1.14-3.41) for quality of sleep and daytime function. | n/a | Placebo controlled RCTs |
| Cutando (2011) [[22](#_ENREF_22)] | Inestimable | -6/Not evaluated | Oral cancers | n.m. | No | Growing importance  of MLT in the prognosis and treatment of oral cavity tumours | Lifestyle factors | In vitro and in vivo studies |
| Cutando (2014) [[23](#_ENREF_23)] | Inestimable | -9/Not evaluated | Oral cancers | n.m. | No | Potential oncostatic activity | n/a | The evidence characterised as preliminary |
| De Crescenzo (2017) [[24](#_ENREF_24)] | 8/289 | 8/Moderate | Mood disorders | Range: 0.1–1200 mg | Yes | No effects on mood symptoms | Feeding status, caffeine, smoking, age | *I*^2^=43%, limited quality and quantity of primary studies |
| De Jonghe (2010) [[25](#_ENREF_25)] | 9/330 | 1/Not evaluated | Patients with dementia and sleep disturbances | Range: 3 mg – 9 mg (for a range: 10 days to 35 months) | No | Effectiveness on agitated behaviour; inconclusive results for sleep quality and daytime functioning | n/a | Five case series; 4 RCTs |
| De Rooij (2013) [[26](#_ENREF_26)] | 2/445 | -9/Not evaluated | Patients with delirium | 0.5 mg for 2 weeks | No | Reduction in the incidence of delirium (OR= 0.19 (95%CI 0.06-0.62) | Dementia and co-morbidities | RCTs |
| Dziegiel (2008) [[27](#_ENREF_27)] | Inestimable | -9/Not evaluated | Cancers | Range: 25 μg to 1 mg/kg (daily injections) or 20 mg/day orally | No | Cardio-, nephro-, and myelo-protective actions | Cytostatic drugs | In vitro and in vivo studies |
| Elmahallawy (2015) [[28](#_ENREF_28)] | Inestimable | -9/Not evaluated | Parasitic, viral and bacterial infections | Range: 5 mg/kg orally - 15 mg/kg | No | Potential role for the prevention and treatment of infections | n/a | In vitro and in vivo studies |
| Erdemli (2016) [[29](#_ENREF_29)] | Inestimable | -7/Not evaluated | Cancer, neurodegenerative and ischemic conditions | Range: 1 mg –10 mg/day orally | No | Improved mitochondrial function; prevention again metal toxicity | n/a | Animal models |
| Escames (2006) [[30](#_ENREF_30)] | 1/20 | -9/Not evaluated | Septic shock | 20 mg orally | No | MLT significantly improves survival in sepsis | Conventional therapy + MLT | Animal and human models, EX MLT |
| Escames (2012) [[31](#_ENREF_31)] | Inestimable | -9/Not evaluated | Healthy adults | Range: 1 mg - 80 mg | No | Improved physical performance; increased cardiac output and vagal tone, decreased heart rate and blood pressure | Exercise, light, feeding time | Animal and human models; mechanisms speculative |
| Favero (2014) [[32](#_ENREF_32)] | 1/30 | -9/Not evaluated | Metabolic syndrome, atherosclerosis | 5 mg daily for 2 months | No | Significant improvements in LDL and SBP compared with baseline | n/a | Open-label study; no blinding or control for placebo effects |
| Ferracioli-Oda (2013) [[33](#_ENREF_33)] | 19/ 1683 | 5/Not evaluated | Sleep disorders | Range: 0.1 mg -5 mg | Yes | WMD = 7.06 (95% CI 4.37 to 9.75) for reducing sleep latency; WMD = 8.25 (95% CI 1.74 to 14.75) for total sleep time | Higher MLT doses and longer duration of trials | Objective and subjective measures; inconsistency (heterogeneity) detected |
| Fernando (2014) [[34](#_ENREF_34)] | 9/977 | -8/Not evaluated | Infertility | 3 mg | No | MLT shows promise as adjunct in the treatment of infertility | Myoinositol po + folic acid | Uncontrolled before – after, prospective cohort; single and double-blinded RCTs |
| Fildes (2009) [[35](#_ENREF_35)] | Inestimable | -9/Not evaluated | Organ transplantation | Range: 20 mg/kg to 200 mg/kg | No | Both beneficial or detrimental effects in organ rejection detected | n/a | In vitro and in vivo studies |
| Giannoulia-Karantana (2006) [[36](#_ENREF_36)] | Inestimable | -9/Not evaluated | Various cancers | 20 mg/day (orally) | No | Immunomodulatory, anti-oxidative  and anti-inflammatory properties; increased survival rate | Chemotherapy | In vitro, animal and human studies |
| Golombek (2015) [[37](#_ENREF_37)] | Inestimable | -9/Not evaluated | Insomnia, mild cognitive impairment | Range: 1 mg-24mg (Up to 60 months) | No | Reduction of chronic benzodiazepine use; improvement of cognitive and emotional performance and daily sleep patterns | Benzodiazepines | Double-blind, placebo controlled RCT, retrospective study, open label study, post-marketing surveillance |
| Gomez-Moreno (2010) [[38](#_ENREF_38)] | Inestimable | -6/Not evaluated | Diseases of the oral cavity | 2.5 mg (for herpes) | No | Beneficial effects in periodontal diseases, herpes viral infections and Candida, local inflammatory rocesses, xerostomia, oral ulcers and oral cancer | Magnesium, phosphate, fatty acids and proteins | Animal and human studies |
| Govender (2014) [[39](#_ENREF_39)] | Inestimable | -9/Not evaluated | Various cancers | n.m. | No | Mitochondrial protection; reduced cardiotoxicity | Chemotherapy | In vitro and in vivo studies; both EX and EN MLT |
| Guénole (2011) [[40](#_ENREF_40)] | 12/205 | -3/Not evaluated | Autism spectrum disorders | Range: 2 mg – 9 mg (for up to several years) | No | Improved quality and quantity of sleep; 6 patients had side-effects | Individual chronotypes | Case reports, retrospective studies, open-label, placebo-controlled  RCTs |
| Hansen (2014) [[41](#_ENREF_41)] | 14/634 | 8/Moderate | Depression | Range: 0.5–6 mg daily | Yes | MD=0.97, (95% CI 0.84 to 2.78) for HAD; -1.09, (95% CI 2.60 to 0.42) for BDI. | Sessional variations in mood | Both cross-over and parallel trials were included |
| Hansen (2015) [[42](#_ENREF_42)] | 12/774 | 9/High | Preoperative and postoperative anxiety in adults | Range: 3 mg -14 mg (tablets or sublingually) | Yes | RE=-1.18, (95% CI -2.59 to 0.23) for preoperative anxiety; RE= -5.31, (95% CI -8.78 to -1.84) for post-operative anxiety | Time of administration before surgery (range 50-100 min); baseline anxiety | *I*^2^=54% for main analysis |
| Hardeland (2015) [[43](#_ENREF_43)] | Inestimable | -9/Not evaluated | Brain inflammaging | 1 mg/kg/day | No | Attenuation of inflammatory responses and progression of inflammaging in the brain | n/a | In vitro and in vivo studies |
| Harrod (2005) [[44](#_ENREF_44)] | Inestimable | -9/Not evaluated | Menopause | Unknown | No | Reduced risk of cerebrovascular disease | Estrogen levels | In vitro, animal and human studies |
| Hartley (2014) [[45](#_ENREF_45)] | 12/4973 | -7/Not evaluated | Depression, insomnia | Range: 1 mg – 50 mg (for up to 1 year)# | No | No effects of MLT alone in major depression;  improved sleep outcomes for MLT agonists | n/a | Open label, and placebo-controlled RCTs |
| Heiligenstein (1998) [[46](#_ENREF_46)] | 5/ Inestimable | -9/Not evaluated | Heathy volunteers, insomniacs | Range: 0.3 mg – 5 mg | No | Improved sleep time, sleep efficiency, non-REM sleep; decreased sleep latency | n/a | Double-blind, placebo-controlled RCT |
| Herxheimer (2002) [[47](#_ENREF_47)] | 10/944 | 5/High | Jet-lag | Range: 0.5 mg – 5 mg | No | Effectiveness in preventing or reducing jet-lag | Zolpidem; both eastward and westward flights | No formal meta-analysis |
| Hill (2015) [[48](#_ENREF_48)] | Inestimable | -9/Not evaluated | Breast cancers | n.m. | No | Suppression of cancer growth | ALAN | In vitro, animal and human studies |
| Hong (2010) [[49](#_ENREF_49)] | Inestimable | -7/Not evaluated | Spinal cord injury | Range: 10 mg/kg- 50 mg/kg | No | Faster functional recovery after spinal cord injury | Dexamethasone (0.025 mg/kg); exercise | Animal studies; hypothetical evidence in humans |
| Huang (2014) [[50](#_ENREF_50)] | 6/1871 | 9/high | Major depression | Range: 25 mg – 50 mg (agomelatine) | Yes | RR= 1.08 (95% CI 1.02–1.15) for response rate; RR= 1.12 (95% CI 1.01–1.24) for acute remission rate | Number of episodes, duration of current episode | Significantly fewer dropouts owing to AEs from agomelatine (RR=0.38, 95% CI 0.25–0.57) |
| Jan (2007) [[51](#_ENREF_51)] | Inestimable | -9/Not evaluated | Pediatric sleep disorders | Range: 1 mg – 10 mg | No | Effectiveness in chronic sleep difficulties | Brain damage | Animal and human  studies |
| Jansen (2006) [[52](#_ENREF_52)] | 5/334 | 9/high | Dementia | Range: 2.5 mg – 10 mg (for up to 2 years) | Yes | WMD=0.29, (95% CI - 0.63, 1.22) for cognition; WMD= -3.48, (95% CI - 4.89, - 2.07) for behaviour and mood | n/a | 4 (out of 5) RCTs were of high quality |
| Jena (2014) [[53](#_ENREF_53)] | Inestimable | -9/Not evaluated | Ulcerative colitis | Capsules (unspecified dose) | No | Improved and worsened symptoms of ulcerative colitis | Crohn’s disease, jetlag | Case reports in humans; chemically  induced disease in animals |
| Jung (2006) [[54](#_ENREF_54)] | Inestimable/539 | -9/Not evaluated | Various cancers | Range: 5-700 mg/m^2^/d topically for 4 weeks | No | Increased survival rate; decreased side-effects of chemotherapy | Chemotherapy | In vitro and in vivo studies; MLT used for cancer prevention |
| Karaaslan (2015) [[55](#_ENREF_55)] | Inestimable | -9/Not evaluated | Aging, cancer,  atherosclerosis, neurodegenerative, inflammatory disorders, and diabetes | Range: 5 mg/kg to 10 mg daily/30 days | No | Antioxidant properties were found in various non-communicable diseases | n/a | Both EX and EN MLT; in vitro, animal and human models |
| Keegan (2014) [[56](#_ENREF_56)] | 7/17 | 0/Low | Acquired brain injury | Range: 2.5 mg – 25 mg | No | Promising evidence for MLT in sleep impairment | n/a | Double-blind  cross-over RCT, case studies |
| Kennaway (2015) [[57](#_ENREF_57)] | Inestimable | -9/Not evaluated | The safety of MLT in paediatrics | Range: 0.7 μg/kg–185 μg/kg (injections) | No | Insufficient evidence to support safety in children | n/a | Animal and human studies |
| Kuriyama (2014) [[58](#_ENREF_58)] | 13/ 5812 | 9/Moderate | Insomnia | Range: 4 mg - 32 mg | Yes | WMD= -4.30 (95% CI, -7.01 to -1.58) for subjective sleep latency; SMD=-0.074 (95% CI, -0.13 to -0.02) for improved sleep quality. | Both primary and chronic insomniacs; bipolar and sleep apnea patients | Parallel an crossover trials; *I^2^*=53.5% for subjective sleep  latency |
| Leger (2015) [[59](#_ENREF_59)] | 6/571 | -9/Not evaluated | Chronic insomnia | Tasimelteon  Range: 1 mg - 50 mg for up to 2 years | No | AEs included headache, diarrhoea, dry mouth,  alanine aminotransferase increased, somnolence, dizziness and nightmare/  abnormal dreams | n/a | Open-label and double-blind,  placebo controlled,  crossover and parallel RCTs. Both efficacy and  safety were evaluated |
| Lemoine (2012) [[60](#_ENREF_60)] | 4/1374 | -9/Not evaluated | Insomnia in older patients | Circadin®  Orally 2-mg/day for 3 months | No | Clinically relevant improvements in sleep quality and morning alertness | n/a | P Lemoine is  the primary investigator in two Circadin® trials |
| Leone (1998) [[61](#_ENREF_61)] | 1/20 | -9/Not evaluated | Cluster headache | A single dose of oral MLT 10 mg for 2 weeks | No | Significant reduction in the mean number of daily headache attacks | 2 patients continued  preventive treatment | Double-blind, placebo-controlled RCT, case report, animal study |
| Liira (2014) [[62](#_ENREF_62)] | 9/inestimable@ | 9/Low-moderate | Sleepiness and sleep-wake disturbances | Range: 1 mg - 10 mg | Yes | MD=24 min (95% CI 9.8 to 38.9) for sleep length; MD= 0.37 min (95% CI - 1.55 to 2.29) for sleep latency | n/a | No evidence of heterogeneity or publication bias |
| Liu (2012) [[63](#_ENREF_63)] | 8/1122 | 7/High | Chronic insomnia | Range: 4 mg – 32 mg | Yes | MD=-4.22 (95% CI - 5.66 to2.77) for subjective sleep latency. For AEs RR=1.11 (95% CI 1.03 to 1.20) | n/a | MD for fixed-effects model |
| Macleod (2004) [[64](#_ENREF_64)] | 13/432 | 7/Low | Ischaemic stroke in animals | Range: 2.5 mg - 50 mg/kg | Yes | ES=0.428 (95% CI 0.393 to 0.463) for cerebral ischaemia | Ketamine anaesthesia | Neuroprotective properties in human stroke were suggested |
| Maldonado(2007) [[65](#_ENREF_65)] | Inestimable | -5/Not evaluated | Thermal injury (burns) | Range: 10 mg -100 mg (various routes from oral to injections) | No | Reduced morbidity and mortality in patients with thermal injury | n/a | In vitro, animal, human studies |
| Maldonado(2009) [[66](#_ENREF_66)] | Inestimable | -9/Not evaluated | Psychiatric disorders | Range: 3–10 mg | No | Useful in the co-treatment of bipolar disorders, depression, and schizophrenia | n/a | MOA via reduction of neuroinflammation |
| Maria (2014) [[67](#_ENREF_67)] | Inestimable | -9/Not evaluated | Osteopenia, osteoporosis, and periodontal disease | Range: 3 mg – 50 mg/kg | No | Reduced risk for osteopenia, osteoporosis, and bone fracture; positive effect on overall bone health | n/a | Multicentre RCTs, animal and in vitro studies |
| Marseglia (2014) [[68](#_ENREF_68)] | Inestimable | -9/Not evaluated | Atopic dermatitis and asthma | 3 mg for 28 days | No | Inhibition of development of atopic eczema; regulation of smooth muscle tone in asthma; not recommended in bronchial asthma | n/a | Data judged as incomplete. One study in adults with atopic eczema (plasma MLT levels) |
| Mehta (2014) [[69](#_ENREF_69)] | Inestimable | -7/Not evaluated | Oral cancers | n.m. | No | Promising results regarding the role of melatonin as anti-carcinogenic agent | n/a | In vitro and in vivo studies; both EX and EN MLT |
| McGrane (2015) [[70](#_ENREF_70)] | 6/116 | -2/Not evaluated | REM sleep behavior disorder | Range: 3 mg–12 mg | No | Reductions in clinical behavioural outcomes and decrease in muscle tonicity during REM sleep | Clonazepam | Case series, RCTs |
| Mihara (2015) [[71](#_ENREF_71)] | 4/358 | 9/Low | Prevention of agitation in children recovering from general anaesthesia | Range: 0.05 to  0.5mg/kg ^-1^ | Yes | RR=0.31, (95%CI 0.16- 0.60) for MLT versus placebo for prevention of agitation | Surgery, anaesthesia type, age, control group medication | Trial sequential analysis  corrected the 95% CI to 0.07-1.47;  MLT dose was correlated with the effect (P = 0.024) |
| Mills (2005) [[72](#_ENREF_72)] | 10/643 | 1/Low | Cancer | Range: 10 mg – 40 mg mg/d (oral) | Yes | RR= 0.66 (95% CI: 0.59–0.73) for the risk of death at 1 year. | n/a | Eggers test for asymmetry= 1.260 (approx.. 95% CI - 2.508 to -0.011) |
| Miroddi (2015) [[73](#_ENREF_73)] | 5/356 | 3/Low | Tinnitus | 3 mg/day | No | Improved tinnitus and sleep  disturbances | Sulpiride and sulodexide | Median Jadad score = 2; majority judged as High or Unclear risk of bias |
| Ma (2016) [[74](#_ENREF_74)] | 5/1186 | -9/Not evaluated | NSCLC | 20 mg/day | No | Improved survival rate; tumour regression | MLT used in conjunction with usual care for NSCLC | Animal models and human trials |
| Malhotra (2004) [[75](#_ENREF_75)] | Inestimable | -9/Not evaluated | Cancer, sleep disorders, mental health, cardio-metabolic, gastric, excretory functions | Range: 0.5 mg-50 mg | No | Improved immunity; reduced oxidative stress | Lifestyle; environmental factors | Experimental studies and human trials |
| Mozaffari (2010) [[76](#_ENREF_76)] | 8/92 | -3/Moderate | IBS | 3 mg orally for median of 8 weeks | No | Decreased abdominal pain and improved overall IBS symptom scores | n/a | All studies were RCTs; median Jadad score= 3. |
| Nduhirabandi (2012) [[77](#_ENREF_77)] | Inestimable | -9/Not evaluated | Obesity-related metabolic disorders | Range: 1 mg to 300 mg per day | No | Prevention or reduction of obesity and its related metabolic disorders | n/a | In vitro, animal and human studies; both EX and EN MLT; evidence judged as preliminary |
| No authors listed (2015) [[78](#_ENREF_78)] | 9/Inestimable | -9/Not evaluated | Children with neurodevelopmental disorders | Range: 0.5mg-12mg | No | Reduce the delay before sleep onset and increase the total duration of sleep | Other drugs affecting the CYP enzymes | The cost of a 30 tablet pack of 2mg of Circadin® is £15.39 |
| Nowak (1998) [[79](#_ENREF_79)] | Inestimable | -9/Not evaluated | Sleep disorders, neoplastic diseases | Range :1 mg - 50 mg/day (orally) | No | Beneficial in the treatment of sleep disturbances; prolonged survival and reduced the number and severity of complications in cancer patients | ALAN, circadian phase shifts, age | Data analysed and synthesised narratively |
| Olde Rikkert (2001) [[80](#_ENREF_80)] | 12/95 | -3/Not evaluated | Elderly insomniacs | Range: 0.5 mg – 6 mg | No | Three studies showed effectiveness of MLT in sleep efficiency; and three showed no effect | Benzodiazepines | Retrospective case study, open label and double blind cross-over RCTs |
| Panzer (1997) [[81](#_ENREF_81)] | 15/634 | -9/Not evaluated | Various cancers | 3200 mg/kg in rats; in humans 250 mg (orally) 6 hourly for 25-30 days | No | Antineoplastic effects | MLT used in conjunction with usual cancer care | In vitro, animal and human studies |
| Romero (2014) [[82](#_ENREF_82)] | Inestimable | -9/Not evaluated | Prevention against metal toxicity | Range: 5 mg/kg/day to 25 mg/kg/day | No | Reduction of metal-induced toxicity; chelation | Adriamycin, alpha-lipoic  acid, vitamin C | Animal and in vitro models |
| Rossignol (2011) [[83](#_ENREF_83)] | 5/61** | 8/Moderate | Autism spectrum  disorders | Range: 0.75 mg – 15 mg | Yes | Hedge’s g=1.97 (95% CI 1.10–2.84) for sleep duration | n/a | Small total sample; different washout periods |
| Ryung Wang (2016) [[84](#_ENREF_84)] | 4/138 | 7/Low | Patients on atypical antipsychotics | Range: 3 mg- 8 mg/day | No | Lowered blood pressure among bipolar disorder patients; but not in schizophrenics; improved lipid profiles and BMI and attenuated weight gain | Antipsychotic drugs | MLT agonist – ramelteon was used in one study |
| Sajith (2007) [[85](#_ENREF_85)] | 11/174 | -3/Not evaluated | Children and adolescents intellectual disabilities | Range: 0.5 mg -10 mg | No | Reduced sleep onset  latency; improved total sleep time | n/a | Double-blind placebo-controlled RCTs, open/observational studies |
| Sanchez-Barcelo (2005) [[86](#_ENREF_86)] | Inestimable | -9/Not evaluated | Breast cancer | Unknown | No | Prevention and treatment of mammary cancer | n/a | In vitro, animal and human studies |
| Sanchez-Barcelo (2010) [[87](#_ENREF_87)] | 20/2186 | -9/Not evaluated | Ocular, blood, neurological, infectious, GI diseases, CVD, DM, RA, FM, CFS, sleep disturbances, aging and depression | Range: 0.1 - 300 mg orally | No | Effectiveness in macular degeneration, glaucoma, gastric mucosa, IBS, HTN, DM, side effects of cancer treatment; hemodialysis, sleep disorders; neurological degenerative diseases | Various concomitant treatments | No critical appraisal of the evidence |
| Sanchez-Barcelo (2012) [[88](#_ENREF_88)] | Inestimable | -9/Not evaluated | Breast cancer | Range: 5 mg-21mg/day | No | Prevention of mammary cancer; reduction of the side effects of chemotherapy and radiation | Lifestyle factors, obesity | In vitro, animal and human studies |
| Seely (2012) [[89](#_ENREF_89)] | 19/3697 | 9/Low | Various cancers | Single oral doses of MLT in the evening  (range: 10-40 mg, mode=20 mg in 16 studies) | Yes | RR=0.63 (95% CI 0.53 -0.74) for 1-year mortality; RR= 2.33 (95% CI = 1.29-4.20), RR=1.90 (1.43-2.51), RR= 1.51 (1.08-2.12) for complete response, partial response, and stable disease respectively | MLT used in conjunction with chemotherapy, radiotherapy, supportive care, and palliative  care | No serious side effects related to MLT were found |
| Seko (2014) [[90](#_ENREF_90)] | 5/680 | 9/Low | Infertility | 3mg/day | Yes | RR= 1.21, (95% CI, 0.98–1.50) for pregnancy rate; MD= 0.6, (95% CI, -0.2–2.2) for oocytes retrieved | Myoinositol and folic acid | Substantial heterogeneity (I² = 69%) for oocytes |
| Shirazi (2007) [[91](#_ENREF_91)] | Inestimable | -9/Not evaluated | Radioprotection | Range: 1 mg – 800 mg | No | Radio-protective and anticancer effects | Radiotherapy | Both healthy volunteers and diseased patients; EX and EN MLT; in vitro, and in vivo studies |
| Singh (2014) [[92](#_ENREF_92)] | Inestimable | -9/Not evaluated | Depression, insomnia, epilepsy, AD, DM, obesity, alopecia, migraine, cancer, and immune and cardiac disorders | Range: 20 mg – 800 mg | No | Improved health outcomes in various chronic diseases | n/a | Animal and human models |
| Srinivasan (2009) [[93](#_ENREF_93)] | Inestimable | -9/Not evaluated | Reproductive functions | 3 mg (1 study) | No | Limited effects of MLT on human reproductive processes such as ovulation and fertility | Neuropeptides, neurotransmitters, and neurosteroids in the HPA axis | In vitro, animal and clinical studies of EX and EN MLT; evidence judged as inconclusive |
| Srinivasan (2012) [[94](#_ENREF_94)] | 56/ Inestimable | -9/Not evaluated | Various pain syndromes | Range: from 3 mg orally for 4 weeks 30 minutes before bed time to 25 mg/day for the duration of six months | No | Reduced pain intensity; antinociceptive and analgesic effects | Concomitant medication use, e.g., fluoxetine, St John Wort | Animal and clinical studies |
| Srinivasan (2012) [[95](#_ENREF_95)] | 4/157 | -9/Not evaluated | Septic conditions in infants or adults | Range: 10mg/day (orally) to 10x10mg/kg (intravenously) | No | Protective action against sepsis | n/a | No critical appraisal; 3 (out of 4) studies done by the same group |
| Sun (2016) [[96](#_ENREF_96)] | Inestimable | -9/Not evaluated | Cardiovascular diseases | Range: 2–5 mg/day for 7–90 days to 10 mg/day for 14 months | No | Effects on ischemia-reperfusion injury, myocardial chronic intermittent hypoxia injury, pulmonary hypertension, hypertension, valvular heart diseases, vascular  diseases, and lipid metabolism | n/a | In vitro, animal and clinical studies; no critical appraisal |
| Tamura (2008) [[97](#_ENREF_97)] | Inestimable | -9/Not evaluated | Reproductive functions (pregnancy) | Range: 1-100 mg/kg | No | Reduced pregnancy-related complications - abortion, pre-eclampsia and fetal brain damage | Vitamin A,C, E in three studies | Animal and human studies |
| Tamura (2009) [[98](#_ENREF_98)] | 9/ Inestimable | -9/Not evaluated | Reproductive functions | Range: 2- 30 mg/kg (various routes from oral to injections) | No | Improved ovarian physiology - follicular development, ovulation, oocyte maturation (quality), and luteal function | Vitamin E (600 mg/day) in one of the studies | In vitro, animal, human studies |
| Tamura (2014) [[99](#_ENREF_99)] | 9/ Inestimable | -9/Not evaluated | Reproductive functions | Range: 1-6 mg | No | Increased oocyte maturation, embryo development and luteinisation  of granulosa cells;  fertilization and pregnancy rates | n/a | Animal, human studies |
| Terry (2009) [[100](#_ENREF_100)] | 3/45 | -9/Not evaluated | IBS | 3 mg/d (range: 2-8 weeks) | No | Decreased mean abdominal pain; improved IBS score, and QOL | One study’s patients had sleep  disturbances | 13 animal, 3 human studies; quality of trials Not evaluated |
| Tordjman (2013) [[101](#_ENREF_101)] | 19/757^^ | -9/Not evaluated | Autism spectrum  disorders | Range: 0.5 mg -  12 mg | No | Increased total sleep time and reduced sleep onset latency; improved behavioural and social outcomes | CBT | Case studies, case series, open label, RCTs, reviews |
| Turk (2003) [[102](#_ENREF_102)] | Inestimable | -9/Not evaluated | Sleep disorders | 0.5 mg/nightly | No | Sleep inducer | n/a | RCTs, case reports |
| Van Geijlswijk (2010) [[103](#_ENREF_103)] | 9/317 | 6/high | Delayed sleep phase disorder | Range: 0.3 mg – 6 mg | Yes | MD=-1.18 (95% CI -1.48 to -0.89) for dim light MLT onset; MD=0.67 hours (95% CI: 0.45-0.89) for clock hour of sleep onset; MD=23.27 min (95% CI: 4.83 -41.72) for decreased sleep-onset latency; MD= -0.28 (95% CI -0.66 to 0.09) for wake-up time | Age | Sleep diaries and with actigraphy measures were used |
| Vielma (2014) [[104](#_ENREF_104)] | Inestimable | -9/Not evaluated | Resistance against infections | Range: 5 mg/kg to 100 mg/kg | No | MLT has the potential to fight bacterial, viral, and parasitic infections | n/a | In vitro, and in vivo studies |
| Vijayalaxmi (2003) [[105](#_ENREF_105)] | Inestimable | -9/Not evaluated | Various cancers | Range: 1 mg to 1 g for 30 days | No | Favorable efficacy to toxicity ratio in the treatment of human cancers | n/a | In vitro, and in vivo studies of both healthy individuals (toxicity) and cancer patients |
| Vijayalaxmi (2004) [[106](#_ENREF_106)] | Inestimable | -9/Not evaluated | Radioprotection in cancer | Unknown | No | Radio-protective and anticancer effects via free radical scavenging | Radiotherapy | EX and EN MLT; in vitro, and in vivo studies |
| Vural (2014) [[107](#_ENREF_107)] | 16/506 | 1/Variable | Older adults with various conditions | Range: 0.1 mg to 50 mg/  kg orally (for up to for 6 months) | No | Enhanced sleep onset, efficiency, and quality | n/a | 9 RCTs, 2 open-label studies and 5 case series |
| Wade (2008) [[108](#_ENREF_108)] | 2/543 | -7/ Not evaluated | Elderly insomniacs | 2 mg orally | No | Significantly improvements in morning alertness and quality of sleep | n/a | Placebo-controlled RCTs; conflict of interest declared by the review authors |
| Wang-Weigand (2009) [[109](#_ENREF_109)] | 4/1122 | -8/ Not evaluated | Insomniacs | Ramelteon 8 mg | No | MD= –13.1 min (p <0.001) | n/a | ANCOVA used |
| Wang (2012) [[110](#_ENREF_110)] | 8/761 | 8/Moderate | Cancer | 20 mg/day orally | Yes | RR = 1.95, 95% CI, 1.49–2.54 for partial remission; RR = 1.90; 95% CI, 1.28–2.83 for survival rate | MLT used as adjunct | 6 (out of 8) eligible RCTs were done by the same group |
| Wang (2016) [[111](#_ENREF_111)] | 4/138 | 8/Moderate | Psychiatric disorders | Range: 3-8mg for median of 8 weeks | No | Beneficial in lowering blood pressure among bipolar patients | Atypical antipsychotics | High clinical heterogeneity; MLT agonist used |
| Wilhelmsen (2011) [[112](#_ENREF_112)] | 7/298 | -9/Not evaluated | Pain syndromes | Range: 2 mg – 5 mg (median=3) | No | Analgesic effects in FM, IBS, migraine | Fluoxetine | Pilot study, open label, RCTs |
| Wilkinson (2016) [[113](#_ENREF_113)] | 1/60 | 9/ Not evaluated | Reproductive functions (pregnancy) | Range: ≤ 10mg - > 70 mg/day | No | Fetal neuroprotection in animals | n/a | The only one trial is currently ongoing |
| Winkler (2014) [[114](#_ENREF_114)] | 3/433@ | 9/Moderate | Primary insomnia | Range: 2 mg -16 mg | No | ES=0.06 (95% CI -0.55 to 0.67, p=0.847) for total sleep time; ES=-0.48 (95% CI -1.10 to 0.13, p= 0.125) for sleep onset latency | n/a | Median Jadad score=3 |
| Witt-Enderby (2006) [[115](#_ENREF_115)] | Inestimable | -9/Not evaluated | Osteoporosis, cancer, sleep disorders | Unknown | No | Prevention of osteoporosis, cancer, sleep enhancer | n/a | EX and EN MLT; human and animal models |
| Wright (2015) [[116](#_ENREF_116)] | 6/322 | 8/Low | Sleep disorders; benzodiazepine withdrawal | Range: 2 mg – 5 mg (up to 18 weeks) | Yes | OR= 0.72, (95% CI 0.21–2.41) for benzodiazepine discontinuation; inconsistent results for sleep quality | Benzodiazepines | *I*^2^=76%; most trials judged as unclear risk of bias |
| Xu (2015) [[117](#_ENREF_117)] | 7/520 | 8/Low to moderate | Sleep disorders and cognition in dementia | Range: 2.5 mg – 10 mg (for up to 24 weeks) | Yes | MD=24.36 min (95% CI: 3.26-45.46) for total sleep time; MD (95% CI:-0.55 to -0.67) for cognitive function | n/a | Heterogeneity (*I*^2^ >50%) across studies |
| Yang (2016) [[118](#_ENREF_118)] | 13/Inestimable | 9/Moderate | Spinal cord injury | Range: 2.5 mg/kg – 100 mg | Yes | MD=1.53 (95% CI 0.07 to 2.99) for neurological  recovery (BBB scale); MD= 0.93; 95% CI 0.30 to 1.56) for motor function scores | n/a | Rat models (median sample size=60); 12.5 mg/kg of MLT was most effective dose |
| Yousaf (2010) [[119](#_ENREF_119)] | 10/788 | 7/Low | Perioperative anxiety and analgesia | Range: 5mg/ single dose to 14.8 mg/kg sublingually and orally | No | MLT “is effective in ameliorating preoperative anxiety in adults, but its analgesic effects remain controversial in the perioperative period” | n/a | Significant clinical heterogeneity |
| Zhang (2016) [[120](#_ENREF_120)] | 9/300 | 4/Not evaluated | Sleep disorders in patients with neurodegenerative disease | Range: 2.5 mg/d – 50 mg/d for up to 24 weeks | Yes | MD= 4.20 (95 % CI 0.92–7.48) for sleep quality | n/a | No effects on objective sleep outcomes in both AD and PD patients |

**Table 5** Footnote: @- for MLT (or MLT agonists) only; * also includes adverse effects; **- pertains to randomised, cross-over, placebo controlled trials only; ^^- number excludes systematic reviews; #- for melatonin agonist; ##- number reflects RCTs only; &- infants treated with MLT only; AD- Alzheimer’s disease; ADHD- attention-deficit/hyperactivity disorder; AEs- adverse effects; ALAN- artificial light at night; ANCOVA- analysis of covariance; BBB- Basso, Beattie, and Bresnahan; BDI- Beck Depression Inventory; CBT-Cognitive Behavioural Therapy ; CFS- chronic fatigue syndrome; CI-confidence interval; CR- controlled release; CVD- cardiovascular diseases; CYP- cytochrome P450; DBP- diastolic blood pressure; DM- diabetes mellitus; ES- effect size; FM- fibromyalgia; GI - gastrointestinal tract; HAD- Hospital Anxiety and Depression Scale; HPA- hypothalamic-pituitary ovarian axis; HTN- hypertension; IBS- Irritable Bowel Syndrome; IL-2- interleukin 2; LDL- low-density lipoprotein; MAs- meta analyses; MD- mean difference; MLT-melatonin; MOA-mechanism of action; NSCLC- Non-small-cell lung cancer; OR- odd ratio; PD-Parkinson’s disease; QOL- quality of life; QPS- quality of primary studies (as evaluated by the authors of primary studies); QR- quality of the reviews (Oxman score, please refer also to additional Table 7); RA- rheumatoid arthritis; RE- relative effect; REM- rapid eye movement; RCT- randomised controlled trial; RR-relative risk; SBP- systolic blood pressure; SMD- standardised mean difference; WMD- weighted mean difference.

**References**

1. Andersen LP, Werner MU, Rosenberg J, Gogenur I: A systematic review of peri-operative melatonin. *Anaesthesia* 2014, 69(10):1163-1171.

2. Anderson G, Maes M: Melatonin: an overlooked factor in schizophrenia and in the inhibition of anti-psychotic side effects. *Metab Brain Dis* 2012, 27(2):113-119.

3. Armour D, Paton C: Melatonin in the treatment of insomnia in children and adolescents. *Psychiatric Bulletin* 2004, 28(6):222-224.

4. Arora H, Ivanovski S: Melatonin as a pro-osteogenic agent in oral implantology: a systematic review of histomorphometric outcomes in animals and quality evaluation using ARRIVE guidelines. *J Periodontal Res*.

5. Bellon AM: Searching for New Options for Treating Insomnia: Are Melatonin and Ramelteon Beneficial? [Article]. *J Psychiatr Pract* 2006, 12(4):229-243.

6. Bendz LM, Scates AC: Melatonin treatment for insomnia in pediatric patients with attention-deficit/hyperactivity disorder. *Ann Pharmacother* 2010, 44(1):185-191.

7. Biran V, Phan Duy A, Decobert F, Bednarek N, Alberti C, Baud O: Is melatonin ready to be used in preterm infants as a neuroprotectant? *Dev Med Child Neurol* 2014, 56(8):717-723.

8. Bonnefont-Rousselot D, Collin F: Melatonin: Action as antioxidant and potential applications in human disease and aging. *Toxicology* 2010, 278(1):55-67.

9. Braam W, Smits MG, Didden R, Korzilius H, Van Geijlswijk IM, Curfs LM: Exogenous melatonin for sleep problems in individuals with intellectual disability: a meta-analysis. *Dev Med Child Neurol* 2009, 51(5):340-349.

10. Brigo F, Igwe SC: Melatonin as add-on treatment for epilepsy. *Cochrane Database Syst Rev* 2016, 2016(3).

11. Brzezinski A: 'Melatonin replacement therapy' for postmenopausal women: Is it justified? *Menopause* 1998, 5(1):60-64.

12. Brzezinski A, Vangel MG, Wurtman RJ, Norrie G, Zhdanova I, Ben-Shushan A, Ford I: Effects of exogenous melatonin on sleep: A meta-analysis. *Sleep Med Rev* 2005, 9(1):41-50.

13. Bubenik GA, Blask DE, Brown GM, Maestroni GJ, Pang SF, Reiter RJ, Viswanathan M, Zisapel N: Prospects of the clinical utilization of melatonin. *Biol Signals Recept* 1998, 7(4):195-219.

14. Buscemi NP, Vandermeer BM, Hooton NB, Pandya RMPH, Tjosvold LM, Hartling LM, Baker GPD, Klassen TPMDM, Vohra SMDM: The Efficacy and Safety of Exogenous Melatonin for Primary Sleep Disorders: A Meta-Analysis. *J Gen Intern Med* 2005, 20(12):1151-1158.

15. Buscemi Nra, Vandermeer Bs, Hooton Npc, Pandya Rpm, Tjosvold Lrl, Hartling Lad, Vohra Sd, Klassen TPd, Baker Gp, chair: Efficacy and safety of exogenous melatonin for secondary sleep disorders and sleep disorders accompanying sleep restriction: meta-analysis. *BMJ* 2006, 332(7538):385-393.

16. Cardinali DP, Golombek DA, Rosenstein RE, Brusco LI, Vigo DE: Assessing the efficacy of melatonin to curtail benzodiazepine/Z drug abuse. *Pharmacol Res* 2015.

17. Carlomagno G, Nordio M, Chiu TT, Unfer V: Contribution of myo-inositol and melatonin to human reproduction. *Eur J Obstet Gynecol Reprod Biol* 2011, 159(2):267-272.

18. Carpentieri A, Diaz de Barboza G, Areco V, Peralta Lopez M, Tolosa de Talamoni N: New perspectives in melatonin uses. *Pharmacol Res* 2012, 65(4):437-444.

19. Carrillo-Vico A, Guerrero JM, Lardone PJ, Reiter RJ: A Review of the Multiple Actions of Melatonin on the Immune System. *Endocrine* 2005, 27(2):189-200.

20. Cervantes M, Morali G, Letechipia-Vallejo G: Melatonin and ischemia-reperfusion injury of the brain. *J Pineal Res* 2008, 45(1):1-7.

21. Chaplin SMM, Nutt DDMFFF: Melatonin (Circadin): a novel hypnotic for use in older patients. *Prescriber* 2008, 19(20):21-24.

22. Cutando A, Aneiros-Fernandez J, Aneiros-Cachaza J, Arias-Santiago S: Melatonin and cancer: current knowledge and its application to oral cavity tumours. *J Oral Pathol Med* 2011, 40(8):593-597.

23. Cutando A, Lopez-Valverde A, J DEV, Gimenez JL, Carcia IA, RG DED: Action of melatonin on squamous cell carcinoma and other tumors of the oral cavity (Review). *Oncol Lett* 2014, 7(4):923-926.

24. De Crescenzo F, Lennox A, Gibson JC, Cordey JH, Stockton S, Cowen PJ, Quested DJ: Melatonin as a treatment for mood disorders: A systematic review. *Acta Psychiatrica Scandinavica* 2017.

25. De Jonghe A, Korevaar JC, Van Munster BC, De Rooij SE: Effectiveness of melatonin treatment on circadian rhythm disturbances in dementia. Are there implications for delirium? A systematic review. *Int J Geriatr Psychiatry* 2010, 25(12):1201-1208.

26. De Rooij SE, Van Munster BC: Melatonin deficiency hypothesis in delirium: A synthesis of current evidence. *Rejuvenation Res* 2013, 16(4):273-278.

27. Dziegiel P, Podhorska-Okolow M, Zabel M: Melatonin: adjuvant therapy of malignant tumors. *Med Sci Monit* 2008, 14(5):Ra64-70.

28. Elmahallawy EK, Luque JO, Aloweidi AS, Gutiérrez-Fernández J, Sampedro-Martínez A, Rodriguez-Granger J, Kaki A, Agil A: Potential relevance of melatonin against some infectious agents: A review and assessment of recent research. *Curr Med Chem* 2015, 22(33):3848-3861.

29. Erdemli HK, Akyol S, Armutcu F, Gulec MA, Canbal M, Akyol O: Melatonin and caffeic acid phenethyl ester in the regulation of mitochondrial function and apoptosis: The basis for future medical approaches. *Life Sci* 2016, 148 Supplement(C):305-312.

30. Escames G, Acuna-Castroviejo D, Lopez LC, Tan D-x, Maldonado MD, Sanchez-Hidalgo M, Leon J, Reiter RJ: Pharmacological utility of melatonin in the treatment of septic shock: experimental and clinical evidence. *J Pharm Pharmacol* 2006, 58(9):1153-1165.

31. Escames G, Ozturk G, Bano-Otalora B, Pozo MJ, Madrid JA, Reiter RJ, Serrano E, Concepcion M, Acuna-Castroviejo D: Exercise and melatonin in humans: reciprocal benefits. *J Pineal Res* 2012, 52(1):1-11.

32. Favero G, Rodella LF, Reiter RJ, Rezzani R: Melatonin and its atheroprotective effects: a review. *Mol Cell Endocrinol* 2014, 382(2):926-937.

33. Ferracioli-Oda E, Qawasmi A, Bloch MH: Meta-analysis: melatonin for the treatment of primary sleep disorders. *PLoS One* 2013, 8(5):e63773.

34. Fernando S, Rombauts L: Melatonin: shedding light on infertility?--A review of the recent literature. *J Ovarian Res* 2014, 7:98.

35. Fildes JE, Yonan N, Keevil BG: Melatonin--a pleiotropic molecule involved in pathophysiological processes following organ transplantation. *Immunology* 2009, 127(4):443-449.

36. Giannoulia-Karantana A, Vlachou A, Polychronopoulou S, Papassotiriou I, Chrousos GP: Melatonin and immunomodulation: connections and potential clinical applications. *Neuroimmunomodulation* 2006, 13(3):133-144.

37. Golombek DAa, Pandi-Perumal SRb, Brown GMc, Cardinali DPd: Some implications of melatonin use in chronopharmacology of insomnia. *Eur J Pharmacol* 2015, 762 Supplement(C):42-48.

38. Gomez-Moreno G, Guardia J, Ferrera MJ, Cutando A, Reiter RJ: Melatonin in diseases of the oral cavity. *Oral Dis* 2010, 16(3):242-247.

39. Govender J, Loos B, Marais E, Engelbrecht AM: Mitochondrial catastrophe during doxorubicin-induced cardiotoxicity: a review of the protective role of melatonin. *J Pineal Res* 2014, 57(4):367-380.

40. Guenole F, Godbout R, Nicolas A, Franco P, Claustrat B, Baleyte JM: Melatonin for disordered sleep in individuals with autism spectrum disorders: systematic review and discussion. *Sleep Med Rev* 2011, 15(6):379-387.

41. Hansen MV, Danielsen AK, Hageman I, Rosenberg J, Gogenur I: The therapeutic or prophylactic effect of exogenous melatonin against depression and depressive symptoms: a systematic review and meta-analysis. *Eur Neuropsychopharmacol* 2014, 24(11):1719-1728.

42. Hansen MV, Halladin NL, Rosenberg J, Gogenur I, Moller MA: Melatonin for pre- and postoperative anxiety in adults. *Cochrane Database Syst Rev* 2015(4).

43. Hardeland Ra, Cardinali DPb, Brown GMc, Pandi-Perumal SRd: Melatonin and brain inflammaging. *Prog Neurobiol* 2015, 127-128 Suppl.(C):46-63.

44. Harrod CG, Bendok BR, Batjer HH: Interactions between melatonin and estrogen may regulate cerebrovascular function in women: Clinical implications for the effective use of HRT during menopause and aging. *Med Hypotheses* 2005, 64(4):725-735.

45. Hartley S, Quera-Salva M-A: Implication of Circadian Rhythms and Melatonin in Major Depressive Disorder: The Evidence Base for New Antidepressant Treatment. *Curr Psychiatry Rev* 2014, 10(3):223-234.

46. Heiligenstein E, Guenther G: Over-the-counter psychotropics: a review of melatonin, St John's wort, valerian, and kava-kava. *J Am Coll Health* 1998, 46(6):271-276.

47. Herxheimer A, Petrie KJ: Melatonin for the prevention and treatment of jet lag. *Cochrane Database Syst Rev* 2002(2):CD001520.

48. Hill SM, Belancio VP, Dauchy RT, Xiang S, Brimer S, Mao L, Hauch A, Lundberg PW, Summers W, Yuan L *et al*: Melatonin: an inhibitor of breast cancer. *Endocr Relat Cancer* 2015, 22(3):R183-204.

49. Hong Y, Palaksha KJ, Park K, Park S, Kim H-D, Reiter RJ, Chang K-T: Melatonin plus exercise-based neurorehabilitative therapy for spinal cord injury. *J Pineal Res* 2010, 49(3):201-209.

50. Huang KL, Lu WC, Wang YY, Hu GC, Lu CH, Lee WY, Hsu CC: Comparison of agomelatine and selective serotonin reuptake inhibitors/serotonin-norepinephrine reuptake inhibitors in major depressive disorder: A meta-analysis of head-to-head randomized clinical trials. *Aust N Z J Psychiatry* 2014, 48(7):663-671.

51. Jan JE, Wasdell MB, Reiter RJ, Weiss MD, Johnson KP, Ivanenko A, Freeman RD: Melatonin therapy of pediatric sleep disorders: Recent advances, why it works, who are the candidates and how to treat. *Curr Pediatric Rev* 2007, 3(3):214-224.

52. Jansen SL, Forbes DA, Duncan V, Morgan DG: Melatonin for cognitive impairment. *Cochrane Database Syst Rev* 2006(1).

53. Jena GMMP, Trivedi PPMS: A Review of the Use of Melatonin in Ulcerative Colitis: Experimental Evidence and New Approaches. *Inflamm Bowel Dis* 2014, 20(3):553-563.

54. Jung B, Ahmad N: Melatonin in cancer management: progress and promise. *Cancer Res* 2006, 66(20):9789-9793.

55. Karaaslan C, Suzen S: Antioxidant properties of melatonin and its potential action in diseases. *Curr Top Med Chem* 2015, 15(9):894-903.

56. Keegan LJ, Reed-Berendt R, Neilly E, Morrall MC, Murdoch-Eaton D: Effectiveness of melatonin for sleep impairment post paediatric acquired brain injury: evidence from a systematic review. *Dev Neurorehabil* 2014, 17(5):355-362.

57. Kennaway DJ: Potential safety issues in the use of the hormone melatonin in paediatrics. *J Paediatr Child Health* 2015, 51(6):584-589.

58. Kuriyama A, Honda M, Hayashino Y: Ramelteon for the treatment of insomnia in adults: a systematic review and meta-analysis. *Sleep Med* 2014, 15(4):385-392.

59. Leger D, Quera-Salva MA, Vecchierini MF, Ogrizek P, Perry CA, Dressman MA: Safety profile of tasimelteon, a melatonin MT1 and MT2 receptor agonist: pooled safety analyses from six clinical studies. *Expert Opin Drug Saf* 2015, 14(11):1673-1685.

60. Lemoine P, Zisapel N: Prolonged-release formulation of melatonin (Circadin) for the treatment of insomnia. *Expert Opin Pharmacother* 2012, 13(6):895-905.

61. Leone M, Bussone G: Melatonin in cluster headache: Rationale for use and possible therapeutic potential. *CNS Drugs* 1998, 9(1):7-16.

62. Liira J, Verbeek JH, Costa G, Driscoll TR, Sallinen M, Isotalo LK, Ruotsalainen JH: Pharmacological interventions for sleepiness and sleep disturbances caused by shift work. *Cochrane Database Syst Rev* 2014(8):Cd009776.

63. Liu J, Wang Ln: Ramelteon in the treatment of chronic insomnia: systematic review and meta-analysis. *Int J Clin Pract* 2012, 66(9):867-873.

64. Macleod MR, O'Collins T, Horky LL, Howells DW, Donnan GA: Systematic review and meta-analysis of the efficacy of melatonin in experimental stroke. *J Pineal Res* 2005, 38(1):35-41.

65. Maldonado M-DMDP, Murillo-Cabezas FMDP, Calvo J-RMDP, Lardone P-JM, Tan D-XMDP, Guerrero J-MMDP, Reiter RJMDP: Melatonin as pharmacologic support in burn patients: A proposed solution to thermal injury-related lymphocytopenia and oxidative damage. *Crit Care Med* 2007, 35(4):1177-1185.

66. Maldonado MD, Reiter RJ, Perez-San-Gregorio MA: Melatonin as a potential therapeutic agent in psychiatric illness. *Hum Psychopharmacol* 2009, 24(5):391-400.

67. Maria S, Witt-Enderby PA: Melatonin effects on bone: Potential use for the prevention and treatment for osteopenia, osteoporosis, and periodontal disease and for use in bone-grafting procedures. *J Pineal Res* 2014, 56(2):115-125.

68. Marseglia L, D'Angelo G, Barberi I, Manti S, Salpietro C, Arrigo T, Reiter RJ, Gitto E: Melatonin and atopy: Role in atopic dermatitis and asthma. *Int J Mol Sci* 2014, 15(8):13482-13493.

69. Mehta A, Kaur G: Potential role of melatonin in prevention and treatment of oral carcinoma. *Indian J Dent* 2014, 5(2):86-91.

70. McGrane IR, Leung JG, St Louis EK, Boeve BF: Melatonin therapy for REM sleep behavior disorder: a critical review of evidence. *Sleep Med* 2015, 16(1):19-26.

71. Mihara T, Nakamura N, Ka K, Oba MS, Goto T: Effects of melatonin premedication to prevent emergence agitation after general anaesthesia in children: A systematic review and meta-analysis with trial sequential analysis. *Eur J Anaesthesiol* 2015, 32(12):862-871.

72. Mills E, Wu P, Seely D, Guyatt G: Melatonin in the treatment of cancer: A systematic review of randomized controlled trials and meta-analysis. *J Pineal Res* 2005, 39(4):360-366.

73. Miroddi M, Bruno R, Galletti F, Calapai F, Navarra M, Gangemi S, Calapai G: Clinical pharmacology of melatonin in the treatment of tinnitus: a review. *Eur J Clin Pharmacol* 2015, 71(3):263-270.

74. Ma Z, Yang Y, Fan C, Han J, Wang D, Di S, Hu W, Liu D, Li X, Reiter RJ *et al*: Melatonin as a potential anticarcinogen for non-small-cell lung cancer. *Oncotarget* 2016.

75. Malhotra S, Sawhney G, Pandhi P: The therapeutic potential of melatonin: a review of the science. *MedGenMed* 2004, 6(2):46.

76. Mozaffari S, Rahimi R, Abdollahi M: Implications of melatonin therapy in irritable bowel syndrome: a systematic review. *Curr Pharm Des* 2010, 16(33):3646-3655.

77. Nduhirabandi F, du Toit EF, Lochner A: Melatonin and the metabolic syndrome: a tool for effective therapy in obesity-associated abnormalities? *Acta Physiologica* 2012, 205(2):209-223.

78. Melatonin for sleep problems in children with neurodevelopmental disorders. *Drug Ther Bull* 2015, 53(10):117-120.

79. Nowak JZ, Zawilska JB: Melatonin and its physiological and therapeutic properties. *Pharm World Sci* 1998, 20(1):18-27.

80. Olde Rikkert MG, Rigaud AS: Melatonin in elderly patients with insomnia. A systematic review. *Z Gerontol Geriatr* 2001, 34(6):491-497.

81. Panzer A, Viljoen M: The validity of melatonin as an oncostatic agent. *J Pineal Res* 1997, 22(4):184-202.

82. Romero A, Ramos E, de Los Rios C, Egea J, Del Pino J, Reiter RJ: A review of metal-catalyzed molecular damage: protection by melatonin. *J Pineal Res* 2014, 56(4):343-370.

83. Rossignol DA, Frye RE: Melatonin in autism spectrum disorders: a systematic review and meta-analysis. *Dev Med Child Neurol* 2011, 53(9):783-792.

84. Ryung Wang H, Sup Woo Y, Bahk WM: The role of melatonin and melatonin agonists in counteracting antipsychotic-induced metabolic side effects: a systematic review. *Int Clin Psychopharmacol* 2016.

85. Sajith SG, Clarke D: Melatonin and sleep disorders associated with intellectual disability: a clinical review. *J Intellect Disabil Res* 2007, 51(Pt 1):2-13.

86. Sanchez-Barcelo EJ, Cos S, Mediavilla D, Martinez-Campa C, Gonzalez A, Alonso-Gonzalez C: Melatonin-estrogen interactions in breast cancer. *J Pineal Res* 2005, 38(4):217-222.

87. Sanchez-Barcelo EJ, Mediavilla MD, Tan DX, Reiter RJ: Clinical uses of melatonin: evaluation of human trials. *Curr Med Chem* 2010, 17(19):2070-2095.

88. Sanchez-Barcelo EJ, Mediavilla MD, Alonso-Gonzalez C, Reiter RJ: Melatonin uses in oncology: breast cancer prevention and reduction of the side effects of chemotherapy and radiation. *Expert Opin Investig Drugs* 2012, 21(6):819-831.

89. Seely D, Wu P, Fritz H, Kennedy DA, Tsui T, Seely AJ, Mills E: Melatonin as adjuvant cancer care with and without chemotherapy: a systematic review and meta-analysis of randomized trials. *Integr Cancer Ther* 2012, 11(4):293-303.

90. Seko LM, Moroni RM, Leitao VM, Teixeira DM, Nastri CO, Martins WP: Melatonin supplementation during controlled ovarian stimulation for women undergoing assisted reproductive technology: systematic review and meta-analysis of randomized controlled trials. *Fertil Steril* 2014, 101(1):154-161.e154.

91. Shirazi A, Ghobadi G, Ghazi-Khansari M: A radiobiological review on melatonin: a novel radioprotector. *J Radiat Res* 2007, 48(4):263-272.

92. Singh M, Jadhav HR: Melatonin: functions and ligands. *Drug Discov Today* 2014, 19(9):1410-1418.

93. Scholtens RM, van Munster BC, van Kempen MF, de Rooij SEJA: Physiological melatonin levels in healthy older people: A systematic review. *J Psychosom Res* 2016, 86:20-27.

94. Srinivasan V, Lauterbach EC, Ho KY, Acuña-Castroviejo D, Zakaria R, Brzezinski A: Melatonin in antinociception: Its therapeutic applications. *Curr Neuropharmacol* 2012, 10(2):167-178.

95. Srinivasan V, Mohamed M, Kato H: Melatonin in bacterial and viral infections with focus on sepsis: a review. *Recent Pat Endocr Metab Immune Drug Discov* 2012, 6(1):30-39.

96. Chang YS, Lin MH, Lee JH, Lee PL, Dai YS, Chu KH, Sun C, Lin YT, Wang LC, Yu HH *et al*: Melatonin Supplementation for Children with Atopic Dermatitis and Sleep Disturbance: A Randomized Clinical Trial. *JAMA Pediatrics* 2016, 170(1):35-42.

97. Reiter RJ, Tan DX, Korkmaz A, Erren TC, Piekarski C, Tamura H, Manchester LC: Light at night, chronodisruption, melatonin suppression, and cancer risk: a review. *Crit Rev Oncog* 2007, 13(4):303-328.

98. Tamura H, Nakamura Y, Korkmaz A, Manchester LC, Tan DX, Sugino N, Reiter RJ: Melatonin and the ovary: physiological and pathophysiological implications. *Fertil Steril* 2009, 92(1):328-343.

99. Reiter RJ, Tan DX, Tamura H, Cruz MH, Fuentes-Broto L: Clinical relevance of melatonin in ovarian and placental physiology: a review. *Gynecol Endocrinol* 2014, 30(2):83-89.

100. Terry PDPMPH, Villinger FDVMP, Bubenik GAMD, Sitaraman SVMDP: Melatonin and ulcerative colitis: Evidence, biological mechanisms, and future research. *Inflamm Bowel Dis* 2009, 15(1):134-140.

101. Tordjman S, Najjar I, Bellissant E, Anderson GM, Barburoth M, Cohen D, Jaafari N, Schischmanoff O, Fagard R, Lagdas E *et al*: Advances in the research of melatonin in autism spectrum disorders: literature review and new perspectives. *Int J Mol Sci* 2013, 14(10):20508-20542.

102. Turk J: Melatonin supplementation for severe and intractable sleep disturbance in young people with genetically determined developmental disabilities: short review and commentary. *J Med Genet* 2003, 40(11):793-796.

103. van Geijlswijk IM, Korzilius HP, Smits MG: The use of exogenous melatonin in delayed sleep phase disorder: a meta-analysis. *Sleep* 2010, 33(12):1605-1614.

104. Vielma JR, Bonilla E, Chacin-Bonilla L, Mora M, Medina-Leendertz S, Bravo Y: Effects of melatonin on oxidative stress, and resistance to bacterial, parasitic, and viral infections: a review. *Acta Trop* 2014, 137:31-38.

105. Vijayalaxmi, Thomas CR, Jr., Reiter RJ, Herman TS: Melatonin: from basic research to cancer treatment clinics. *J Clin Oncol* 2002, 20(10):2575-2601.

106. Vijayalaxmi, Reiter RJ, Tan DX, Herman TS, Thomas CR, Jr.: Melatonin as a radioprotective agent: a review. *Int J Radiat Oncol Biol Phys* 2004, 59(3):639-653.

107. Vural EMS, Van Munster BC, De Rooij SE: Optimal dosages for melatonin supplementation therapy in older adults: A systematic review of current literature. *Drugs Aging* 2014, 31(6):441-451.

108. Wade A, Downie S: Prolonged-release melatonin for the treatment of Insomnia in patients over 55 years. *Expert Opin Investigat Drugs* 2008, 17(10):1567-1572.

109. Wang-Weigand S, McCue M, Ogrinc F, Mini L: Effects of ramelteon 8 mg on objective sleep latency in adults with chronic insomnia on nights 1 and 2: pooled analysis. *Curr Med Res Opin* 2009, 25(5):1209-1213.

110. Wang YM, Jin BZ, Ai F, Duan CH, Lu YZ, Dong TF, Fu QL: The efficacy and safety of melatonin in concurrent chemotherapy or radiotherapy for solid tumors: a meta-analysis of randomized controlled trials. *Cancer Chemother Pharmacol* 2012, 69(5):1213-1220.

111. Wang HR, Woo YS, Bahk WM: The role of melatonin and melatonin agonists in counteracting antipsychotic-induced metabolic side effects: a systematic review. *International Clinical Psychopharmacology* 2016, 31(6):301-306.

112. Wilhelmsen M, Amirian I, Reiter RJ, Rosenberg J, Gogenur I: Analgesic effects of melatonin: a review of current evidence from experimental and clinical studies. *J Pineal Res* 2011, 51(3):270-277.

113. Wilkinson D, Shepherd E, Wallace EM: Melatonin for women in pregnancy for neuroprotection of the fetus. *Cochrane Database Syst Rev* 2016(3).

114. Winkler A, Auer C, Doering BK, Rief W: Drug treatment of primary insomnia: A meta-analysis of polysomnographic randomized controlled trials. *CNS Drugs* 2014, 28(9):799-816.

115. Witt-Enderby PA, Radio NM, Doctor JS, Davis VL: Therapeutic treatments potentially mediated by melatonin receptors: potential clinical uses in the prevention of osteoporosis, cancer and as an adjuvant therapy. *J Pineal Res* 2006, 41(4):297-305.

116. Wright A, Diebold J, Otal J, Stoneman C, Wong J, Wallace C, Duffett M: The Effect of Melatonin on Benzodiazepine Discontinuation and Sleep Quality in Adults Attempting to Discontinue Benzodiazepines: A Systematic Review and Meta-Analysis. *Drugs Aging* 2015, 32(12):1009-1018.

117. Chen S, Shi L, Liang F, Xu L, Desislava D, Wu Q, Zhang J: Exogenous Melatonin for Delirium Prevention: a Meta-analysis of Randomized Controlled Trials. *Mol Neurobiol* 2015.

118. Yang L, Yao M, Lan Y, Mo W, Sun YL, Wang J, Wang YJ, Cui XJ: Melatonin for Spinal Cord Injury in Animal Models: A Systematic Review and Network Meta-Analysis. *J Neurotrauma* 2016, 33(3):290-300.

119. Yousaf F, Seet E, Venkatraghavan L, Abrishami A, Chung F: Efficacy and safety of melatonin as an anxiolytic and analgesic in the perioperative period : A qualitative systematic review of randomized trials. *Anesthesiology* 2010, 113(4):968-976.

120. Zhang W, Chen XY, Su SW, Jia QZ, Ding T, Zhu ZN, Zhang T: Exogenous melatonin for sleep disorders in neurodegenerative diseases: a meta-analysis of randomized clinical trials. *Neurol Sci* 2016, 37(1):57-65.
