# Supplementary material for: Melatonin and health: an umbrella review of health outcomes and biological mechanisms of action
Source: BMC Med. 2018 Feb 5;16:18. doi: 10.1186/s12916-017-1000-8 (PMC5798185; doi:10.1186/s12916-017-1000-8)
Supplement: Supplementary file 2 — Summary of studies on the effects of endogenous melatonin on health outcomes (N = 75). (DOCX 111 kb) [file 12916_2017_1000_MOESM2_ESM.docx]

**Additional file 2: Table S2. Summary of studies of the effects of endogenous MLT on health outcomes (n=75)**

| **Study (year) [Reference]** | **Number of primary studies/total N** | **QR/QPS** | **Subjects/condition/ /indication** | **Administration of melatonin (dose, route, frequency, and duration)** | **Meta-analyses (y/n)** | **Effects*/health outcomes/ overall result** | **Confounders** | **Comment** |
| --- | --- | --- | --- | --- | --- | --- | --- | --- |
| Agorastos (2016) [[1](#_ENREF_1)] | Inestimable | -9/Not evaluated | Posttraumatic stress disorder | Unknown | No | Ability to induce sleep, reduce pain, modulate stress | Hormones, diet, activity, ageing processes, chronodistruption | Narrative review; animal and human studies |
| Aranda (2017) [[2](#_ENREF_2)] | Inestimable | -9/Not evaluated | Inflammatory visual diseases | 10 mg/kg (injection) | No | Ability to reduce inflammation, oxidative and nitrosative damage | Corticosteroids | In vitro and in vivo models |
| Arendt (2003) [[3](#_ENREF_3)] | Inestimable | -9/Not evaluated | Circadian rhythm related sleep disorders | Range 0.05mg-5 mg | No | Ability to induce sleepiness or sleep | Body posture, dim light, ALAN | EN and EX MLT |
| Atkinson (2003) [[4](#_ENREF_4)] | Inestimable | -9/Not evaluated | Exercise physiology and human performance | Range: 0.1-100mg | No | Reduction in short term mental (cognitive) and physical performance | Age and fitness | EN and EX MLT; healthy individuals; hypothermic properties of MLT |
| Aversa (2012) [[5](#_ENREF_5)] | Inestimable | -9/Not evaluated | Pregnant women and newborns | Unknown | No | Improved quality of life, and reduced  healthcare costs; fewer complications | Vitamins A,C, E, β-carotene | EN and EX MLT; narrative review |
| Barron (2007) [[6](#_ENREF_6)] | 45/ Inestimable | -1/Not evaluated | Menstrual cycle parameters | 3 mg | No | MLT and light exposure affects regularity of menstrual cycles, menstrual cycle symptoms, and disordered ovarian function | Hormone levels variability, light intensity and duration, caffeine consumption, subclinical thyroid disease, polycystic ovaries | EN and EX MLT; a variety of experimental and quasi-experimental designs |
| Bartsch (1997) [[7](#_ENREF_7)] | Inestimable | -9/Not evaluated | Cancer (various) | Unknown | No | Reduction of melatonin in cancer patient | Estrogen, prolactin, somatotropin, and  TSH, tryptophan, tumour stage | Animal and human studies |
| Bartsch (2006) [[8](#_ENREF_8)] | Inestimable | -9/Not evaluated | Cancer (various) | Unknown | No | Lifestyle factors and industrialisation might negatively affect MLT production | ALAN, frequent long flights, alcohol use | In vitro and in vivo models, MLT for the prevention and treatment |
| Basler (2014) [[9](#_ENREF_9)] | 10/1650^ | -3/Not evaluated | Breast cancer | Unknown | Yes | RR=0.82, (95% CI 0.68-0.99) for breast cancer risk | Genetic and environmental factors | Prospective case-control studies |
| Benítez-King (2009) [[10](#_ENREF_10)] | Inestimable | -9/Not evaluated | Cancer | Unknown | No | Inhibition of cancer cell migration, synchronisation of water transportation | n/a | In vitro models |
| Beyer (1998) [[11](#_ENREF_11)] | Inestimable | -9/Not evaluated | Cancers, hypertension, pulmonary, and neurodegenerative diseases | Unknown | No | Prevention, deceleration, or treatment of neurodegenerative  and neoplastic diseases | n/a | EX and EN MLT; in vivo studies |
| Bizzarri (2013) [[12](#_ENREF_12)] | Inestimable | -9/Not evaluated | Cancer | Unknown | No | Induction of cell apoptosis | n/a | In vivo, animal and human models |
| Blask (2011) [[13](#_ENREF_13)] | Inestimable | -9/Not evaluated | Breast cancer | Unknown | No | Reduce/prevent human breast cancer growth through mediated circadian regulation | ALAN | Narrative review; in vitro studies |
| Boga (2012) [[14](#_ENREF_14)] | Inestimable | -9/Not evaluated | Viral infections | Range: 1 mg-500 mg | No | Prevention and treatment of viral  infections | n/a | In vitro and in vivo models |
| Bonmati-Carrion (2014) [[15](#_ENREF_15)] | Inestimable | -9/Not evaluated | Diabetes, obesity, heart disease, cognitive and affective impairment, premature aging and  cancer | Unknown | No | Positive health effects by reducing the light pollution | Chrono disruption by blue light | Narrative and speculative review |
| Chen (2011) [[16](#_ENREF_16)] | 16/547 | -9/Low | Diseases involving ileum and colon | Range: 3 mg – 40 mg/day  (for range: 2-9 weeks) | No | Reduced hypersensitivity and pain in patients with  IBS; promising results in GI cancer | Chemotherapy, aloe vera and fish oil | Case reports, RCTs, in vitro and in vivo models |
| Chen (2012) [[17](#_ENREF_17)] | 17/Inestimable | -9/Not evaluated | Neonates and children | Range: 2 -200 mg/kg/day | No | Improved health outcomes: sleep, seizure control; reduced stress | Carbamazepine | Animal and human models, RCTs, open label studies |
| Chen (2015) [[18](#_ENREF_18)] | 4/669 | 9/Moderate to high | Delirium | Range: 0.5 mg – 8 mg | Yes | RR= 0.41 (95 % CI 0.15 to 1.13) for incidence of delirium | Underlying medical conditions | *I*^2^=84% |
| Cheung (2003) [[19](#_ENREF_19)] | Inestimable | -9/Not evaluated | CNS injuries | Range: 5 to 300 mg/kg (up to 4 months) | No | Various neuroprotective effects; MLT and MLT-progestin combinations can alter pituitary-ovarian  function in women and can inhibit ovulation | n/a | EX and EN MLT were assessed |
| Cos (2000) [[20](#_ENREF_20)] | Inestimable | -9/Not evaluated | Cancer | Range: 12.5µg-500 µg/day | No | Reduced the incidence and growth of breast cancer | n/a | In vivo and in vitro models |
| Costello (2014) [[21](#_ENREF_21)] | 35/2356 | 7/High | Shift workers, jetlag sufferers, insomniacs, healthy volunteers | Range: 0.3 mg to 10.0 mg/day (Range: 5-30 days) taken in pills, capsules, patches, solutions | No | Improved insomnia in both healthy volunteers and insomniacs; and sleep efficacy | Different duration and direction of flights | Majority of trials used subjective outcome measures |
| Di Bella (2006) [[22](#_ENREF_22)] | Inestimable | -9/Not evaluated | Cancer | 1 mg/0.1 ml anhydrous ethanol 95°, 10% solution (intravenously) | No | Prevention and treatment of cancer | n/a | In vivo and animal studies |
| Dopfel (2007) [[23](#_ENREF_23)] | Inestimable | -7/Not evaluated | Cancer | Unknown | No | Prevention of cancer | Dietary factors, age, BMI, parity, night work, drug use | In vivo, animal and human studies |
| Dragojevic Dikic (2015) [[24](#_ENREF_24)] | Inestimable | -9/Not evaluated | Female reproductive functions | 3mg | No | Regulation of human reproduction | n/a | EX and EN MLT were assessed |
| Grant (2009) [[25](#_ENREF_25)] | Inestimable | -9/Not evaluated | Cancer | Unknown | No | Prevention and treatment of cancer | ALAN, tamoxifen, raloxifene or fulvestrant | In vivo and in vitro models |
| Grossman (2011) [[26](#_ENREF_26)] | 7/221 | 3/Moderate to high | Nocturnal hypertension | Range: 2 mg – 5 mg | Yes | (MD=-6.1 mmHg (95% CI -10.7 to -1.5) for SBP; (MD=-3.5 mmHg; 95% CI -6.1 to -0.9) for DBP (both for slow release MLT) | Coronary artery disease, type 1 DM | 3 parallel and 4 cross-over trials |
| Guaiana (2013) [[27](#_ENREF_27)] | 13/4495 | 9/Moderate | Major depressive disorder | Agomelatine Range: 25-50 mg/day | Yes | RR=1.01 (95% CI 0.95 to 1.08) for response to treatment; RR=0.83 (95% CI 0.68 to 1.01) for remission | 10mg/d escitalopram | All but two studies had high risk of ‘other bias’; downgraded twice on inconsistency |
| Hosseinzadeh (2016) [[28](#_ENREF_28)] | Inestimable | -9/Not evaluated | Osteoarthritis | Unknown | No | Regulation of apoptotic processes and endoplasmic reticulum and mitochondrial  activity | n/a | In vivo and in vitro studies |
| Hrenak (2015) [[29](#_ENREF_29)] | 68/ Inestimable | -9/Not evaluated | Renal protection | Range: 0.3-100 mg/kg | No | Attenuated sleep disturbances and improved iron metabolism in hemodialysed patients | n/a | Animal and human studies |
| Hu (2016) [[30](#_ENREF_30)] | Inestimable | -9/Not evaluated | Fibrosis | Range: 3 μg/100 g (body weight) to 300 μg/100 g | No | Anti-fibrotic effects, injury reduction, wound healing | Light/dark cycle | Animal models, in vitro studies |
| Hunter (2017) [[31](#_ENREF_31)] | 6/475 | -4/Not evaluated | Cancer | 7.64 ng/mL | No | Increased risk of cancer | Circadian disruption, light at night/shift work | Case-control studies |
| Jemima (2011) [[32](#_ENREF_32)] | Inestimable | -9/Not evaluated | Various clinical conditions | Unknown | No | Diminished MLT secretion in various conditions including Alzheimer’s | Diet | Healthy individuals and diseased patients |
| Jung-Hynes (2010) [[33](#_ENREF_33)] | Inestimable | -9/Not evaluated | Aging and cancer | Range: 2-10 mg/kg | No | Decreased risk of cancer | Sirtuins, alterations in circadian rhythms | EX and EN MLT were assessed in animal and humans |
| Kaminski-Hartenthaler (2015) [[34](#_ENREF_34)] | 0/0 | 9/ Not evaluated | Seasonal affective disorder | n/a | n/a | The need for well-conducted, RCTs on this topic has been suggested | n/a | Authors planned  to include studies of agomelatine versus MLT |
| Lanfumey (2013) [[35](#_ENREF_35)] | Inestimable | -9/Not evaluated | Mood disorders | Unknown | No | Improvement of depressive symptoms, immune response, motor activity and cognitive performance | Genetic,  epigenetic and environmental factors | In vitro and in vivo studies including animals and humans |
| Lee (2017) [[36](#_ENREF_36)] | Inestimable | -9/Not evaluated | Stem cells survival | Range: 20-70mg/kg (injection) | No | Improved ischemia- related organ dysfunction | n/a | In vitro and in vivo studies |
| Marrin (2013) [[37](#_ENREF_37)] | 30/193 | -1/Not evaluated | Core temperature | Range: 0.003 – 40 mg | Yes | WMD=0.21 degree Celsius (0.18–0.24) for core temperature | Site measured, sex | Age range: 18–41 years |
| Marseglia (2016) [[38](#_ENREF_38)] | 4/57 | -9/Not evaluated | Pregnant women/neonates | Range: 4 mg oral twice daily-10mg/kg daily for 5 days | No | Reduced complications during pregnancy and in the perinatal period | Hypothermia | Results of two trials have not yet been published |
| Mayo (2017) [[39](#_ENREF_39)] | Inestimable | -8/Not evaluated | Regulation of cell cycle (via interaction with sirtuins) | 100 mg/kg bw/day | No | Reduced inflammation; cytoprotection, neuroprotection, immunomodulation | Sirtuins | Indirect evidence |
| Najeeb (2016) [[40](#_ENREF_40)] | 3/180 | -8/Not evaluated | Dentistry and periodontology | Unknown | No | Positive outcomes in periodontitis, periodontal regeneration, oral  implantology | Benzocaine | No critical appraisal of studies |
| Pacchierotti (2001) [[41](#_ENREF_41)] | Inestimable | -8/Not evaluated | Psychiatric disorders | Unknown | No | Altered secretion in seasonal affective disorder, bipolar disorder, unipolar depression, bulimia,  anorexia, schizophrenia, panic disorder, obsessive compulsive disorder | Intense stress, daytime exercise | Animal and human studies |
| Pandi-Perumal (2008) [[42](#_ENREF_42)] | 17/284 | 0/Not evaluated | Circadian rhythm sleep disorders | Range: 0.05 mg – 10 mg | No | Beneficial for treating disturbed sleep disorders | Low levels of EN MLT; light or posture; night shift work | EX and EN MLT were assessed; mainly human trials |
| Pandi-Perumal, (2008) [[43](#_ENREF_43)] | Inestimable | -9/Not evaluated | Cancers | Range: 0.5 mg - 50 mg orally for 4-6 weeks | No | Effective in advanced tumours of  The GI track | **n/a** | In in vitro and human studies; review of location of MLT receptors |
| Paul (2015) [[44](#_ENREF_44)] | Inestimable | -9/Not evaluated | Various health conditions | 50mg/kg | No | Improved health outcomes; prevention of diseases and disorders of elderly | Homocysteine | EX and EN MLT for diseases treatment and prevention; animal, human studies |
| Phillips (2004) [[45](#_ENREF_45)] | 3/35 | 2/Not evaluated | Children with neuro-developmental disabilities and sleep impairment | Range: 0.5 mg 0 5 mg | No | Increased sleep time, decreased awakenings & significant reduction in sleep latency in two studies | No wash-out period in one study | All three trials were of cross-over design |
| Pytka (2017) [[46](#_ENREF_46)] | Inestimable | -9/Not evaluated | Depression | Unknown | No | Reduction of depression | n/a | Animal models; MLT agonists also evaluated |
| Ramis (2015) [[47](#_ENREF_47)] | Inestimable | -9/Not evaluated | Various health conditions | Up to 50 mg/kg | No | Reduction of inflammation, oxidative stress | Tryptamine, tryptophan, indole-3- carboxylic acid, | In vivo and in vitro models; EX and EN MLT |
| Ramos (2017) [[48](#_ENREF_48)] | Inestimable | -9/Not evaluated | Ischemic brain injury | Range: 2.5-10mg/kg | No | Antioxidant, anti-inflammatory, neuromodulatory and antiexcitotoxic effects | n/a | Mainly animal studies |
| Reiter (2000) [[49](#_ENREF_49)] | Inestimable | -9/Not evaluated | Neurodegenerative diseases, cancer, ischemia/reperfusion injury and aging | Unknown | No | Reduction of oxidative stress | n/a | In vivo models |
| Reiter (2001) [[50](#_ENREF_50)] | Inestimable | -9/Not evaluated | CNS injuries | Unknown | No | Reduction of oxidative damage in the brain | n/a | In vivo and in vitro models |
| Reiter (2001) [[51](#_ENREF_51)] | Inestimable | -9/Not evaluated | Various diseases, ageing | Unknown | No | Direct and indirect anti-oxidative effects | n/a | In vivo and in vitro models |
| Reiter (2003) [[52](#_ENREF_52)] | Inestimable | -9/Not evaluated | Various health conditions | Range: 250 μg to 10 mg | No | Anti-inflammatory, anti-oxidative, anti-adhesive effects | ALAN | In vivo and in vitro models |
| Reiter (2003) [[53](#_ENREF_53)] | Inestimable | -9/Not evaluated | Ischemia/reperfusion injury (stroke) | Range: 2.5 mg/kg - 10mg/kg | No | Reduced brain damage, infarct volume, and improved neurophysiologic outcomes | n/a | Animal and in vitro studies |
| Reiter (2004) [[54](#_ENREF_54)] | Inestimable | -9/Not evaluated | Dementias | Range: 1.5- 50 mg/kg | No | Beneficial for prevention and treatment of dementias | Vitamins C and E | Small, uncontrolled trials in humans; majority of animal studies |
| Reiter (2005) [[55](#_ENREF_55)] | Inestimable | -9/Not evaluated | CNS injuries | Range: 5 to 10 mg/kg | No | MLT reduces neurophysiological deficits, infarct volume, and death in animals | n/a | EX and EN MLT were assessed in various models |
| Reiter (2007) [[56](#_ENREF_56)] | Inestimable | -9/Not evaluated | Cancer | Unknown | No | Increased risk of cancer | Chronodisruption and nocturnal MLT inhibition | Epidemiological and experimental studies |
| Reiter (2009) [[57](#_ENREF_57)] | Inestimable | -9/Not evaluated | Psychiatric disorders | Range: 2-9mg (duration 9-18 month) | No | Reduction of neural damage; improved psychological well-being | Vitamin E; poor sleep hygiene | EX and EN MLT were assessed in animal and human model |
| Reiter (2012) [[58](#_ENREF_58)] | 30 humans & inestimable for animals | -9/Not evaluated | Obesity and metabolic syndrome | 5 mg for 2 months (human) | No | Anti-obesity, anti-diabetic actions, and blood pressure-reducing effects | Circadian chronodisruption, sleep deficiency | In-vivo and ex-vivo models, open-label study |
| Reiter (2014) [[59](#_ENREF_59)] | Inestimable | -9/Not evaluated | Female reproductive functions | 3 mg | No | Enhancement  of oocyte maturation and preservation of oocyte quality | n/a | Animal and human models |
| Reiter (2014) [[60](#_ENREF_60)] | 5/Inestimable | -9/Not evaluated | Hepatobiliary system | 10 mg/kg | No | Beneficial for biliary tract and liver function | n/a | Animal studies |
| Rodriguez (2009) [[61](#_ENREF_61)] | 4/Inestimable | -9/Not evaluated | Acute coronary syndrome | Unknown | No | MLT has positive effects on the cardiovascular system | n/a | Narrative review; no pooled estimates |
| Rondanelli (2013) [[62](#_ENREF_62)] | 59/Inestimable | -7/Not evaluated | Various cancers | Range: 6-50mg/day | No | Tumour remission, greater survival; better QOL, sleep and mood | n/a | EX and EN MLT for both prevention and treatment using in vitro, animal and human studies |
| Samantaray (2009) [[63](#_ENREF_63)] | Inestimable | -9/Not evaluated | CNS and PNS injuries | Range: 10 to 250 mg/kg (up to 10 days) | No | Reduction of glutamate and free radicals | n/a | EX and EN MLT; animal models |
| Scholtens (2016) [[64](#_ENREF_64)] | 19/361 | 6/Moderate | Healthy adults | Range: 8.9 pg ml^−1^ to 40.1 pg ml^−1^ | No | Max MLT level in studies with people ME aged 65–70 y was 49.3 pg ml−1 and in studies with people ME aged ≥75 y 27.8 pg ml−1; p < 0.001. | n/a | Case control studies were included |
| Shiu (2007) [[65](#_ENREF_65)] | Inestimable | -9/Not evaluated | Prostate  cancer | Unknown | No | Prostate cancer chemoprevention | n/a | EX and EN MLT for prevention and treatment; in vitro and vivo models; no critical appraisal of literature |
| Su (2017) [[66](#_ENREF_66)] | Inestimable | -8/Not evaluated | Cancer | Unknown | No | Regression of cancer progression/inhibition of metastasis | Chemo and radiotherapy | In vitro studies |
| Tain (2017) [[67](#_ENREF_67)] | Inestimable | -8/Not evaluated | Cardiovascular diseases | Range: 40 µg/mL to 1 mg/kg | No | Prevention of hypertension | Diet | Animal models |
| Tan (2011) [[68](#_ENREF_68)] | 15/Inestimable | -9/Not evaluated | Obesity | Range: 1 mg kg^-1^ to 36 mg/kg | No | Weight reduction | ALAN | EX and EN MLT; animal models |
| Valenzuela (2015) [[69](#_ENREF_69)] | Inestimable | -9/Not evaluated | Pregnancy | Unknown | No | Reduction of complications during pregnancy (preterm delivery, preeclampsia) | MLT production by placenta and lymphocytes | In vitro, animal and human models |
| Xin (2009) [[70](#_ENREF_70)] | Inestimable | -9/Not evaluated | Neurodegenerative disorders | Up to 300mg/day (per rectum) | No | Protection against neurodegeneration in Alzheimer’s,  Parkinson's, Huntington’s disease, and Amyotrophic Lateral Sclerosis | n/a | In vitro, animal, human models |
| Xin (2015) [[71](#_ENREF_71)] | 9/Inestimable | -9/Not evaluated | GI cancers | Range: 20-40 mg/day | No | Anti-gastrointestinal  cancer properties | ALAN, oxaliplatin, 5-fluorouracil, folinic acid, cisplatin, epirubicin, leucovorin, and interleukin 2 were used in clinical trials | In vitro, animal models, clinical trials |
| Yang (2014) [[72](#_ENREF_72)] | 9/Inestimable | -9/Not evaluated | Heart diseases | Unknown | No | Protection against myocardial infarction, hypertension, vascular endothelial dysfunction, arrhythmias, cardiotoxicity | n/a | Case–control study; EX and EN MLT |
| Yang (2014) [[72](#_ENREF_72), [73](#_ENREF_73)] | 6/1237## | 8/High | Cancer | Unknown | Yes | RR =0.86 (95% CI 0.78 to 0.95) for reduced breast cancer risk | ALAN, sleep duration, menopausal status | *I*^2^= 46.4% |
| Zetner (2016) [[74](#_ENREF_74)] | 37/Inestimable | 1/Not evaluated | Protection against radiation injuries | Range: 0.1 – 250 mg/kg | No | Protection against radiation enteritis | n/a | Experimental case-control animal studies |
| Zhang (2017) [[75](#_ENREF_75)] | Inestimable | -9/Not evaluated | Liver injuries and diseases | Range: 0.5 mM - 3 g/kg | No | Protective effects against liver injuries and diseases | Diet, exercise, light exposure | Human, animal and in vitro studies |

**Table 6** Footnote: ^- for the pooled data; ##- number of cases only; ALAN- artificial light at night; CNS-Central Nervous System; DNA- deoxyribonucleic acid; EN-endogenous; EX-exogenous; GI-gastrointestinal; ME-mean; mg-milligram; MLT-melatonin;

pg- pictogram/millilitre; PNS- peripheral nervous system; QOL-quality of life; QPS- quality of primary studies (as evaluated by the authors of primary studies); QR- quality of the reviews (Oxman score, please refer also to additional Table 7); TSH- thyroid-stimulating hormone

**References**

1. Agorastos A, Linthorst ACE: Potential pleiotropic beneficial effects of adjuvant melatonergic treatment in posttraumatic stress disorder. *Journal of Pineal Research: Molecular, Biological, Physiological and Clinical Aspects of Melatonin* 2016, 61(1):3-26.

2. Aranda ML, Fleitas MF, Dieguez H, Iaquinandi A, Sande PH, Dorfman D, Rosenstein RE: Melatonin As A Therapeutic Resource For Inflammatory Visual Diseases. *Current Neuropharmacology* 2017, 13:13.

3. Arendt J: Importance and Relevance of Melatonin to Human Biological Rhythms. *J Neuroendocrinol* 2003, 15(4):427-431.

4. Atkinson G, Drust B, Reilly T, Waterhouse J: The Relevance of Melatonin to Sports Medicine and Science. *Sports Med* 2003, 33(11):809-831.

5. Aversa S, Pellegrino S, Barberi I, Reiter RJ, Gitto E: Potential utility of melatonin as an antioxidant during pregnancy and in the perinatal period. *J Matern Fetal Neonatal Med* 2012, 25(3):207-221.

6. Barron ML: Light exposure, melatonin secretion, and menstrual cycle parameters: An integrative review. *Biol Res Nurs* 2007, 9(1):49-69.

7. Bartsch C, Bartsch H: Melatonin in cancer patients and in tumor-bearing animals. *Adv Exp Med Biol* 1999, 467:247-264.

8. Bartsch C, Bartsch H: The anti-tumor activity of pineal melatonin and cancer enhancing life styles in industrialized societies. *Cancer Causes Control* 2006, 17(4):559-571.

9. Basler M, Jetter A, Fink D, Seifert B, Kullak-Ublick GA, Trojan A: Urinary excretion of melatonin and association with breast cancer: meta-analysis and review of the literature. *Breast Care (Basel)* 2014, 9(3):182-187.

10. Benitez-King G, Soto-Vega E, Ramirez-Rodriguez G: Melatonin modulates microfilament phenotypes in epithelial cells: implications for adhesion and inhibition of cancer cell migration. *Histol Histopathol* 2009, 24(6):789-799.

11. Beyer CE, Steketee JD, Saphier D: Antioxidant properties of melatonin - An emerging mystery. *Biochem Pharmacol* 1998, 56(10):1265-1272.

12. Bizzarri M, Proietti S, Cucina A, Reiter RJ: Molecular mechanisms of the pro-apoptotic actions of melatonin in cancer: a review. *Expert Opin Ther Targets* 2013, 17(12):1483-1496.

13. Blask DE, Hill SM, Dauchy RT, Xiang S, Yuan L, Duplessis T, Mao L, Dauchy E, Sauer LA: Circadian regulation of molecular, dietary, and metabolic signaling mechanisms of human breast cancer growth by the nocturnal melatonin signal and the consequences of its disruption by light at night. *J Pineal Res* 2011, 51(3):259-269.

14. Boga JA, Coto-Montes A, Rosales-Corral SA, Tan D-X, Reiter RJ: Beneficial actions of melatonin in the management of viral infections: a new use for this "molecular handyman"? *Rev Med Virol* 2012, 22(5):323-338.

15. Bonmati-Carrion MA, Arguelles-Prieto R, Martinez-Madrid MJ, Reiter R, Hardeland R, Rol MA, Madrid JA: Protecting the melatonin rhythm through circadian healthy light exposure. *Int J Mol Sci* 2014, 15(12):23448-23500.

16. Chen CQ, Fichna J, Bashashati M, Li YY, Storr M: Distribution, function and physiological role of melatonin in the lower gut. *World J Gastroenterol* 2011, 17(34):3888-3898.

17. Chen YC, Tain YL, Sheen JM, Huang LT: Melatonin utility in neonates and children. *J Formos Med Assoc* 2012, 111(2):57-66.

18. Chen S, Shi L, Liang F, Xu L, Desislava D, Wu Q, Zhang J: Exogenous Melatonin for Delirium Prevention: a Meta-analysis of Randomized Controlled Trials. *Mol Neurobiol* 2015.

19. Cheung RT: The utility of melatonin in reducing cerebral damage resulting from ischemia and reperfusion. *J Pineal Res* 2003, 34(3):153-160.

20. Cos S, Sanchez-Barcelo EJ: Melatonin, experimental basis for a possible application in breast cancer prevention and treatment. *Histol Histopathol* 2000, 15(2):637-647.

21. Costello RB, Lentino CV, Boyd CC, O'Connell ML, Crawford CC, Sprengel ML, Deuster PA: The effectiveness of melatonin for promoting healthy sleep: A rapid evidence assessment of the literature. *Nutrition Journal* 2014:106.

22. Di Bella L, Gualano L: Key aspects of melatonin physiology: thirty years of research. *Neuro Endocrinol Lett* 2006, 27(4):425-432.

23. Dopfel RP, Schulmeister K, Schernhammer ES: Nutritional and lifestyle correlates of the cancer-protective hormone melatonin. *Cancer Detect Prev* 2007, 31(2):140-148.

24. Dragojevic Dikic S, Jovanovic AM, Dikic S, Jovanovic T, Jurisic A, Dobrosavljevic A: Melatonin: a "Higgs boson" in human reproduction. *Gynecol Endocrinol* 2015, 31(2):92-101.

25. Grant SG, Melan MA, Latimer JJ, Witt-Enderby PA: Melatonin and breast cancer: cellular mechanisms, clinical studies and future perspectives. *Expert Rev Mol Med* 2009, 11:e5.

26. Grossman E, Laudon M, Zisapel N: Effect of melatonin on nocturnal blood pressure: meta-analysis of randomized controlled trials. *Vasc Health Risk Manag* 2011, 7:577-584.

27. Guaiana G, Gupta S, Chiodo D, Davies SJ, Haederle K, Koesters M: Agomelatine versus other antidepressive agents for major depression. *Cochrane Database Syst Rev* 2013(12):Cd008851.

28. Hosseinzadeh A, Kamrava SK, Joghataei MT, Darabi R, Shakeri‐Zadeh A, Shahriari M, Reiter RJ, Ghaznavi H, Mehrzadi S: Apoptosis signaling pathways in osteoarthritis and possible protective role of melatonin. *Journal of Pineal Research: Molecular, Biological, Physiological and Clinical Aspects of Melatonin* 2016, 61(4):411-425.

29. Hrenak J, Paulis L, Repova K, Aziriova S, Nagtegaal EJ, Reiter RJ, Simko F: Melatonin and renal protection: Novel perspectives from animal experiments and human studies (review). *Curr Pharm Des* 2015, 21(7):936-949.

30. Hu W, Ma Z, Jiang S, Fan C, Deng C, Yan X, Di S, Lv J, Reiter RJ, Yang Y: Melatonin: The dawning of a treatment for fibrosis? *Journal of Pineal Research: Molecular, Biological, Physiological and Clinical Aspects of Melatonin* 2016, 60(2):121-131.

31. Hunter CM, Figueiro MG: Measuring Light at Night and Melatonin Levels in Shift Workers: A Review of the Literature. *Biological Research for Nursing* 2017, 19(4):365-374.

32. Jemima J, Bhattacharjee P, Singhal RS: Melatonin - a review on the lesser known potential nutraceutical. *Int J Pharm Sci Res* 2011, 2(8):1975-1987.

33. Jung-Hynes B, Reiter RJ, Ahmad N: Sirtuins, melatonin and circadian rhythms: building a bridge between aging and cancer. *J Pineal Res* 2010, 48(1):9-19.

34. KaminskiHartenthaler A, Nussbaumer B, Forneris CA, Morgan LC, Gaynes BN, Sonis JH, Greenblatt A, Wipplinger J, Lux LJ, Winkler D *et al*: Melatonin and agomelatine for preventing seasonal affective disorder. *Cochrane Database Syst Rev* 2015(11).

35. Lanfumey L, Mongeau R, Hamon M: Biological rhythms and melatonin in mood disorders and their treatments. *Pharmacol Ther* 2013, 138(2):176-184.

36. Lee MS, Yin T-C, Sung P-H, Chiang JY, Sun C-K, Yip H-K: Melatonin enhances survival and preserves functional integrity of stem cells: A review. *Journal of Pineal Research* 2017, 62(2):n/a-n/a.

37. Marrin K, Drust B, Gregson W, Atkinson G: A meta-analytic approach to quantify the dose-response relationship between melatonin and core temperature. *Eur J Appl Physiol* 2013, 113(9):2323-2329.

38. Marseglia L, D'Angelo G, Manti S, Reiter RJ, Gitto E: Potential Utility of Melatonin in Preeclampsia, Intrauterine Fetal Growth Retardation, and Perinatal Asphyxia. *Reproductive Sciences* 2016, 23(8):970-977.

39. Mayo JC, Sainz RM, González Menéndez P, Cepas V, Tan DX, Reiter RJ: Melatonin and sirtuins: A 'not‐so unexpected' relationship. *Journal of Pineal Research: Molecular, Biological, Physiological and Clinical Aspects of Melatonin* 2017, 62(2):1-17.

40. Najeeb S, Khurshid Z, Zohaib S, Zafar MS: Therapeutic potential of melatonin in oral medicine and periodontology. *Kaohsiung Journal of Medical Sciences* 2016, 32(8):391-396.

41. Pacchierotti C, Iapichino S, Bossini L, Pieraccini F, Castrogiovanni P: Melatonin in psychiatric disorders: a review on the melatonin involvement in psychiatry. *Front Neuroendocrinol* 2001, 22(1):18-32.

42. Pandi-Perumal SR, Trakht I, Spence DW, Srinivasan V, Dagan Y, Cardinali DP: The roles of melatonin and light in the pathophysiology and treatment of circadian rhythm sleep disorders. *Nat Clin Pract Neurol* 2008, 4(8):436-447.

43. Pandi-Perumal SR, Trakht I, Srinivasan V, Spence DW, Maestroni GJ, Zisapel N, Cardinali DP: Physiological effects of melatonin: role of melatonin receptors and signal transduction pathways. *Prog Neurobiol* 2008, 85(3):335-353.

44. Paul R, Borah A: The potential physiological crosstalk and interrelationship between two sovereign endogenous amines, melatonin and homocysteine. *Life Sci* 2015, 139:97-107.

45. Phillips L, Appleton RE: Systematic review of melatonin treatment in children with neurodevelopmental disabilities and sleep impairment. *Dev Med Child Neurol* 2004, 46(11):771-775.

46. Pytka K, Mlyniec K, Podkowa K, Podkowa A, Jakubczyk M, Zmudzka E, Lustyk K, Sapa J, Filipek B: The role of melatonin, neurokinin, neurotrophic tyrosine kinase and glucocorticoid receptors in antidepressant-like effect. *Pharmacological Reports* 2017, 69(3):546-554.

47. Ramis MR, Esteban S, Miralles A, Tan DX, Reiter RJ: Protective Effects of Melatonin and Mitochondria-targeted Antioxidants Against Oxidative Stress: A Review. *Curr Med Chem* 2015, 22(22):2690-2711.

48. Ramos E, Patino P, Reiter RJ, Gil-Martin E, Marco-Contelles J, Parada E, los Rios CD, Romero A, Egea J: Ischemic brain injury: New insights on the protective role of melatonin. *Free Radical Biology and Medicine* 2017, 104:32-53.

49. Reiter RJ, Tan DX, Osuna C, Gitto E: Actions of melatonin in the reduction of oxidative stress. A review. *J Biomed Sci* 2000, 7(6):444-458.

50. Reiter RJ, Acuna-Castroviejo D, Tan DX, Burkhardt S: Free radical-mediated molecular damage. Mechanisms for the protective actions of melatonin in the central nervous system. *Ann N Y Acad Sci* 2001, 939:200-215.

51. Reiter RJ, Tan DX, Manchester LC, Qi W: Biochemical reactivity of melatonin with reactive oxygen and nitrogen species: a review of the evidence. *Cell Biochem Biophys* 2001, 34(2):237-256.

52. Reiter RJ: Melatonin: clinical relevance. *Best Pract Res Clin Endocrinol Metab* 2003, 17(2):273-285.

53. Reiter RJ, Sainz RM, Lopez-Burillo S, Mayo JC, Manchester LC, Tan DX: Melatonin Ameliorates Neurologic Damage and Neurophysiologic Deficits in Experimental Models of Stroke. *Ann N Y Acad Sci* 2003, 993:35-47.

54. Reiter RJ, Tan D-X, Pappolla MA: Melatonin Relieves the Neural Oxidative Burden that Contributes to Dementias. *Ann N Y Acad Sci* 2004:179-196.

55. Reiter RJ, Tan DX, Leon J, Kilic U, Kilic E: When melatonin gets on your nerves: its beneficial actions in experimental models of stroke. *Exp Biol Med (Maywood)* 2005, 230(2):104-117.

56. Reiter RJ, Tan DX, Korkmaz A, Erren TC, Piekarski C, Tamura H, Manchester LC: Light at night, chronodisruption, melatonin suppression, and cancer risk: a review. *Crit Rev Oncog* 2007, 13(4):303-328.

57. Reiter RJ, Benitez-King G: Melatonin reduces neuronal loss and cytoskeletal deterioration: Implications for psychiatry. *Salud Mental* 2009, 32(1):3-11.

58. Reiter RJ, Tan DX, Korkmaz A, Ma S: Obesity and metabolic syndrome: Association with chronodisruption, sleep deprivation, and melatonin suppression. *Ann Med* 2012, 44(6):564-577.

59. Reiter RJ, Tan DX, Tamura H, Cruz MH, Fuentes-Broto L: Clinical relevance of melatonin in ovarian and placental physiology: a review. *Gynecol Endocrinol* 2014, 30(2):83-89.

60. Reiter RJ, Rosales-Corral SA, Manchester LC, Liu X, Tan DX: Melatonin in the biliary tract and liver: Health implications. *Curr Pharm Des* 2014, 20(30):4788-4801.

61. Rodríguez AD: Melatonin in the acute coronary syndromes. *Salud(i)Ciencia* 2007, 15(6):983-985.

62. Rondanelli M, Faliva MA, Perna S, Antoniello N: Update on the role of melatonin in the prevention of cancer tumorigenesis and in the management of cancer correlates, such as sleep-wake and mood disturbances: Review and remarks. *Aging Clin Exp Res* 2013, 25(5):499-510.

63. Samantaray S, Das A, Thakore NP, Matzelle DD, Reiter RJ, Ray SK, Banik NL: Therapeutic potential of melatonin in traumatic central nervous system injury. *J Pineal Res* 2009, 47(2):134-142.

64. Scholtens RM, van Munster BC, van Kempen MF, de Rooij SEJA: Physiological melatonin levels in healthy older people: A systematic review. *J Psychosom Res* 2016, 86:20-27.

65. Shiu SYW: Towards rational and evidence-based use of melatonin in prostate cancer prevention and treatment. *J Pineal Res* 2007, 43(1):1-9.

66. Su SC, Hsieh MJ, Yang WE, Chung WH, Reiter RJ, Yang SF: Cancer metastasis: Mechanisms of inhibition by melatonin. *Journal of Pineal Research: Molecular, Biological, Physiological and Clinical Aspects of Melatonin* 2017, 62(1):1-11.

67. Tain YL, Huang LT, Hsu CN: Developmental Programming of Adult Disease: Reprogramming by Melatonin? *International Journal of Molecular Sciences* 2017, 18(2):16.

68. Tan DX, Manchester LC, Fuentes-Broto L, Paredes SD, Reiter RJ: Significance and application of melatonin in the regulation of brown adipose tissue metabolism: Relation to human obesity. *Obes Rev* 2011, 12(3):167-188.

69. Valenzuela FJ, Vera J, Venegas C, Pino F, Lagunas C: Circadian System and Melatonin Hormone: Risk Factors for Complications during Pregnancy. *Obstet Gynecol Int* 2015, 2015:825802.

70. Wang X: The Antiapoptotic Activity of Melatonin in Neurodegenerative Diseases. *CNS: Neurosci Ther* 2009, 15(4):345-357.

71. Xin Z, Jiang S, Jiang P, Yan X, Fan C, Di S, Wu G, Yang Y, Reiter RJ, Ji G: Melatonin as a treatment for gastrointestinal cancer: a review. *J Pineal Res* 2015, 58(4):375-387.

72. Yang Y, Sun Y, Yi W, Li Y, Fan C, Xin Z, Jiang S, Di S, Qu Y, Reiter RJ *et al*: A review of melatonin as a suitable antioxidant against myocardial ischemia-reperfusion injury and clinical heart diseases. *J Pineal Res* 2014, 57(4):357-366.

73. Yang WS, Deng Q, Fan WY, Wang WY, Wang X: Light exposure at night, sleep duration, melatonin, and breast cancer: A dose-response analysis of observational studies. *Eur J Cancer Prev* 2014, 23(4):269-276.

74. Zetner D, Andersen LP, Rosenberg J: Melatonin as Protection Against Radiation Injury: A Systematic Review. *Drug Res (Stuttg)* 2016, 66(6):281-296.

75. Zhang JJ, Meng X, Li Y, Zhou Y, Xu DP, Li S, Li HB: Effects of Melatonin on Liver Injuries and Diseases. *International Journal of Molecular Sciences* 2017, 18(4):23.
