# Supplementary material for: Melatonin and health: an umbrella review of health outcomes and biological mechanisms of action
Source: BMC Med. 2018 Feb 5;16:18. doi: 10.1186/s12916-017-1000-8 (PMC5798185; doi:10.1186/s12916-017-1000-8)
Supplement: Supplementary file 3 — Quality ratings for included systematic reviews of melatonin for health. (DOCX 254 kb) [file 12916_2017_1000_MOESM3_ESM.docx]

**Additional file 3: Table S3. Quality ratings for included SRs of MLT for health**

| **Study (Year)**  **[Reference]** | **Search**  **Methods?**  **(a)** | **Search Comprehensive?**  **(b)** | **Inclusion Criteria?**  **(c)** | **Bias**  **Avoided?**  **(d)** | **Validity**  **Criteria?**  **(e)** | **Validity**  **Assessed?**  **(f)** | **Methods for**  **Combining**  **Studies?**  **(g)** | **Appropriately**  **Combined?**  **(h)** | **Conclusions**  **Supported?**  **(i)** | **Sum** |
| --- | --- | --- | --- | --- | --- | --- | --- | --- | --- | --- |
| Agorastos (2016) [[1](#_ENREF_1)] | -1 | -1 | -1 | -1 | -1 | -1 | -1 | -1 | -1 | **-9** |
| Andersen (2014) [[2](#_ENREF_2)] | 1 | 1 | 1 | 1 | 1 | 1 | 1 | 0 | 1 | **8** |
| Anderson (2012) [[3](#_ENREF_3)] | 1 | 1 | 0 | -1 | -1 | -1 | -1 | -1 | 0 | **-3** |
| Aranda (2017) [[4](#_ENREF_4)] | -1 | -1 | -1 | -1 | -1 | -1 | -1 | -1 | -1 | **-9** |
| Arendt (2003) [[5](#_ENREF_5)] | -1 | -1 | -1 | -1 | -1 | -1 | -1 | -1 | -1 | **-9** |
| Armour (2004) [[6](#_ENREF_6)] | 1 | -1 | -1 | -1 | -1 | -1 | -1 | -1 | -1 | **-7** |
| Arora (2016) [[7](#_ENREF_7)] | 1 | 1 | 1 | 1 | 1 | 1 | 1 | 0 | 1 | **8** |
| Atkinson (2003) [[8](#_ENREF_8)] | -1 | -1 | -1 | -1 | -1 | -1 | -1 | -1 | -1 | **-9** |
| Aversa (2012) [[9](#_ENREF_9)] | -1 | -1 | -1 | -1 | -1 | -1 | -1 | -1 | -1 | **-9** |
| Bartsch (1997) [[10](#_ENREF_10)] | -1 | -1 | -1 | -1 | -1 | -1 | -1 | -1 | -1 | **-9** |
| Bartsch (2006) [[11](#_ENREF_11)] | -1 | -1 | -1 | -1 | -1 | -1 | -1 | -1 | -1 | **-9** |
| Barron (2007) [[12](#_ENREF_12)] | 1 | 1 | 0 | -1 | 0 | 0 | -1 | -1 | 0 | **-1** |
| Basler (2014) [[13](#_ENREF_13)] | 0 | -1 | 0 | -1 | -1 | 0 | 0 | 0 | 0 | **-3** |
| Bellon (2006) [[14](#_ENREF_14)] | 1 | 1 | 1 | -1 | -1 | -1 | -1 | 1 | 1 | **1** |
| Bendz (2010) [[15](#_ENREF_15)] | 1 | 1 | -1 | -1 | -1 | -1 | -1 | -1 | -1 | **-5** |
| Benítez-King (2009) [[16](#_ENREF_16)] | -1 | -1 | -1 | -1 | -1 | -1 | -1 | -1 | -1 | **-9** |
| Beyer (1998) [[17](#_ENREF_17)] | -1 | -1 | -1 | -1 | -1 | -1 | -1 | -1 | -1 | **-9** |
| Biran (2014) [[18](#_ENREF_18)] | -1 | -1 | -1 | -1 | -1 | -1 | -1 | -1 | -1 | **-9** |
| Bizzarri (2013) [[19](#_ENREF_19)] | -1 | -1 | -1 | -1 | -1 | -1 | -1 | -1 | -1 | **-9** |
| Blask (2011) [[20](#_ENREF_20)] | -1 | -1 | -1 | -1 | -1 | -1 | -1 | -1 | -1 | **-9** |
| Boga (2012) [[21](#_ENREF_21)] | -1 | -1 | -1 | -1 | -1 | -1 | -1 | -1 | -1 | **-9** |
| Bonmati-Carrion (2014) [[22](#_ENREF_22)] | -1 | -1 | -1 | -1 | -1 | -1 | -1 | -1 | -1 | **-9** |
| Bonnefont-Rousselot (2010) [[23](#_ENREF_23)] | -1 | -1 | -1 | -1 | -1 | -1 | -1 | -1 | -1 | **-9** |
| Braam (2009) [[24](#_ENREF_24)] | 1 | 1 | 0 | 0 | 1 | 1 | 1 | 0 | 1 | **6** |
| Brigo (2016) [[25](#_ENREF_25)] | 1 | 1 | 1 | 1 | 1 | 1 | 1 | 1 | 1 | **9** |
| Brzezinski (1998) [[26](#_ENREF_26)] | -1 | -1 | -1 | -1 | -1 | -1 | -1 | -1 | -1 | **-9** |
| Brzezinski (2005) [[27](#_ENREF_27)] | 1 | 0 | 1 | 1 | 0 | 1 | 1 | 1 | 1 | **7** |
| Bubenik (1998) [[28](#_ENREF_28)] | -1 | -1 | -1 | -1 | -1 | -1 | -1 | -1 | -1 | **-9** |
| Buscemi (2005) [[29](#_ENREF_29)] | 1 | 1 | 1 | 1 | 1 | 1 | 1 | 1 | 1 | **9** |
| Buscemi (2006) [[30](#_ENREF_30)] | 1 | 1 | 1 | 1 | 1 | 1 | 1 | 1 | 1 | **9** |
| Cardinali (2015) [[31](#_ENREF_31)] | 1 | 1 | -1 | -1 | -1 | -1 | -1 | -1 | -1 | **-5** |
| Carlomagno (2011) [[32](#_ENREF_32)] | -1 | -1 | -1 | -1 | -1 | -1 | -1 | -1 | -1 | **-9** |
| Carpentieri (2012) [[33](#_ENREF_33)] | -1 | -1 | -1 | -1 | -1 | -1 | -1 | -1 | -1 | **-9** |
| Carrillo-Vico (2005) [[34](#_ENREF_34)] | -1 | -1 | -1 | -1 | -1 | -1 | -1 | -1 | -1 | **-9** |
| Cervantes (2008) [[35](#_ENREF_35)] | -1 | -1 | -1 | -1 | -1 | -1 | -1 | -1 | -1 | **-9** |
| Chaplin (2008) [[36](#_ENREF_36)] | -1 | -1 | -1 | -1 | -1 | -1 | -1 | -1 | -1 | **-9** |
| Chen (2011) [[37](#_ENREF_37)] | -1 | -1 | -1 | -1 | -1 | -1 | -1 | -1 | -1 | **-9** |
| Chen (2012) [[38](#_ENREF_38)] | -1 | -1 | -1 | -1 | -1 | -1 | -1 | -1 | -1 | **-9** |
| Chen (2015) [[39](#_ENREF_39)] | 1 | 1 | 1 | 1 | 1 | 1 | 1 | 1 | 1 | **9** |
| Cheung (2003) [[40](#_ENREF_40)] | -1 | -1 | -1 | -1 | -1 | -1 | -1 | -1 | -1 | **-9** |
| Cos (2000) [[41](#_ENREF_41)] | -1 | -1 | -1 | -1 | -1 | -1 | -1 | -1 | -1 | **-9** |
| Costello (2014) [[42](#_ENREF_42)] | 1 | 1 | 1 | 1 | 1 | 1 | 0 | 0 | 1 | **7** |
| Cutando (2011) [[43](#_ENREF_43)] | 0 | 1 | -1 | -1 | -1 | -1 | -1 | -1 | -1 | **-6** |
| Cutando (2014) [[44](#_ENREF_44)] | -1 | -1 | -1 | -1 | -1 | -1 | -1 | -1 | -1 | **-9** |
| De Crescenzo (2017) [[45](#_ENREF_45)] | 1 | 1 | 1 | 1 | 1 | 1 | 1 | 0 | 1 | **8** |
| De Jonghe (2010) [[46](#_ENREF_46)] | 1 | 1 | 1 | 1 | -1 | -1 | -1 | -1 | 1 | **1** |
| De Rooij (2013) [[47](#_ENREF_47)] | -1 | -1 | -1 | -1 | -1 | -1 | -1 | -1 | -1 | **-9** |
| Di Bella (2006) [[48](#_ENREF_48)] | -1 | -1 | -1 | -1 | -1 | -1 | -1 | -1 | -1 | **-9** |
| Dopfel (2007) [[49](#_ENREF_49)] | 0 | 0 | -1 | -1 | -1 | -1 | -1 | -1 | -1 | **-7** |
| Dragojevic Dikic (2015) [[50](#_ENREF_50)] | -1 | -1 | -1 | -1 | -1 | -1 | -1 | -1 | -1 | **-9** |
| Dziegiel (2008) [[51](#_ENREF_51)] | -1 | -1 | -1 | -1 | -1 | -1 | -1 | -1 | -1 | **-9** |
| Elmahallawy (2015) [[52](#_ENREF_52)] | -1 | -1 | -1 | -1 | -1 | -1 | -1 | -1 | -1 | **-9** |
| Erdemli (2016) [[53](#_ENREF_53)] | 0 | 0 | -1 | -1 | -1 | -1 | -1 | -1 | -1 | **-7** |
| Escames (2006) [[54](#_ENREF_54)] | -1 | -1 | -1 | -1 | -1 | -1 | -1 | -1 | -1 | **-9** |
| Escames (2012) [[55](#_ENREF_55)] | -1 | -1 | -1 | -1 | -1 | -1 | -1 | -1 | -1 | **-9** |
| Favero (2014) [[56](#_ENREF_56)] | -1 | -1 | -1 | -1 | -1 | -1 | -1 | -1 | -1 | **-9** |
| Fernando (2014) [[57](#_ENREF_57)] | -1 | -1 | -1 | -1 | -1 | -1 | -1 | -1 | 0 | **-8** |
| Ferracioli-Oda (2013) [[58](#_ENREF_58)] | 1 | 1 | 1 | 1 | -1 | -1 | 1 | 1 | 1 | **5** |
| Fildes (2009) [[59](#_ENREF_59)] | -1 | -1 | -1 | -1 | -1 | -1 | -1 | -1 | -1 | **-9** |
| Giannoulia-Karantana (2006) [[60](#_ENREF_60)] | -1 | -1 | -1 | -1 | -1 | -1 | -1 | -1 | -1 | **-9** |
| Golombek (2015) [[61](#_ENREF_61)] | -1 | -1 | -1 | -1 | -1 | -1 | -1 | -1 | -1 | **-9** |
| Gomez-Moreno (2010) [[62](#_ENREF_62)] | 0 | 1 | -1 | -1 | -1 | -1 | -1 | -1 | -1 | **-6** |
| Govender (2014) [[63](#_ENREF_63)] | -1 | -1 | -1 | -1 | -1 | -1 | -1 | -1 | -1 | **-9** |
| Guaiana (2013) [[64](#_ENREF_64)] | 1 | 1 | 1 | 1 | 1 | 1 | 1 | 1 | 1 | **9** |
| Guénole (2011) [[65](#_ENREF_65)] | 1 | 1 | -1 | 0 | -1 | -1 | -1 | -1 | 0 | **-3** |
| Grant (2009) [[66](#_ENREF_66)] | -1 | -1 | -1 | -1 | -1 | -1 | -1 | -1 | -1 | **-9** |
| Grossman (2011) [[67](#_ENREF_67)] | 0 | 0 | 1 | 1 | 1 | 1 | -1 | -1 | 1 | **3** |
| Hansen (2014) [[68](#_ENREF_68)] | 1 | 1 | 1 | 1 | 1 | 1 | 1 | 0 | 1 | **8** |
| Hansen (2015) [[69](#_ENREF_69)] | 1 | 1 | 1 | 1 | 1 | 1 | 1 | 1 | 1 | **9** |
| Hardeland (2015) [[70](#_ENREF_70)] | -1 | -1 | -1 | -1 | -1 | -1 | -1 | -1 | -1 | **-9** |
| Harrod (2005) [[71](#_ENREF_71)] | -1 | -1 | -1 | -1 | -1 | -1 | -1 | -1 | -1 | **-9** |
| Hartley (2014) [[72](#_ENREF_72)] | 0 | 0 | -1 | -1 | -1 | -1 | -1 | -1 | -1 | **-7** |
| Heiligenstein (1998) [[73](#_ENREF_73)] | -1 | -1 | -1 | -1 | -1 | -1 | -1 | -1 | -1 | **-9** |
| Herxheimer (2002) [[74](#_ENREF_74)] | 1 | 1 | 1 | 1 | 1 | 1 | -1 | -1 | 1 | **5** |
| Hill (2015) [[75](#_ENREF_75)] | -1 | -1 | -1 | -1 | -1 | -1 | -1 | -1 | -1 | **-9** |
| Hong (2010) [[76](#_ENREF_76)] | -1 | -1 | -1 | -1 | -1 | -1 | -1 | -1 | -1 | **-9** |
| Hosseinzadeh (2016) [[77](#_ENREF_77)] | -1 | -1 | -1 | -1 | -1 | -1 | -1 | -1 | -1 | **-9** |
| Hrenak (2015) [[78](#_ENREF_78)] | -1 | -1 | -1 | -1 | -1 | -1 | -1 | -1 | -1 | **-9** |
| Hu (2016) [[79](#_ENREF_79)] | -1 | -1 | -1 | -1 | -1 | -1 | -1 | -1 | -1 | **-9** |
| Huang (2014) [[80](#_ENREF_80)] | 1 | 1 | 1 | 1 | 1 | 1 | 1 | 1 | 1 | **9** |
| Hunter (2017) [[81](#_ENREF_81)] | 0 | 0 | 0 | 0 | -1 | -1 | -1 | -1 | 0 | **-4** |
| Jan (2007) [[82](#_ENREF_82)] | -1 | -1 | -1 | -1 | -1 | -1 | -1 | -1 | -1 | **-9** |
| Jansen (2006) [[83](#_ENREF_83)] | 1 | 1 | 1 | 1 | 1 | 1 | 1 | 1 | 1 | **9** |
| Jemima (2011) [[84](#_ENREF_84)] | -1 | -1 | -1 | -1 | -1 | -1 | -1 | -1 | -1 | **-9** |
| Jena (2014) [[85](#_ENREF_85)] | -1 | -1 | -1 | -1 | -1 | -1 | -1 | -1 | -1 | **-9** |
| Jung (2006) [[86](#_ENREF_86)] | -1 | -1 | -1 | -1 | -1 | -1 | -1 | -1 | -1 | **-9** |
| Jung-Hynes (2010) [[87](#_ENREF_87)] | -1 | -1 | -1 | -1 | -1 | -1 | -1 | -1 | -1 | **-9** |
| Kaminski-Hartenthaler (2015) [[88](#_ENREF_88)] | 1 | 1 | 1 | 1 | 1 | 1 | 1 | 1 | 1 | **9** |
| Karaaslan (2015) [[89](#_ENREF_89)] | -1 | -1 | -1 | -1 | -1 | -1 | -1 | -1 | -1 | **-9** |
| Keegan (2014) [[90](#_ENREF_90)] | 1 | 1 | 1 | -1 | 0 | 0 | -1 | -1 | 0 | **0** |
| Kennaway (2015) [[91](#_ENREF_91)] | -1 | -1 | -1 | -1 | -1 | -1 | -1 | -1 | -1 | **-9** |
| Kuriyama (2014) [[92](#_ENREF_92)] | 1 | 1 | 1 | 1 | 1 | 1 | 1 | 1 | 1 | **9** |
| Lanfumey (2013) [[93](#_ENREF_93)] | -1 | -1 | -1 | -1 | -1 | -1 | -1 | -1 | -1 | **-9** |
| Lee (2017) [[94](#_ENREF_94)] | -1 | -1 | -1 | -1 | -1 | -1 | -1 | -1 | -1 | **-9** |
| Leger (2015) [[95](#_ENREF_95)] | -1 | -1 | -1 | -1 | -1 | -1 | -1 | -1 | -1 | **-9** |
| Lemoine (2012) [[96](#_ENREF_96)] | -1 | -1 | -1 | -1 | -1 | -1 | -1 | -1 | -1 | **-9** |
| Leone (1998) [[97](#_ENREF_97)] | -1 | -1 | -1 | -1 | -1 | -1 | -1 | -1 | -1 | **-9** |
| Liira (2014) [[98](#_ENREF_98)] | 1 | 1 | 1 | 1 | 1 | 1 | 1 | 1 | 1 | **9** |
| Liu (2012) [[99](#_ENREF_99)] | 1 | 1 | 1 | -1 | 1 | 1 | 1 | 1 | 1 | **7** |
| Ma (2016) [[100](#_ENREF_100)] | -1 | -1 | -1 | -1 | -1 | -1 | -1 | -1 | -1 | **-9** |
| Macleod (2004) [[101](#_ENREF_101)] | 1 | 1 | 0 | 0 | 1 | 1 | 1 | 1 | 1 | **7** |
| Maldonado (2007) [[102](#_ENREF_102)] | 1 | 1 | -1 | -1 | -1 | -1 | -1 | -1 | -1 | **-5** |
| Maldonado (2009) [[103](#_ENREF_103)] | -1 | -1 | -1 | -1 | -1 | -1 | -1 | -1 | -1 | **-9** |
| Malhotra (2004) [[104](#_ENREF_104)] | -1 | -1 | -1 | -1 | -1 | -1 | -1 | -1 | -1 | **-9** |
| Maria (2014) [[105](#_ENREF_105)] | -1 | -1 | -1 | -1 | -1 | -1 | -1 | -1 | -1 | **-9** |
| Marrin (2013) [[106](#_ENREF_106)] | 0 | 0 | 0 | -1 | -1 | -1 | 0 | 1 | 1 | **-1** |
| Marseglia (2014) [[107](#_ENREF_107)] | -1 | -1 | -1 | -1 | -1 | -1 | -1 | -1 | -1 | **-9** |
| Marseglia (2016) [[108](#_ENREF_108)] | -1 | -1 | -1 | -1 | -1 | -1 | -1 | -1 | 0 | **-8** |
| Mayo (2017) [[109](#_ENREF_109)] | -1 | -1 | -1 | -1 | -1 | -1 | -1 | -1 | 0 | **-8** |
| Mehta (2014) [[110](#_ENREF_110)] | 0 | 0 | -1 | -1 | -1 | -1 | -1 | -1 | -1 | **-7** |
| McGrane (2015) [[111](#_ENREF_111)] | 1 | 1 | 0 | 0 | -1 | -1 | -1 | -1 | 0 | **-2** |
| Mihara (2015) [[112](#_ENREF_112)] | 1 | 1 | 1 | 1 | 1 | 1 | 1 | 1 | 1 | **9** |
| Mills (2005) [[113](#_ENREF_113)] | 1 | 1 | 1 | -1 | -1 | -1 | 1 | 1 | -1 | **1** |
| Miroddi (2015) [[114](#_ENREF_114)] | 1 | 1 | 0 | 0 | 1 | 1 | -1 | -1 | 1 | **3** |
| Mozaffari (2010) [[115](#_ENREF_115)] | 1 | 1 | -1 | -1 | -1 | 0 | -1 | -1 | 0 | **-3** |
| Najeeb (2016) [[116](#_ENREF_116)] | -1 | -1 | -1 | -1 | -1 | -1 | -1 | -1 | 0 | **-8** |
| Nduhirabandi (2012) [[117](#_ENREF_117)] | -1 | -1 | -1 | -1 | -1 | -1 | -1 | -1 | -1 | **-9** |
| No authors listed (2015) [[118](#_ENREF_118)] | -1 | -1 | -1 | -1 | -1 | -1 | -1 | -1 | -1 | **-9** |
| Nowak (1998) [[119](#_ENREF_119)] | -1 | -1 | -1 | -1 | -1 | -1 | -1 | -1 | -1 | **-9** |
| Olde Rikkert (2001) [[120](#_ENREF_120)] | 1 | 0 | 0 | 0 | -1 | -1 | -1 | -1 | 0 | **-3** |
| Pacchierotti (2001) [[121](#_ENREF_121)] | -1 | -1 | -1 | -1 | -1 | -1 | -1 | -1 | -1 | **-9** |
| Pandi-Perumal (2008) [[122](#_ENREF_122)] | 1 | 1 | 0 | 1 | -1 | -1 | -1 | -1 | 1 | **0** |
| Pandi-Perumal, (2008) [[123](#_ENREF_123)] | -1 | -1 | -1 | -1 | -1 | -1 | -1 | -1 | -1 | **-9** |
| Panzer (1997) [[124](#_ENREF_124)] | -1 | -1 | -1 | -1 | -1 | -1 | -1 | -1 | -1 | **-9** |
| Paul (2015) [[125](#_ENREF_125)] | -1 | -1 | -1 | -1 | -1 | -1 | -1 | -1 | -1 | **-9** |
| Phillips (2004) [[126](#_ENREF_126)] | 1 | 1 | 1 | 0 | 0 | -1 | 0 | -1 | 1 | **2** |
| Pytka (2017) [[127](#_ENREF_127)] | -1 | -1 | -1 | -1 | -1 | -1 | -1 | -1 | -1 | **-9** |
| Ramis (2015) [[128](#_ENREF_128)] | -1 | -1 | -1 | -1 | -1 | -1 | -1 | -1 | -1 | **-9** |
| Ramos (2017) [[129](#_ENREF_129)] | -1 | -1 | -1 | -1 | -1 | -1 | -1 | -1 | -1 | **-9** |
| Reiter (2000) [[130](#_ENREF_130)] | -1 | -1 | -1 | -1 | -1 | -1 | -1 | -1 | -1 | **-9** |
| Reiter (2001) [[131](#_ENREF_131)] | -1 | -1 | -1 | -1 | -1 | -1 | -1 | -1 | -1 | **-9** |
| Reiter (2001) [[132](#_ENREF_132)] | -1 | -1 | -1 | -1 | -1 | -1 | -1 | -1 | -1 | **-9** |
| Reiter (2003) [[133](#_ENREF_133)] | -1 | -1 | -1 | -1 | -1 | -1 | -1 | -1 | -1 | **-9** |
| Reiter (2003) [[134](#_ENREF_134)] | -1 | -1 | -1 | -1 | -1 | -1 | -1 | -1 | -1 | **-9** |
| Reiter (2004) [[135](#_ENREF_135)] | -1 | -1 | -1 | -1 | -1 | -1 | -1 | -1 | -1 | **-9** |
| Reiter (2005) [[136](#_ENREF_136)] | -1 | -1 | -1 | -1 | -1 | -1 | -1 | -1 | -1 | **-9** |
| Reiter (2007) [[137](#_ENREF_137)] | -1 | -1 | -1 | -1 | -1 | -1 | -1 | -1 | -1 | **-9** |
| Reiter (2009) [[138](#_ENREF_138)] | -1 | -1 | -1 | -1 | -1 | -1 | -1 | -1 | -1 | **-9** |
| Reiter (2012) [[139](#_ENREF_139)] | -1 | -1 | -1 | -1 | -1 | -1 | -1 | -1 | -1 | **-9** |
| Reiter (2014) [[140](#_ENREF_140)] | -1 | -1 | -1 | -1 | -1 | -1 | -1 | -1 | -1 | **-9** |
| Reiter (2014) [[141](#_ENREF_141)] | -1 | -1 | -1 | -1 | -1 | -1 | -1 | -1 | -1 | **-9** |
| Rodriguez (2009) [[142](#_ENREF_142)] | -1 | -1 | -1 | -1 | -1 | -1 | -1 | -1 | -1 | **-9** |
| Romero (2014) [[143](#_ENREF_143)] | -1 | -1 | -1 | -1 | -1 | -1 | -1 | -1 | -1 | **-9** |
| Rondanelli (2013) [[144](#_ENREF_144)] | 0 | 0 | -1 | -1 | -1 | -1 | -1 | -1 | -1 | **-7** |
| Rossignol (2011) [[145](#_ENREF_145)] | 1 | 1 | 1 | 1 | 1 | 1 | 1 | 0 | 1 | **8** |
| Ryung Wang (2016) [[146](#_ENREF_146)] | 1 | 1 | 1 | 1 | 1 | 1 | 0 | 0 | 1 | **7** |
| Sajith (2007) [[147](#_ENREF_147)] | 1 | 1 | 0 | -1 | -1 | -1 | -1 | -1 | 0 | **-3** |
| Samantaray (2009) [[148](#_ENREF_148)] | -1 | -1 | -1 | -1 | -1 | -1 | -1 | -1 | -1 | **-9** |
| Sanchez-Barcelo (2005) [[149](#_ENREF_149)] | -1 | -1 | -1 | -1 | -1 | -1 | -1 | -1 | -1 | **-9** |
| Sanchez-Barcelo (2010) [[150](#_ENREF_150)] | -1 | -1 | -1 | -1 | -1 | -1 | -1 | -1 | -1 | **-9** |
| Sanchez-Barcelo (2012) [[151](#_ENREF_151)] | -1 | -1 | -1 | -1 | -1 | -1 | -1 | -1 | -1 | **-9** |
| Scholtens (2016) [[152](#_ENREF_152)] | 1 | 1 | 1 | 1 | 1 | 1 | 0 | -1 | 1 | **6** |
| Seely (2012) [[153](#_ENREF_153)] | 1 | 1 | 1 | 1 | 1 | 1 | 1 | 1 | 1 | **9** |
| Seko (2014) [[154](#_ENREF_154)] | 1 | 1 | 1 | 1 | 1 | 1 | 1 | 1 | 1 | **9** |
| Shirazi (2007) [[155](#_ENREF_155)] | -1 | -1 | -1 | -1 | -1 | -1 | -1 | -1 | -1 | **-9** |
| Shiu (2007) [[156](#_ENREF_156)] | -1 | -1 | -1 | -1 | -1 | -1 | -1 | -1 | -1 | **-9** |
| Singh (2014) [[157](#_ENREF_157)] | -1 | -1 | -1 | -1 | -1 | -1 | -1 | -1 | -1 | **-9** |
| Srinivasan (2009) [[158](#_ENREF_158)] | -1 | -1 | -1 | -1 | -1 | -1 | -1 | -1 | -1 | **-9** |
| Srinivasan (2012) [[159](#_ENREF_159)] | -1 | -1 | -1 | -1 | -1 | -1 | -1 | -1 | -1 | **-9** |
| Srinivasan (2012) [[160](#_ENREF_160)] | -1 | -1 | -1 | -1 | -1 | -1 | -1 | -1 | -1 | **-9** |
| Su (2017) [[161](#_ENREF_161)] | -1 | -1 | -1 | -1 | -1 | -1 | -1 | -1 | 0 | **-8** |
| Sun (2016) [[162](#_ENREF_162)] | -1 | -1 | -1 | -1 | -1 | -1 | -1 | -1 | -1 | **-9** |
| Tain (2017) [[163](#_ENREF_163)] | -1 | -1 | -1 | -1 | -1 | -1 | -1 | -1 | 0 | **-8** |
| Tamura (2008) [[164](#_ENREF_164)] | -1 | -1 | -1 | -1 | -1 | -1 | -1 | -1 | -1 | **-9** |
| Tamura (2009) [[165](#_ENREF_165)] | -1 | -1 | -1 | -1 | -1 | -1 | -1 | -1 | -1 | **-9** |
| Tamura (2014) [[166](#_ENREF_166)] | -1 | -1 | -1 | -1 | -1 | -1 | -1 | -1 | -1 | **-9** |
| Tan (2011) [[167](#_ENREF_167)] | -1 | -1 | -1 | -1 | -1 | -1 | -1 | -1 | -1 | **-9** |
| Terry (2009) [[168](#_ENREF_168)] | -1 | -1 | -1 | -1 | -1 | -1 | -1 | -1 | -1 | **-9** |
| Tordjman (2013) [[169](#_ENREF_169)] | -1 | -1 | -1 | -1 | -1 | -1 | -1 | -1 | -1 | **-9** |
| Turk (2003) [[170](#_ENREF_170)] | -1 | -1 | -1 | -1 | -1 | -1 | -1 | -1 | -1 | **-9** |
| Valenzuela (2015) [[171](#_ENREF_171)] | -1 | -1 | -1 | -1 | -1 | -1 | -1 | -1 | -1 | **-9** |
| Van Geijlswijk (2010) [[172](#_ENREF_172)] | 1 | 1 | 1 | 1 | 1 | 1 | -1 | 0 | 1 | **6** |
| Vijayalaxmi (2003) [[173](#_ENREF_173)] | -1 | -1 | -1 | -1 | -1 | -1 | -1 | -1 | -1 | **-9** |
| Vijayalaxmi (2004) [[174](#_ENREF_174)] | -1 | -1 | -1 | -1 | -1 | -1 | -1 | -1 | -1 | **-9** |
| Vielma (2014) [[175](#_ENREF_175)] | -1 | -1 | -1 | -1 | -1 | -1 | -1 | -1 | -1 | **-9** |
| Vural (2014) [[176](#_ENREF_176)] | 1 | 1 | 1 | 1 | -1 | 0 | -1 | -1 | 0 | **1** |
| Wang-Weigand (2009) [[177](#_ENREF_177)] | -1 | -1 | 0 | -1 | -1 | -1 | -1 | -1 | -1 | **-8** |
| Wang (2012) [[178](#_ENREF_178)] | 1 | 1 | 1 | 1 | 1 | 1 | 1 | 0 | 1 | **8** |
| Wang (2016) [[179](#_ENREF_179)] | 1 | 1 | 1 | 1 | 1 | 1 | 1 | 0 | 1 | **8** |
| Wade (2008) [[180](#_ENREF_180)] | 0 | 0 | -1 | -1 | -1 | -1 | -1 | -1 | -1 | **-7** |
| Wilhelmsen (2011) [[181](#_ENREF_181)] | -1 | -1 | -1 | -1 | -1 | -1 | -1 | -1 | -1 | **-9** |
| Wilkinson (2016) [[182](#_ENREF_182)] | 1 | 1 | 1 | 1 | 1 | 1 | 1 | 1 | 1 | **9** |
| Winkler (2014) [[183](#_ENREF_183)] | 1 | 1 | 1 | 1 | 1 | 1 | 1 | 1 | 1 | **9** |
| Witt-Enderby (2006) [[184](#_ENREF_184)] | -1 | -1 | -1 | -1 | -1 | -1 | -1 | -1 | -1 | **-9** |
| Wright (2015) [[185](#_ENREF_185)] | 1 | 1 | 0 | 1 | 1 | 1 | 1 | 1 | 1 | **8** |
| Xin (2009) [[186](#_ENREF_186)] | -1 | -1 | -1 | -1 | -1 | -1 | -1 | -1 | -1 | **-9** |
| Xin (2015) [[187](#_ENREF_187)] | -1 | -1 | -1 | -1 | -1 | -1 | -1 | -1 | -1 | **-9** |
| Xu (2015) [[188](#_ENREF_188)] | 1 | 1 | 1 | 1 | 1 | 1 | 1 | 0 | 1 | **8** |
| Yang (2014) [[189](#_ENREF_189)] | -1 | -1 | -1 | -1 | -1 | -1 | -1 | -1 | -1 | **-9** |
| Yang (2014) [[190](#_ENREF_190)] | 1 | 1 | 1 | 1 | 1 | 0 | 1 | 1 | 1 | **8** |
| Yang (2016) [[191](#_ENREF_191)] | 1 | 1 | 1 | 1 | 1 | 1 | 1 | 1 | 1 | **9** |
| Yousaf (2010) [[192](#_ENREF_192)] | 1 | 1 | 1 | 1 | 1 | 1 | 1 | -1 | 1 | **7** |
| Zetner (2016) [[193](#_ENREF_193)] | 1 | 1 | 1 | 1 | -1 | -1 | -1 | -1 | 1 | **1** |
| Zhang (2016) [[194](#_ENREF_194)] | 1 | 1 | 0 | 0 | 0 | 0 | 1 | 1 | 0 | **4** |
| Zhang (2017) [[195](#_ENREF_195)] | -1 | -1 | -1 | -1 | -1 | -1 | -1 | -1 | -1 | **-9** |

**Table 7** Footnote: SR- systematic review; MLT- melatonin

Scoring: Each Question is Scored as **1**, **0**, or **-1**.

**1** means that: (a) the review states the databases used, date of most recent searches, and some mention of search terms; (b) the review searches at least 2 databases and looks at other sources; (c) the review states the criteria used for deciding which studies to include in the overview; (d) the review reports how many studies were identified by searches, numbers excluded, and appropriate reasons for excluding them; (e) the review states the criteria used for assessing the validity of the included studies; (f) the review reports validity assessment and did some type of analysis with it; (g) the report mentions that quantitative analysis was not possible and reasons that it could not be done; (h) the review performs a test for heterogeneity before pooling or does appropriate subgroup testing, appropriate sensitivity analysis, or other such analysis; (i) the conclusions made by the author(s) are supported by the data and/or analysis reported in the review.

**0** means that the above mentioned criteria were partially fulfilled.

**-1** means that none of the above criteria were fulfilled.

***** Operationalisation of the Oxman criteria [[196](#_ENREF_196)]

**References**

1. Agorastos A, Linthorst ACE: Potential pleiotropic beneficial effects of adjuvant melatonergic treatment in posttraumatic stress disorder. *Journal of Pineal Research: Molecular, Biological, Physiological and Clinical Aspects of Melatonin* 2016, 61(1):3-26.

2. Andersen LP, Werner MU, Rosenberg J, Gogenur I: A systematic review of peri-operative melatonin. *Anaesthesia* 2014, 69(10):1163-1171.

3. Anderson G, Maes M: Melatonin: an overlooked factor in schizophrenia and in the inhibition of anti-psychotic side effects. *Metab Brain Dis* 2012, 27(2):113-119.

4. Aranda ML, Fleitas MF, Dieguez H, Iaquinandi A, Sande PH, Dorfman D, Rosenstein RE: Melatonin As A Therapeutic Resource For Inflammatory Visual Diseases. *Current Neuropharmacology* 2017, 13:13.

5. Arendt J: Importance and Relevance of Melatonin to Human Biological Rhythms. *J Neuroendocrinol* 2003, 15(4):427-431.

6. Armour D, Paton C: Melatonin in the treatment of insomnia in children and adolescents. *Psychiatric Bulletin* 2004, 28(6):222-224.

7. Arora H, Ivanovski S: Melatonin as a pro-osteogenic agent in oral implantology: a systematic review of histomorphometric outcomes in animals and quality evaluation using ARRIVE guidelines. *J Periodontal Res*.

8. Atkinson G, Drust B, Reilly T, Waterhouse J: The Relevance of Melatonin to Sports Medicine and Science. *Sports Med* 2003, 33(11):809-831.

9. Aversa S, Pellegrino S, Barberi I, Reiter RJ, Gitto E: Potential utility of melatonin as an antioxidant during pregnancy and in the perinatal period. *J Matern Fetal Neonatal Med* 2012, 25(3):207-221.

10. Bartsch C, Bartsch H: Melatonin in cancer patients and in tumor-bearing animals. *Adv Exp Med Biol* 1999, 467:247-264.

11. Bartsch C, Bartsch H: The anti-tumor activity of pineal melatonin and cancer enhancing life styles in industrialized societies. *Cancer Causes Control* 2006, 17(4):559-571.

12. Barron ML: Light exposure, melatonin secretion, and menstrual cycle parameters: An integrative review. *Biol Res Nurs* 2007, 9(1):49-69.

13. Basler M, Jetter A, Fink D, Seifert B, Kullak-Ublick GA, Trojan A: Urinary excretion of melatonin and association with breast cancer: meta-analysis and review of the literature. *Breast Care (Basel)* 2014, 9(3):182-187.

14. Bellon AM: Searching for New Options for Treating Insomnia: Are Melatonin and Ramelteon Beneficial? [Article]. *J Psychiatr Pract* 2006, 12(4):229-243.

15. Bendz LM, Scates AC: Melatonin treatment for insomnia in pediatric patients with attention-deficit/hyperactivity disorder. *Ann Pharmacother* 2010, 44(1):185-191.

16. Benitez-King G, Soto-Vega E, Ramirez-Rodriguez G: Melatonin modulates microfilament phenotypes in epithelial cells: implications for adhesion and inhibition of cancer cell migration. *Histol Histopathol* 2009, 24(6):789-799.

17. Beyer CE, Steketee JD, Saphier D: Antioxidant properties of melatonin - An emerging mystery. *Biochem Pharmacol* 1998, 56(10):1265-1272.

18. Biran V, Phan Duy A, Decobert F, Bednarek N, Alberti C, Baud O: Is melatonin ready to be used in preterm infants as a neuroprotectant? *Dev Med Child Neurol* 2014, 56(8):717-723.

19. Bizzarri M, Proietti S, Cucina A, Reiter RJ: Molecular mechanisms of the pro-apoptotic actions of melatonin in cancer: a review. *Expert Opin Ther Targets* 2013, 17(12):1483-1496.

20. Blask DE, Hill SM, Dauchy RT, Xiang S, Yuan L, Duplessis T, Mao L, Dauchy E, Sauer LA: Circadian regulation of molecular, dietary, and metabolic signaling mechanisms of human breast cancer growth by the nocturnal melatonin signal and the consequences of its disruption by light at night. *J Pineal Res* 2011, 51(3):259-269.

21. Boga JA, Coto-Montes A, Rosales-Corral SA, Tan D-X, Reiter RJ: Beneficial actions of melatonin in the management of viral infections: a new use for this "molecular handyman"? *Rev Med Virol* 2012, 22(5):323-338.

22. Bonmati-Carrion MA, Arguelles-Prieto R, Martinez-Madrid MJ, Reiter R, Hardeland R, Rol MA, Madrid JA: Protecting the melatonin rhythm through circadian healthy light exposure. *Int J Mol Sci* 2014, 15(12):23448-23500.

23. Bonnefont-Rousselot D, Collin F: Melatonin: Action as antioxidant and potential applications in human disease and aging. *Toxicology* 2010, 278(1):55-67.

24. Braam W, Smits MG, Didden R, Korzilius H, Van Geijlswijk IM, Curfs LM: Exogenous melatonin for sleep problems in individuals with intellectual disability: a meta-analysis. *Dev Med Child Neurol* 2009, 51(5):340-349.

25. Brigo F, Igwe SC: Melatonin as add-on treatment for epilepsy. *Cochrane Database Syst Rev* 2016, 2016(3).

26. Brzezinski A: 'Melatonin replacement therapy' for postmenopausal women: Is it justified? *Menopause* 1998, 5(1):60-64.

27. Brzezinski A, Vangel MG, Wurtman RJ, Norrie G, Zhdanova I, Ben-Shushan A, Ford I: Effects of exogenous melatonin on sleep: A meta-analysis. *Sleep Med Rev* 2005, 9(1):41-50.

28. Bubenik GA, Blask DE, Brown GM, Maestroni GJ, Pang SF, Reiter RJ, Viswanathan M, Zisapel N: Prospects of the clinical utilization of melatonin. *Biol Signals Recept* 1998, 7(4):195-219.

29. Buscemi NP, Vandermeer BM, Hooton NB, Pandya RMPH, Tjosvold LM, Hartling LM, Baker GPD, Klassen TPMDM, Vohra SMDM: The Efficacy and Safety of Exogenous Melatonin for Primary Sleep Disorders: A Meta-Analysis. *J Gen Intern Med* 2005, 20(12):1151-1158.

30. Buscemi Nra, Vandermeer Bs, Hooton Npc, Pandya Rpm, Tjosvold Lrl, Hartling Lad, Vohra Sd, Klassen TPd, Baker Gp, chair: Efficacy and safety of exogenous melatonin for secondary sleep disorders and sleep disorders accompanying sleep restriction: meta-analysis. *BMJ* 2006, 332(7538):385-393.

31. Cardinali DP, Golombek DA, Rosenstein RE, Brusco LI, Vigo DE: Assessing the efficacy of melatonin to curtail benzodiazepine/Z drug abuse. *Pharmacol Res* 2015.

32. Carlomagno G, Nordio M, Chiu TT, Unfer V: Contribution of myo-inositol and melatonin to human reproduction. *Eur J Obstet Gynecol Reprod Biol* 2011, 159(2):267-272.

33. Carpentieri A, Diaz de Barboza G, Areco V, Peralta Lopez M, Tolosa de Talamoni N: New perspectives in melatonin uses. *Pharmacol Res* 2012, 65(4):437-444.

34. Carrillo-Vico A, Guerrero JM, Lardone PJ, Reiter RJ: A Review of the Multiple Actions of Melatonin on the Immune System. *Endocrine* 2005, 27(2):189-200.

35. Cervantes M, Morali G, Letechipia-Vallejo G: Melatonin and ischemia-reperfusion injury of the brain. *J Pineal Res* 2008, 45(1):1-7.

36. Chaplin SMM, Nutt DDMFFF: Melatonin (Circadin): a novel hypnotic for use in older patients. *Prescriber* 2008, 19(20):21-24.

37. Chen CQ, Fichna J, Bashashati M, Li YY, Storr M: Distribution, function and physiological role of melatonin in the lower gut. *World J Gastroenterol* 2011, 17(34):3888-3898.

38. Chen YC, Tain YL, Sheen JM, Huang LT: Melatonin utility in neonates and children. *J Formos Med Assoc* 2012, 111(2):57-66.

39. Chen S, Shi L, Liang F, Xu L, Desislava D, Wu Q, Zhang J: Exogenous Melatonin for Delirium Prevention: a Meta-analysis of Randomized Controlled Trials. *Mol Neurobiol* 2015.

40. Cheung RT: The utility of melatonin in reducing cerebral damage resulting from ischemia and reperfusion. *J Pineal Res* 2003, 34(3):153-160.

41. Cos S, Sanchez-Barcelo EJ: Melatonin, experimental basis for a possible application in breast cancer prevention and treatment. *Histol Histopathol* 2000, 15(2):637-647.

42. Costello RB, Lentino CV, Boyd CC, O'Connell ML, Crawford CC, Sprengel ML, Deuster PA: The effectiveness of melatonin for promoting healthy sleep: A rapid evidence assessment of the literature. *Nutrition Journal* 2014:106.

43. Cutando A, Aneiros-Fernandez J, Aneiros-Cachaza J, Arias-Santiago S: Melatonin and cancer: current knowledge and its application to oral cavity tumours. *J Oral Pathol Med* 2011, 40(8):593-597.

44. Cutando A, Lopez-Valverde A, J DEV, Gimenez JL, Carcia IA, RG DED: Action of melatonin on squamous cell carcinoma and other tumors of the oral cavity (Review). *Oncol Lett* 2014, 7(4):923-926.

45. De Crescenzo F, Lennox A, Gibson JC, Cordey JH, Stockton S, Cowen PJ, Quested DJ: Melatonin as a treatment for mood disorders: A systematic review. *Acta Psychiatrica Scandinavica* 2017.

46. De Jonghe A, Korevaar JC, Van Munster BC, De Rooij SE: Effectiveness of melatonin treatment on circadian rhythm disturbances in dementia. Are there implications for delirium? A systematic review. *Int J Geriatr Psychiatry* 2010, 25(12):1201-1208.

47. De Rooij SE, Van Munster BC: Melatonin deficiency hypothesis in delirium: A synthesis of current evidence. *Rejuvenation Res* 2013, 16(4):273-278.

48. Di Bella L, Gualano L: Key aspects of melatonin physiology: thirty years of research. *Neuro Endocrinol Lett* 2006, 27(4):425-432.

49. Dopfel RP, Schulmeister K, Schernhammer ES: Nutritional and lifestyle correlates of the cancer-protective hormone melatonin. *Cancer Detect Prev* 2007, 31(2):140-148.

50. Dragojevic Dikic S, Jovanovic AM, Dikic S, Jovanovic T, Jurisic A, Dobrosavljevic A: Melatonin: a "Higgs boson" in human reproduction. *Gynecol Endocrinol* 2015, 31(2):92-101.

51. Dziegiel P, Podhorska-Okolow M, Zabel M: Melatonin: adjuvant therapy of malignant tumors. *Med Sci Monit* 2008, 14(5):Ra64-70.

52. Elmahallawy EK, Luque JO, Aloweidi AS, Gutiérrez-Fernández J, Sampedro-Martínez A, Rodriguez-Granger J, Kaki A, Agil A: Potential relevance of melatonin against some infectious agents: A review and assessment of recent research. *Curr Med Chem* 2015, 22(33):3848-3861.

53. Erdemli HK, Akyol S, Armutcu F, Gulec MA, Canbal M, Akyol O: Melatonin and caffeic acid phenethyl ester in the regulation of mitochondrial function and apoptosis: The basis for future medical approaches. *Life Sci* 2016, 148 Supplement(C):305-312.

54. Escames G, Acuna-Castroviejo D, Lopez LC, Tan D-x, Maldonado MD, Sanchez-Hidalgo M, Leon J, Reiter RJ: Pharmacological utility of melatonin in the treatment of septic shock: experimental and clinical evidence. *J Pharm Pharmacol* 2006, 58(9):1153-1165.

55. Escames G, Ozturk G, Bano-Otalora B, Pozo MJ, Madrid JA, Reiter RJ, Serrano E, Concepcion M, Acuna-Castroviejo D: Exercise and melatonin in humans: reciprocal benefits. *J Pineal Res* 2012, 52(1):1-11.

56. Favero G, Rodella LF, Reiter RJ, Rezzani R: Melatonin and its atheroprotective effects: a review. *Mol Cell Endocrinol* 2014, 382(2):926-937.

57. Fernando S, Rombauts L: Melatonin: shedding light on infertility?--A review of the recent literature. *J Ovarian Res* 2014, 7:98.

58. Ferracioli-Oda E, Qawasmi A, Bloch MH: Meta-analysis: melatonin for the treatment of primary sleep disorders. *PLoS One* 2013, 8(5):e63773.

59. Fildes JE, Yonan N, Keevil BG: Melatonin--a pleiotropic molecule involved in pathophysiological processes following organ transplantation. *Immunology* 2009, 127(4):443-449.

60. Giannoulia-Karantana A, Vlachou A, Polychronopoulou S, Papassotiriou I, Chrousos GP: Melatonin and immunomodulation: connections and potential clinical applications. *Neuroimmunomodulation* 2006, 13(3):133-144.

61. Golombek DAa, Pandi-Perumal SRb, Brown GMc, Cardinali DPd: Some implications of melatonin use in chronopharmacology of insomnia. *Eur J Pharmacol* 2015, 762 Supplement(C):42-48.

62. Gomez-Moreno G, Guardia J, Ferrera MJ, Cutando A, Reiter RJ: Melatonin in diseases of the oral cavity. *Oral Dis* 2010, 16(3):242-247.

63. Govender J, Loos B, Marais E, Engelbrecht AM: Mitochondrial catastrophe during doxorubicin-induced cardiotoxicity: a review of the protective role of melatonin. *J Pineal Res* 2014, 57(4):367-380.

64. Guaiana G, Gupta S, Chiodo D, Davies SJ, Haederle K, Koesters M: Agomelatine versus other antidepressive agents for major depression. *Cochrane Database Syst Rev* 2013(12):Cd008851.

65. Guenole F, Godbout R, Nicolas A, Franco P, Claustrat B, Baleyte JM: Melatonin for disordered sleep in individuals with autism spectrum disorders: systematic review and discussion. *Sleep Med Rev* 2011, 15(6):379-387.

66. Grant SG, Melan MA, Latimer JJ, Witt-Enderby PA: Melatonin and breast cancer: cellular mechanisms, clinical studies and future perspectives. *Expert Rev Mol Med* 2009, 11:e5.

67. Grossman E, Laudon M, Zisapel N: Effect of melatonin on nocturnal blood pressure: meta-analysis of randomized controlled trials. *Vasc Health Risk Manag* 2011, 7:577-584.

68. Hansen MV, Danielsen AK, Hageman I, Rosenberg J, Gogenur I: The therapeutic or prophylactic effect of exogenous melatonin against depression and depressive symptoms: a systematic review and meta-analysis. *Eur Neuropsychopharmacol* 2014, 24(11):1719-1728.

69. Hansen MV, Halladin NL, Rosenberg J, Gogenur I, Moller MA: Melatonin for pre- and postoperative anxiety in adults. *Cochrane Database Syst Rev* 2015(4).

70. Hardeland Ra, Cardinali DPb, Brown GMc, Pandi-Perumal SRd: Melatonin and brain inflammaging. *Prog Neurobiol* 2015, 127-128 Suppl.(C):46-63.

71. Harrod CG, Bendok BR, Batjer HH: Interactions between melatonin and estrogen may regulate cerebrovascular function in women: Clinical implications for the effective use of HRT during menopause and aging. *Med Hypotheses* 2005, 64(4):725-735.

72. Hartley S, Quera-Salva M-A: Implication of Circadian Rhythms and Melatonin in Major Depressive Disorder: The Evidence Base for New Antidepressant Treatment. *Curr Psychiatry Rev* 2014, 10(3):223-234.

73. Heiligenstein E, Guenther G: Over-the-counter psychotropics: a review of melatonin, St John's wort, valerian, and kava-kava. *J Am Coll Health* 1998, 46(6):271-276.

74. Herxheimer A, Petrie KJ: Melatonin for the prevention and treatment of jet lag. *Cochrane Database Syst Rev* 2002(2):CD001520.

75. Hill SM, Belancio VP, Dauchy RT, Xiang S, Brimer S, Mao L, Hauch A, Lundberg PW, Summers W, Yuan L *et al*: Melatonin: an inhibitor of breast cancer. *Endocr Relat Cancer* 2015, 22(3):R183-204.

76. Hong Y, Palaksha KJ, Park K, Park S, Kim H-D, Reiter RJ, Chang K-T: Melatonin plus exercise-based neurorehabilitative therapy for spinal cord injury. *J Pineal Res* 2010, 49(3):201-209.

77. Hosseinzadeh A, Kamrava SK, Joghataei MT, Darabi R, Shakeri‐Zadeh A, Shahriari M, Reiter RJ, Ghaznavi H, Mehrzadi S: Apoptosis signaling pathways in osteoarthritis and possible protective role of melatonin. *J Pineal Res* 2016, 61(4):411-425.

78. Hrenak J, Paulis L, Repova K, Aziriova S, Nagtegaal EJ, Reiter RJ, Simko F: Melatonin and renal protection: Novel perspectives from animal experiments and human studies (review). *Curr Pharm Des* 2015, 21(7):936-949.

79. Hu W, Ma Z, Jiang S, Fan C, Deng C, Yan X, Di S, Lv J, Reiter RJ, Yang Y: Melatonin: The dawning of a treatment for fibrosis? *Journal of Pineal Research: Molecular, Biological, Physiological and Clinical Aspects of Melatonin* 2016, 60(2):121-131.

80. Huang KL, Lu WC, Wang YY, Hu GC, Lu CH, Lee WY, Hsu CC: Comparison of agomelatine and selective serotonin reuptake inhibitors/serotonin-norepinephrine reuptake inhibitors in major depressive disorder: A meta-analysis of head-to-head randomized clinical trials. *Aust N Z J Psychiatry* 2014, 48(7):663-671.

81. Hunter CM, Figueiro MG: Measuring Light at Night and Melatonin Levels in Shift Workers: A Review of the Literature. *Biological Research for Nursing* 2017, 19(4):365-374.

82. Jan JE, Wasdell MB, Reiter RJ, Weiss MD, Johnson KP, Ivanenko A, Freeman RD: Melatonin therapy of pediatric sleep disorders: Recent advances, why it works, who are the candidates and how to treat. *Curr Pediatric Rev* 2007, 3(3):214-224.

83. Jansen SL, Forbes DA, Duncan V, Morgan DG: Melatonin for cognitive impairment. *Cochrane Database Syst Rev* 2006(1).

84. Jemima J, Bhattacharjee P, Singhal RS: Melatonin - a review on the lesser known potential nutraceutical. *Int J Pharm Sci Res* 2011, 2(8):1975-1987.

85. Jena GMMP, Trivedi PPMS: A Review of the Use of Melatonin in Ulcerative Colitis: Experimental Evidence and New Approaches. *Inflamm Bowel Dis* 2014, 20(3):553-563.

86. Jung B, Ahmad N: Melatonin in cancer management: progress and promise. *Cancer Res* 2006, 66(20):9789-9793.

87. Jung-Hynes B, Reiter RJ, Ahmad N: Sirtuins, melatonin and circadian rhythms: building a bridge between aging and cancer. *J Pineal Res* 2010, 48(1):9-19.

88. KaminskiHartenthaler A, Nussbaumer B, Forneris CA, Morgan LC, Gaynes BN, Sonis JH, Greenblatt A, Wipplinger J, Lux LJ, Winkler D *et al*: Melatonin and agomelatine for preventing seasonal affective disorder. *Cochrane Database Syst Rev* 2015(11).

89. Karaaslan C, Suzen S: Antioxidant properties of melatonin and its potential action in diseases. *Curr Top Med Chem* 2015, 15(9):894-903.

90. Keegan LJ, Reed-Berendt R, Neilly E, Morrall MC, Murdoch-Eaton D: Effectiveness of melatonin for sleep impairment post paediatric acquired brain injury: evidence from a systematic review. *Dev Neurorehabil* 2014, 17(5):355-362.

91. Kennaway DJ: Potential safety issues in the use of the hormone melatonin in paediatrics. *J Paediatr Child Health* 2015, 51(6):584-589.

92. Kuriyama A, Honda M, Hayashino Y: Ramelteon for the treatment of insomnia in adults: a systematic review and meta-analysis. *Sleep Med* 2014, 15(4):385-392.

93. Lanfumey L, Mongeau R, Hamon M: Biological rhythms and melatonin in mood disorders and their treatments. *Pharmacol Ther* 2013, 138(2):176-184.

94. Lee MS, Yin T-C, Sung P-H, Chiang JY, Sun C-K, Yip H-K: Melatonin enhances survival and preserves functional integrity of stem cells: A review. *Journal of Pineal Research* 2017, 62(2):n/a-n/a.

95. Leger D, Quera-Salva MA, Vecchierini MF, Ogrizek P, Perry CA, Dressman MA: Safety profile of tasimelteon, a melatonin MT1 and MT2 receptor agonist: pooled safety analyses from six clinical studies. *Expert Opin Drug Saf* 2015, 14(11):1673-1685.

96. Lemoine P, Zisapel N: Prolonged-release formulation of melatonin (Circadin) for the treatment of insomnia. *Expert Opin Pharmacother* 2012, 13(6):895-905.

97. Leone M, Bussone G: Melatonin in cluster headache: Rationale for use and possible therapeutic potential. *CNS Drugs* 1998, 9(1):7-16.

98. Liira J, Verbeek JH, Costa G, Driscoll TR, Sallinen M, Isotalo LK, Ruotsalainen JH: Pharmacological interventions for sleepiness and sleep disturbances caused by shift work. *Cochrane Database Syst Rev* 2014(8):Cd009776.

99. Liu J, Wang Ln: Ramelteon in the treatment of chronic insomnia: systematic review and meta-analysis. *Int J Clin Pract* 2012, 66(9):867-873.

100. Ma Z, Yang Y, Fan C, Han J, Wang D, Di S, Hu W, Liu D, Li X, Reiter RJ *et al*: Melatonin as a potential anticarcinogen for non-small-cell lung cancer. *Oncotarget* 2016.

101. Macleod MR, O'Collins T, Horky LL, Howells DW, Donnan GA: Systematic review and meta-analysis of the efficacy of melatonin in experimental stroke. *J Pineal Res* 2005, 38(1):35-41.

102. Maldonado M-DMDP, Murillo-Cabezas FMDP, Calvo J-RMDP, Lardone P-JM, Tan D-XMDP, Guerrero J-MMDP, Reiter RJMDP: Melatonin as pharmacologic support in burn patients: A proposed solution to thermal injury-related lymphocytopenia and oxidative damage. *Crit Care Med* 2007, 35(4):1177-1185.

103. Maldonado MD, Reiter RJ, Perez-San-Gregorio MA: Melatonin as a potential therapeutic agent in psychiatric illness. *Hum Psychopharmacol* 2009, 24(5):391-400.

104. Malhotra S, Sawhney G, Pandhi P: The therapeutic potential of melatonin: a review of the science. *MedGenMed* 2004, 6(2):46.

105. Maria S, Witt-Enderby PA: Melatonin effects on bone: Potential use for the prevention and treatment for osteopenia, osteoporosis, and periodontal disease and for use in bone-grafting procedures. *J Pineal Res* 2014, 56(2):115-125.

106. Marrin K, Drust B, Gregson W, Atkinson G: A meta-analytic approach to quantify the dose-response relationship between melatonin and core temperature. *Eur J Appl Physiol* 2013, 113(9):2323-2329.

107. Marseglia L, D'Angelo G, Barberi I, Manti S, Salpietro C, Arrigo T, Reiter RJ, Gitto E: Melatonin and atopy: Role in atopic dermatitis and asthma. *Int J Mol Sci* 2014, 15(8):13482-13493.

108. Marseglia L, D'Angelo G, Manti S, Reiter RJ, Gitto E: Potential Utility of Melatonin in Preeclampsia, Intrauterine Fetal Growth Retardation, and Perinatal Asphyxia. *Reproductive Sciences* 2016, 23(8):970-977.

109. Mayo JC, Sainz RM, González Menéndez P, Cepas V, Tan DX, Reiter RJ: Melatonin and sirtuins: A 'not‐so unexpected' relationship. *Journal of Pineal Research: Molecular, Biological, Physiological and Clinical Aspects of Melatonin* 2017, 62(2):1-17.

110. Mehta A, Kaur G: Potential role of melatonin in prevention and treatment of oral carcinoma. *Indian J Dent* 2014, 5(2):86-91.

111. McGrane IR, Leung JG, St Louis EK, Boeve BF: Melatonin therapy for REM sleep behavior disorder: a critical review of evidence. *Sleep Med* 2015, 16(1):19-26.

112. Mihara T, Nakamura N, Ka K, Oba MS, Goto T: Effects of melatonin premedication to prevent emergence agitation after general anaesthesia in children: A systematic review and meta-analysis with trial sequential analysis. *Eur J Anaesthesiol* 2015, 32(12):862-871.

113. Mills E, Wu P, Seely D, Guyatt G: Melatonin in the treatment of cancer: A systematic review of randomized controlled trials and meta-analysis. *J Pineal Res* 2005, 39(4):360-366.

114. Miroddi M, Bruno R, Galletti F, Calapai F, Navarra M, Gangemi S, Calapai G: Clinical pharmacology of melatonin in the treatment of tinnitus: a review. *Eur J Clin Pharmacol* 2015, 71(3):263-270.

115. Mozaffari S, Rahimi R, Abdollahi M: Implications of melatonin therapy in irritable bowel syndrome: a systematic review. *Curr Pharm Des* 2010, 16(33):3646-3655.

116. Najeeb S, Khurshid Z, Zohaib S, Zafar MS: Therapeutic potential of melatonin in oral medicine and periodontology. *Kaohsiung Journal of Medical Sciences* 2016, 32(8):391-396.

117. Nduhirabandi F, du Toit EF, Lochner A: Melatonin and the metabolic syndrome: a tool for effective therapy in obesity-associated abnormalities? *Acta Physiologica* 2012, 205(2):209-223.

118. Melatonin for sleep problems in children with neurodevelopmental disorders. *Drug & Therapeutics Bulletin* 2015, 53(10):117-120.

119. Nowak JZ, Zawilska JB: Melatonin and its physiological and therapeutic properties. *Pharm World Sci* 1998, 20(1):18-27.

120. Olde Rikkert MG, Rigaud AS: Melatonin in elderly patients with insomnia. A systematic review. *Z Gerontol Geriatr* 2001, 34(6):491-497.

121. Pacchierotti C, Iapichino S, Bossini L, Pieraccini F, Castrogiovanni P: Melatonin in psychiatric disorders: a review on the melatonin involvement in psychiatry. *Front Neuroendocrinol* 2001, 22(1):18-32.

122. Pandi-Perumal SR, Trakht I, Spence DW, Srinivasan V, Dagan Y, Cardinali DP: The roles of melatonin and light in the pathophysiology and treatment of circadian rhythm sleep disorders. *Nat Clin Pract Neurol* 2008, 4(8):436-447.

123. Pandi-Perumal SR, Trakht I, Srinivasan V, Spence DW, Maestroni GJ, Zisapel N, Cardinali DP: Physiological effects of melatonin: role of melatonin receptors and signal transduction pathways. *Prog Neurobiol* 2008, 85(3):335-353.

124. Panzer A, Viljoen M: The validity of melatonin as an oncostatic agent. *J Pineal Res* 1997, 22(4):184-202.

125. Paul R, Borah A: The potential physiological crosstalk and interrelationship between two sovereign endogenous amines, melatonin and homocysteine. *Life Sci* 2015, 139:97-107.

126. Phillips L, Appleton RE: Systematic review of melatonin treatment in children with neurodevelopmental disabilities and sleep impairment. *Dev Med Child Neurol* 2004, 46(11):771-775.

127. Pytka K, Mlyniec K, Podkowa K, Podkowa A, Jakubczyk M, Zmudzka E, Lustyk K, Sapa J, Filipek B: The role of melatonin, neurokinin, neurotrophic tyrosine kinase and glucocorticoid receptors in antidepressant-like effect. *Pharmacological Reports* 2017, 69(3):546-554.

128. Ramis MR, Esteban S, Miralles A, Tan DX, Reiter RJ: Protective Effects of Melatonin and Mitochondria-targeted Antioxidants Against Oxidative Stress: A Review. *Curr Med Chem* 2015, 22(22):2690-2711.

129. Ramos E, Patino P, Reiter RJ, Gil-Martin E, Marco-Contelles J, Parada E, los Rios CD, Romero A, Egea J: Ischemic brain injury: New insights on the protective role of melatonin. *Free Radical Biology and Medicine* 2017, 104:32-53.

130. Reiter RJ, Tan DX, Qi W, Manchester LC, Karbownik M, Calvo JR: Pharmacology and physiology of melatonin in the reduction of oxidative stress in vivo. *Biol Signals Recept* 2000, 9(3-4):160-171.

131. Reiter RJ, Acuna-Castroviejo D, Tan DX, Burkhardt S: Free radical-mediated molecular damage. Mechanisms for the protective actions of melatonin in the central nervous system. *Ann N Y Acad Sci* 2001, 939:200-215.

132. Reiter RJ, Tan DX, Manchester LC, Qi W: Biochemical reactivity of melatonin with reactive oxygen and nitrogen species: a review of the evidence. *Cell Biochem Biophys* 2001, 34(2):237-256.

133. Reiter RJ: Melatonin: clinical relevance. *Best Pract Res Clin Endocrinol Metab* 2003, 17(2):273-285.

134. Reiter RJ, Sainz RM, Lopez-Burillo S, Mayo JC, Manchester LC, Tan DX: Melatonin Ameliorates Neurologic Damage and Neurophysiologic Deficits in Experimental Models of Stroke. *Ann N Y Acad Sci* 2003, 993:35-47.

135. Reiter RJ, Tan D-X, Pappolla MA: Melatonin Relieves the Neural Oxidative Burden that Contributes to Dementias. *Ann N Y Acad Sci* 2004:179-196.

136. Reiter RJ, Tan DX, Leon J, Kilic U, Kilic E: When melatonin gets on your nerves: its beneficial actions in experimental models of stroke. *Exp Biol Med (Maywood)* 2005, 230(2):104-117.

137. Reiter RJ, Tan DX, Korkmaz A, Erren TC, Piekarski C, Tamura H, Manchester LC: Light at night, chronodisruption, melatonin suppression, and cancer risk: a review. *Crit Rev Oncog* 2007, 13(4):303-328.

138. Korkmaz A, Sanchez-Barcelo EJ, Tan DX, Reiter RJ: Role of melatonin in the epigenetic regulation of breast cancer. *Breast Cancer Res Treat* 2009, 115(1):13-27.

139. Reiter RJ, Tan DX, Korkmaz A, Ma S: Obesity and metabolic syndrome: Association with chronodisruption, sleep deprivation, and melatonin suppression. *Ann Med* 2012, 44(6):564-577.

140. Reiter RJ, Tan DX, Tamura H, Cruz MH, Fuentes-Broto L: Clinical relevance of melatonin in ovarian and placental physiology: a review. *Gynecol Endocrinol* 2014, 30(2):83-89.

141. Reiter RJ, Rosales-Corral SA, Manchester LC, Liu X, Tan DX: Melatonin in the biliary tract and liver: Health implications. *Curr Pharm Des* 2014, 20(30):4788-4801.

142. Rodríguez AD: Melatonin in the acute coronary syndromes. *Salud(i)Ciencia* 2007, 15(6):983-985.

143. Romero A, Ramos E, de Los Rios C, Egea J, Del Pino J, Reiter RJ: A review of metal-catalyzed molecular damage: protection by melatonin. *J Pineal Res* 2014, 56(4):343-370.

144. Rondanelli M, Faliva MA, Perna S, Antoniello N: Update on the role of melatonin in the prevention of cancer tumorigenesis and in the management of cancer correlates, such as sleep-wake and mood disturbances: Review and remarks. *Aging Clin Exp Res* 2013, 25(5):499-510.

145. Rossignol DA, Frye RE: Melatonin in autism spectrum disorders: a systematic review and meta-analysis. *Dev Med Child Neurol* 2011, 53(9):783-792.

146. Ryung Wang H, Sup Woo Y, Bahk WM: The role of melatonin and melatonin agonists in counteracting antipsychotic-induced metabolic side effects: a systematic review. *Int Clin Psychopharmacol* 2016.

147. Sajith SG, Clarke D: Melatonin and sleep disorders associated with intellectual disability: a clinical review. *J Intellect Disabil Res* 2007, 51(Pt 1):2-13.

148. Samantaray S, Das A, Thakore NP, Matzelle DD, Reiter RJ, Ray SK, Banik NL: Therapeutic potential of melatonin in traumatic central nervous system injury. *J Pineal Res* 2009, 47(2):134-142.

149. Sanchez-Barcelo EJ, Cos S, Mediavilla D, Martinez-Campa C, Gonzalez A, Alonso-Gonzalez C: Melatonin-estrogen interactions in breast cancer. *J Pineal Res* 2005, 38(4):217-222.

150. Sanchez-Barcelo EJ, Mediavilla MD, Tan DX, Reiter RJ: Clinical uses of melatonin: evaluation of human trials. *Curr Med Chem* 2010, 17(19):2070-2095.

151. Sanchez-Barcelo EJ, Mediavilla MD, Alonso-Gonzalez C, Reiter RJ: Melatonin uses in oncology: breast cancer prevention and reduction of the side effects of chemotherapy and radiation. *Expert Opin Investig Drugs* 2012, 21(6):819-831.

152. Scholtens RM, van Munster BC, van Kempen MF, de Rooij SEJA: Physiological melatonin levels in healthy older people: A systematic review. *J Psychosom Res* 2016, 86:20-27.

153. Seely D, Wu P, Fritz H, Kennedy DA, Tsui T, Seely AJ, Mills E: Melatonin as adjuvant cancer care with and without chemotherapy: a systematic review and meta-analysis of randomized trials. *Integr Cancer Ther* 2012, 11(4):293-303.

154. Seko LM, Moroni RM, Leitao VM, Teixeira DM, Nastri CO, Martins WP: Melatonin supplementation during controlled ovarian stimulation for women undergoing assisted reproductive technology: systematic review and meta-analysis of randomized controlled trials. *Fertil Steril* 2014, 101(1):154-161.e154.

155. Shirazi A, Ghobadi G, Ghazi-Khansari M: A radiobiological review on melatonin: a novel radioprotector. *J Radiat Res* 2007, 48(4):263-272.

156. Shiu SYW: Towards rational and evidence-based use of melatonin in prostate cancer prevention and treatment. *J Pineal Res* 2007, 43(1):1-9.

157. Singh M, Jadhav HR: Melatonin: functions and ligands. *Drug Discov Today* 2014, 19(9):1410-1418.

158. Srinivasan V, Spence WD, Pandi-Perumal SR, Zakharia R, Bhatnagar KP, Brzezinski A: Melatonin and human reproduction: Shedding light on the darkness hormone. *Gynecol Endocrinol* 2009, 25(12):779-785.

159. Srinivasan V, Lauterbach EC, Ho KY, Acuña-Castroviejo D, Zakaria R, Brzezinski A: Melatonin in antinociception: Its therapeutic applications. *Curr Neuropharmacol* 2012, 10(2):167-178.

160. Srinivasan V, Mohamed M, Kato H: Melatonin in bacterial and viral infections with focus on sepsis: a review. *Recent Pat Endocr Metab Immune Drug Discov* 2012, 6(1):30-39.

161. Su SC, Hsieh MJ, Yang WE, Chung WH, Reiter RJ, Yang SF: Cancer metastasis: Mechanisms of inhibition by melatonin. *Journal of Pineal Research: Molecular, Biological, Physiological and Clinical Aspects of Melatonin* 2017, 62(1):1-11.

162. Chang YS, Lin MH, Lee JH, Lee PL, Dai YS, Chu KH, Sun C, Lin YT, Wang LC, Yu HH *et al*: Melatonin Supplementation for Children with Atopic Dermatitis and Sleep Disturbance: A Randomized Clinical Trial. *JAMA Pediatrics* 2016, 170(1):35-42.

163. Tain YL, Huang LT, Hsu CN: Developmental Programming of Adult Disease: Reprogramming by Melatonin? *International Journal of Molecular Sciences* 2017, 18(2):16.

164. Tamura H, Nakamura Y, Terron MP, Flores LJ, Manchester LC, Tan DX, Sugino N, Reiter RJ: Melatonin and pregnancy in the human. *Reprod Toxicol* 2008, 25(3):291-303.

165. Tamura H, Nakamura Y, Korkmaz A, Manchester LC, Tan DX, Sugino N, Reiter RJ: Melatonin and the ovary: physiological and pathophysiological implications. *Fertil Steril* 2009, 92(1):328-343.

166. Tamura H, Takasaki A, Taketani T, Tanabe M, Lee L, Tamura I, Maekawa R, Aasada H, Yamagata Y, Sugino N: Melatonin and female reproduction. *J Obstet Gynaecol Res* 2014, 40(1):1-11.

167. Tan DX, Manchester LC, Fuentes-Broto L, Paredes SD, Reiter RJ: Significance and application of melatonin in the regulation of brown adipose tissue metabolism: Relation to human obesity. *Obes Rev* 2011, 12(3):167-188.

168. Terry PDPMPH, Villinger FDVMP, Bubenik GAMD, Sitaraman SVMDP: Melatonin and ulcerative colitis: Evidence, biological mechanisms, and future research. *Inflamm Bowel Dis* 2009, 15(1):134-140.

169. Tordjman S, Najjar I, Bellissant E, Anderson GM, Barburoth M, Cohen D, Jaafari N, Schischmanoff O, Fagard R, Lagdas E *et al*: Advances in the research of melatonin in autism spectrum disorders: literature review and new perspectives. *Int J Mol Sci* 2013, 14(10):20508-20542.

170. Turk J: Melatonin supplementation for severe and intractable sleep disturbance in young people with genetically determined developmental disabilities: short review and commentary. *J Med Genet* 2003, 40(11):793-796.

171. Valenzuela FJ, Vera J, Venegas C, Pino F, Lagunas C: Circadian System and Melatonin Hormone: Risk Factors for Complications during Pregnancy. *Obstet Gynecol Int* 2015, 2015:825802.

172. van Geijlswijk IM, Korzilius HP, Smits MG: The use of exogenous melatonin in delayed sleep phase disorder: a meta-analysis. *Sleep* 2010, 33(12):1605-1614.

173. Vijayalaxmi, Thomas CR, Jr., Reiter RJ, Herman TS: Melatonin: from basic research to cancer treatment clinics. *J Clin Oncol* 2002, 20(10):2575-2601.

174. Vijayalaxmi, Reiter RJ, Tan DX, Herman TS, Thomas CR, Jr.: Melatonin as a radioprotective agent: a review. *Int J Radiat Oncol Biol Phys* 2004, 59(3):639-653.

175. Vielma JR, Bonilla E, Chacin-Bonilla L, Mora M, Medina-Leendertz S, Bravo Y: Effects of melatonin on oxidative stress, and resistance to bacterial, parasitic, and viral infections: a review. *Acta Trop* 2014, 137:31-38.

176. Vural EMS, Van Munster BC, De Rooij SE: Optimal dosages for melatonin supplementation therapy in older adults: A systematic review of current literature. *Drugs Aging* 2014, 31(6):441-451.

177. Wang-Weigand S, McCue M, Ogrinc F, Mini L: Effects of ramelteon 8 mg on objective sleep latency in adults with chronic insomnia on nights 1 and 2: pooled analysis. *Curr Med Res Opin* 2009, 25(5):1209-1213.

178. Wang YM, Jin BZ, Ai F, Duan CH, Lu YZ, Dong TF, Fu QL: The efficacy and safety of melatonin in concurrent chemotherapy or radiotherapy for solid tumors: a meta-analysis of randomized controlled trials. *Cancer Chemother Pharmacol* 2012, 69(5):1213-1220.

179. Wang HR, Woo YS, Bahk WM: The role of melatonin and melatonin agonists in counteracting antipsychotic-induced metabolic side effects: a systematic review. *International Clinical Psychopharmacology* 2016, 31(6):301-306.

180. Wade A, Downie S: Prolonged-release melatonin for the treatment of Insomnia in patients over 55 years. *Expert Opin Investigat Drugs* 2008, 17(10):1567-1572.

181. Wilhelmsen M, Amirian I, Reiter RJ, Rosenberg J, Gogenur I: Analgesic effects of melatonin: a review of current evidence from experimental and clinical studies. *J Pineal Res* 2011, 51(3):270-277.

182. Wilkinson D, Shepherd E, Wallace EM: Melatonin for women in pregnancy for neuroprotection of the fetus. *Cochrane Database Syst Rev* 2016(3).

183. Winkler A, Auer C, Doering BK, Rief W: Drug treatment of primary insomnia: A meta-analysis of polysomnographic randomized controlled trials. *CNS Drugs* 2014, 28(9):799-816.

184. Witt-Enderby PA, Radio NM, Doctor JS, Davis VL: Therapeutic treatments potentially mediated by melatonin receptors: potential clinical uses in the prevention of osteoporosis, cancer and as an adjuvant therapy. *J Pineal Res* 2006, 41(4):297-305.

185. Wright A, Diebold J, Otal J, Stoneman C, Wong J, Wallace C, Duffett M: The Effect of Melatonin on Benzodiazepine Discontinuation and Sleep Quality in Adults Attempting to Discontinue Benzodiazepines: A Systematic Review and Meta-Analysis. *Drugs Aging* 2015, 32(12):1009-1018.

186. Wang X: The Antiapoptotic Activity of Melatonin in Neurodegenerative Diseases. *CNS: Neurosci Ther* 2009, 15(4):345-357.

187. Xin Z, Jiang S, Jiang P, Yan X, Fan C, Di S, Wu G, Yang Y, Reiter RJ, Ji G: Melatonin as a treatment for gastrointestinal cancer: a review. *J Pineal Res* 2015, 58(4):375-387.

188. Xu J, Wang LL, Dammer EB, Li CB, Xu G, Chen SD, Wang G: Melatonin for sleep disorders and cognition in dementia: a meta-analysis of randomized controlled trials. *Am J Alzheimers Dis Other Demen* 2015, 30(5):439-447.

189. Yang Y, Sun Y, Yi W, Li Y, Fan C, Xin Z, Jiang S, Di S, Qu Y, Reiter RJ *et al*: A review of melatonin as a suitable antioxidant against myocardial ischemia-reperfusion injury and clinical heart diseases. *J Pineal Res* 2014, 57(4):357-366.

190. Yang WS, Deng Q, Fan WY, Wang WY, Wang X: Light exposure at night, sleep duration, melatonin, and breast cancer: A dose-response analysis of observational studies. *Eur J Cancer Prev* 2014, 23(4):269-276.

191. Yang L, Yao M, Lan Y, Mo W, Sun YL, Wang J, Wang YJ, Cui XJ: Melatonin for Spinal Cord Injury in Animal Models: A Systematic Review and Network Meta-Analysis. *J Neurotrauma* 2016, 33(3):290-300.

192. Yousaf F, Seet E, Venkatraghavan L, Abrishami A, Chung F: Efficacy and safety of melatonin as an anxiolytic and analgesic in the perioperative period : A qualitative systematic review of randomized trials. *Anesthesiology* 2010, 113(4):968-976.

193. Zetner D, Andersen LP, Rosenberg J: Melatonin as Protection Against Radiation Injury: A Systematic Review. *Drug Res (Stuttg)* 2016, 66(6):281-296.

194. Zhang W, Chen XY, Su SW, Jia QZ, Ding T, Zhu ZN, Zhang T: Exogenous melatonin for sleep disorders in neurodegenerative diseases: a meta-analysis of randomized clinical trials. *Neurol Sci* 2016, 37(1):57-65.

195. Zhang JJ, Meng X, Li Y, Zhou Y, Xu DP, Li S, Li HB: Effects of Melatonin on Liver Injuries and Diseases. *International Journal of Molecular Sciences* 2017, 18(4):23.

196. Oxman AD, Guyatt GH: Validation of an index of the quality of review articles. *J Clin Epidemiol* 1991, 44(11):1271-1278.
