# Supplementary material for: Melatonin and health: an umbrella review of health outcomes and biological mechanisms of action
Source: BMC Med. 2018 Feb 5;16:18. doi: 10.1186/s12916-017-1000-8 (PMC5798185; doi:10.1186/s12916-017-1000-8)
Supplement: Supplementary file 4 — List of randomised trials covered in the systematic reviews. (DOCX 58 kb) [file 12916_2017_1000_MOESM4_ESM.docx]

**Additional file 4: Table S4. List of RCTs covered in the included SRs**

| SR by (year) | Andersen (2014) | Basler (2014) | Braam (2009) | Brzezinski (2005) | Buscemi (2005) | Buscemi (2006) | Chen (2015) | De Crescenzo (2017) | Ferracioli-Oda (2013) | Grossman (2011) | Guaiana (2013) | Hansen (2014) | Hansen (2015) | Huang (2014) | Jansen (2006) | Kuriyama (2014) | Liira (2014) | Liu (2012) | Macleod (2004) | Marrin (2013) | Mihara (2015) | Mills (2005) | Rossignol (2011) | Seely (2012) | Seko (2014) | Van Geijlswijk (2010) | Wang (2012) | Wright (2015) | Yang (2014) | Yang (2016) | Zhang (2016) |
| --- | --- | --- | --- | --- | --- | --- | --- | --- | --- | --- | --- | --- | --- | --- | --- | --- | --- | --- | --- | --- | --- | --- | --- | --- | --- | --- | --- | --- | --- | --- | --- |
| RCT by (year) | | | | | | | | | | | | | | | | | | | | | | | | | | | | | | | |
| Aama 2011 |  |  |  |  |  |  | √ |  |  |  |  |  |  |  |  |  |  |  |  |  |  |  |  |  |  |  |  |  |  |  |  |
| Acil 2004 | √ |  |  |  |  |  |  |  |  |  |  |  | √ |  |  |  |  |  |  |  |  |  |  |  |  |  |  |  |  |  |  |
| Aizawa 2002 |  |  |  |  |  |  |  |  |  |  |  |  |  |  |  |  |  |  |  | √ |  |  |  |  |  |  |  |  |  |  |  |
| Akakin 2013 |  |  |  |  |  |  |  |  |  |  |  |  |  |  |  |  |  |  |  |  |  |  |  |  |  |  |  |  | √ |  |  |
| Almeida 2002 |  |  |  |  |  |  |  |  | √ |  |  |  |  |  |  |  |  |  |  |  |  |  |  |  |  |  |  |  |  |  |  |
| Almenrader 2013 | √ |  |  |  |  |  |  |  |  |  |  |  |  |  |  |  |  |  |  |  |  |  |  |  |  |  |  |  |  |  |  |
| Andrade 2001 |  |  |  |  | √ |  |  |  |  |  |  |  |  |  |  |  |  |  |  |  |  |  |  |  |  |  |  |  |  |  |  |
| Aoki 2006 |  |  |  |  |  |  |  |  |  |  |  |  |  |  |  |  |  |  |  | √ |  |  |  |  |  |  |  |  |  |  |  |
| Asayama 2003 |  |  |  |  |  |  |  |  |  |  |  |  |  |  | √ |  |  |  |  |  |  |  |  |  |  |  |  |  |  |  | √ |
| Ates 2007 |  |  |  |  |  |  |  |  |  |  |  |  |  |  |  |  |  |  |  |  |  |  |  |  |  |  |  |  | √ |  |  |
| Atkinson 2005 a |  |  |  |  |  |  |  |  |  |  |  |  |  |  |  |  |  |  |  | √ |  |  |  |  |  |  |  |  |  |  |  |
| Atkinson 2005 b |  |  |  |  |  |  |  |  |  |  |  |  |  |  |  |  |  |  |  | √ |  |  |  |  |  |  |  |  |  |  |  |
| Attenburrow 1996 |  |  |  | √ |  |  |  |  |  |  |  |  |  |  |  |  |  |  |  |  |  |  |  |  |  |  |  |  |  |  |  |
| Baskett 2003 |  |  |  |  | √ |  |  |  |  |  |  |  |  |  |  |  |  |  |  |  |  |  |  |  |  |  |  |  |  |  |  |
| Bauer 2013 |  |  |  |  |  |  |  |  |  |  |  |  |  |  |  |  |  |  |  |  |  |  |  |  |  |  |  |  |  | √ |  |
| Batioglu 2012 |  |  |  |  |  |  |  |  |  |  |  |  |  |  |  |  |  |  |  |  |  |  |  |  | √ |  |  |  |  |  |  |
| Bjorvatn 2007 |  |  |  |  |  |  |  |  |  |  |  |  |  |  |  |  | √ |  |  |  |  |  |  |  |  |  |  |  |  |  |  |
| Borazan 2010 | √ |  |  |  |  |  |  |  |  |  |  |  |  |  |  |  |  |  |  |  |  |  |  |  |  |  |  |  |  |  |  |
| Borlongan 2000 |  |  |  |  |  |  |  |  |  |  |  |  |  |  |  |  |  |  | √ |  |  |  |  |  |  |  |  |  |  |  |  |
| Brackowski 1994 |  |  |  |  |  |  |  |  |  |  |  |  |  |  |  |  |  |  |  |  |  |  |  | √ |  |  |  |  |  |  |  |
| Braam 2008 |  |  | √ |  |  |  |  |  |  |  |  |  |  |  |  |  |  |  |  |  |  |  |  |  |  |  |  |  |  |  |  |
| Braam 2008 |  |  | √ |  |  |  |  |  |  |  |  |  |  |  |  |  |  |  |  |  |  |  |  |  |  |  |  |  |  |  |  |
| Burgess 2001 |  |  |  |  |  |  |  |  |  |  |  |  |  |  |  |  |  |  |  | √ |  |  |  |  |  |  |  |  |  |  |  |
| CAGO2303 |  |  |  |  |  |  |  |  |  |  | √ |  |  |  |  |  |  |  |  |  |  |  |  |  |  |  |  |  |  |  |  |
| Cagnacci 1992 |  |  |  |  |  |  |  |  |  |  |  |  |  |  |  |  |  |  |  | √ |  |  |  |  |  |  |  |  |  |  |  |
| Cagnacci 2005 |  |  |  |  |  |  |  |  |  | √ |  |  |  |  |  |  |  |  |  |  |  |  |  |  |  |  |  |  |  |  |  |
| Capuzzo 2006 | √ |  |  |  |  |  |  |  |  |  |  |  | √ |  |  |  |  |  |  |  |  |  |  |  |  |  |  |  |  |  |  |
| Cardinali 2002 |  |  |  |  |  |  |  |  |  |  |  |  |  |  |  |  |  |  |  |  |  |  |  |  |  |  |  | √ |  |  |  |
| Camfield 1996 |  |  | √ |  |  | √ |  |  |  |  |  |  |  |  |  |  |  |  |  |  |  |  |  |  |  |  |  |  |  |  |  |
| Caumo 2007 | √ |  |  |  |  |  |  |  |  |  |  |  | √ |  |  |  |  |  |  |  |  |  |  |  |  |  |  |  |  |  |  |
| Caumo 2009 | √ |  |  |  |  |  |  |  |  |  |  |  | √ |  |  |  |  |  |  |  |  |  |  |  |  |  |  |  |  |  |  |
| Cavallo 2004 |  |  |  |  |  |  |  |  |  | √ |  |  |  |  |  |  |  |  |  |  |  |  |  |  |  |  |  |  |  |  |  |
| Cavallo 2005 |  |  |  |  |  |  |  |  |  |  |  |  |  |  |  |  | √ |  |  |  |  |  |  |  |  |  |  |  |  |  |  |
| Cayli 2004 |  |  |  |  |  |  |  |  |  |  |  |  |  |  |  |  |  |  |  |  |  |  |  |  |  |  |  |  | √ |  |  |
| Cerea 2003 |  |  |  |  |  |  |  |  |  |  |  |  |  |  |  |  |  |  |  |  |  |  |  | √ |  |  | √ |  |  |  |  |
| Chojnacki 2011 |  |  |  |  |  |  |  |  |  |  |  | √ |  |  |  |  |  |  |  |  |  |  |  |  |  |  |  |  |  |  |  |
| CL3-022 |  |  |  |  |  |  |  |  |  |  | √ |  |  |  |  |  |  |  |  |  |  |  |  |  |  |  |  |  |  |  |  |
| CL3-023 |  |  |  |  |  |  |  |  |  |  | √ |  |  |  |  |  |  |  |  |  |  |  |  |  |  |  |  |  |  |  |  |
| CL3-024 |  |  |  |  |  |  |  |  |  |  | √ |  |  |  |  |  |  |  |  |  |  |  |  |  |  |  |  |  |  |  |  |
| Cramer 1974 |  |  |  | √ |  |  |  |  |  |  |  |  |  |  |  |  |  |  |  |  |  |  |  |  |  |  |  |  |  |  |  |
| Coppola 2004 |  |  | √ |  |  |  |  |  |  |  |  |  |  |  |  |  |  |  |  |  |  |  |  |  |  |  |  |  |  |  |  |
| Corruble 2013 |  |  |  |  |  |  |  |  |  |  | √ |  |  | √ |  |  |  |  |  |  |  |  |  |  |  |  |  |  |  |  |  |
| Czeisler 2005 |  |  |  |  |  |  |  |  |  |  |  |  |  |  |  |  | √ |  |  |  |  |  |  |  |  |  |  |  |  |  |  |
| Czeisler 2009 |  |  |  |  |  |  |  |  |  |  |  |  |  |  |  |  | √ |  |  |  |  |  |  |  |  |  |  |  |  |  |  |
| Dahlitz 1991 |  |  |  | √ | √ |  |  |  | √ |  |  |  |  |  |  |  |  |  |  |  |  |  |  |  |  | √ |  |  |  |  |  |
| Danilenko  2005 |  |  |  |  |  |  |  | √ |  |  |  |  |  |  |  |  |  |  |  |  |  |  |  |  |  |  |  |  |  |  |  |
| Davis 2001 |  |  |  |  |  |  |  |  |  |  |  |  |  |  |  |  |  |  |  |  |  |  |  |  |  |  |  |  |  | √ |  |
| Dawson 1996 |  |  |  |  |  |  |  |  |  |  |  |  |  |  |  |  |  |  |  | √ |  |  |  |  |  |  |  |  |  |  |  |
| Dawson 1998 |  |  |  |  | √ |  |  |  | √ |  |  |  |  |  |  |  |  |  |  |  |  |  |  |  |  |  |  |  |  |  |  |
| Deacon 1994 |  |  |  |  |  |  |  |  |  |  |  |  |  |  |  |  |  |  |  | √ |  |  |  |  |  |  |  |  |  |  |  |
| Deacon 1995 |  |  |  |  |  |  |  |  |  |  |  |  |  |  |  |  |  |  |  | √ |  |  |  |  |  |  |  |  |  |  |  |
| Dodge 2001 |  |  | √ |  |  | √ |  |  |  |  |  |  |  |  |  |  |  |  |  |  |  |  |  |  |  |  |  |  |  |  |  |
| Dolberg  1998 |  |  |  |  |  |  |  | √ |  |  |  |  |  |  |  |  |  |  |  |  |  |  |  |  |  |  |  |  |  |  |  |
| Dollins 1994 |  |  |  | √ |  |  |  |  |  |  |  |  |  |  |  |  |  |  |  | √ |  |  |  |  |  |  |  |  |  |  |  |
| Dowling 2005 |  |  |  |  |  |  |  |  |  |  |  |  |  |  |  |  |  |  |  |  |  |  |  |  |  |  |  |  |  |  | √ |
| Dowling 2008 |  |  |  |  |  |  |  |  |  |  |  |  |  |  |  |  |  |  |  |  |  |  |  |  |  |  |  |  |  |  | √ |
| Erman 2006 |  |  |  |  |  |  |  |  |  |  |  |  |  |  |  | √ |  | √ |  |  |  |  |  |  |  |  |  |  |  |  |  |
| Erman 2011 |  |  |  |  |  |  |  |  |  |  |  |  |  |  |  |  | √ |  |  |  |  |  |  |  |  |  |  |  |  |  |  |
| Erol 2008 |  |  |  |  |  |  |  |  |  |  |  |  |  |  |  |  |  |  |  |  |  |  |  |  |  |  |  |  | √ |  |  |
| Ersahin 2012 |  |  |  |  |  |  |  |  |  |  |  |  |  |  |  |  |  |  |  |  |  |  |  |  |  |  |  |  | √ |  |  |
| Eryilmaz 2011 |  |  |  |  |  |  |  |  |  |  |  |  |  |  |  |  |  |  |  |  |  |  |  |  | √ |  |  |  |  |  |  |
| Ellis 1996 |  |  |  |  | √ |  |  |  | √ |  |  |  |  |  |  |  |  |  |  |  |  |  |  |  |  |  |  |  |  |  |  |
| Evagelidis 2009 | √ |  |  |  |  |  |  |  |  |  |  |  |  |  |  |  |  |  |  |  |  |  |  |  |  |  |  |  |  |  |  |
| Fava 2012 |  |  |  |  |  |  |  | √ |  |  |  |  |  |  |  |  |  |  |  |  |  |  |  |  |  |  |  |  |  |  |  |
| Folkard 1993 |  |  |  |  |  |  |  |  |  |  |  |  |  |  |  |  | √ |  |  |  |  |  |  |  |  |  |  |  |  |  |  |
| Fujimoto 2000 |  |  |  |  |  |  |  |  |  |  |  |  |  |  |  |  |  |  |  |  |  |  |  |  |  |  |  |  | √ |  |  |
| Garstang 2006 |  |  | √ |  |  |  |  |  |  |  |  |  |  |  |  |  |  |  |  |  |  |  | √ |  |  |  |  |  |  |  |  |
| Garfinkel 1995 |  |  |  | √ | √ |  |  |  |  |  |  |  |  |  |  |  |  |  |  |  |  |  |  |  |  |  |  |  |  |  |  |
| Garfinkel 1997 |  |  |  |  | √ |  |  |  |  |  |  |  |  |  |  |  |  |  |  |  |  |  |  |  |  |  |  |  |  |  |  |
| Garfinke 1999 |  |  |  |  |  |  |  |  |  |  |  |  |  |  |  |  |  |  |  |  |  |  |  |  |  |  |  | √ |  |  |  |
| Garzon 2009 |  |  |  |  |  |  |  |  | √ |  |  | √ |  |  |  |  |  |  |  |  |  |  |  |  |  |  |  | √ |  |  |  |
| Genovese 2005 |  |  |  |  |  |  |  |  |  |  |  |  |  |  |  |  |  |  |  |  |  |  |  |  |  |  |  |  | √ |  |  |
| Gehrman 2009 |  |  |  |  |  |  |  |  |  |  |  |  |  |  |  |  |  |  |  |  |  |  |  |  |  |  |  |  |  |  | √ |
| Gilbert 1999 |  |  |  |  |  |  |  |  |  |  |  |  |  |  |  |  |  |  |  | √ |  |  |  |  |  |  |  |  |  |  |  |
| Girschik 2013 |  |  |  |  |  |  |  |  |  |  |  |  |  |  |  |  |  |  |  |  |  |  |  |  |  |  |  |  |  | √ |  |
| Gogenur 2009 | √ |  |  |  |  |  |  |  |  |  |  |  |  |  |  |  |  |  |  |  |  |  |  |  |  |  |  |  |  |  |  |
| Gooneratne 2010 |  |  |  |  |  |  |  |  |  |  |  |  |  |  |  | √ |  |  |  |  |  |  |  |  |  |  |  |  |  |  |  |
| Grossman 2006 |  |  |  |  |  |  |  |  |  | √ |  |  |  |  |  |  |  |  |  |  |  |  |  |  |  |  |  |  |  |  |  |
| Guizar-Sahagun 2009 |  |  |  |  |  |  |  |  |  |  |  |  |  |  |  |  |  |  |  |  |  |  |  |  |  |  |  |  | √ |  |  |
| Gupta 2002 |  |  |  |  |  |  |  |  |  |  |  |  |  |  |  |  |  |  | √ |  |  |  |  |  |  |  |  |  |  |  |  |
| Hale 2010 |  |  |  |  |  |  |  |  |  |  | √ |  |  | √ |  |  |  |  |  |  |  |  |  |  |  |  |  |  |  |  |  |
| Haghjooy 2013 | √ |  |  |  |  |  |  |  |  |  |  |  |  |  |  |  |  |  |  |  |  |  |  |  |  |  |  |  |  |  |  |
| Haimov 1995 |  |  |  | √ | √ |  |  |  | √ |  |  |  |  |  |  |  |  |  |  |  |  |  |  |  |  |  |  |  |  |  |  |
| Hatta 2014 |  |  |  |  |  |  | √ |  |  |  |  |  |  |  |  |  |  |  |  |  |  |  |  |  |  |  |  |  |  |  |  |
| Hughes 1997 |  |  |  | √ |  |  |  |  |  |  |  |  |  |  |  |  |  |  |  | √ |  |  |  |  |  |  |  |  |  |  |  |
| Hughes 1998 |  |  |  | √ |  |  |  |  |  |  |  |  |  |  |  |  |  |  |  |  |  |  |  |  |  |  |  |  |  |  |  |
| Hurtuk 2011 |  |  |  |  |  |  |  |  |  |  |  | √ |  |  |  |  |  |  |  |  |  |  |  |  |  |  |  |  |  |  |  |
| Ismail, 2009 | √ |  |  |  |  |  |  |  |  |  |  |  | √ |  |  |  |  |  |  |  |  |  |  |  |  |  |  |  |  |  |  |
| Isik 2008 | √ |  |  |  |  |  |  |  |  |  |  |  |  |  |  |  |  |  |  |  |  |  |  |  |  |  |  |  |  |  |  |
| Ionescu  2008 | √ |  |  |  |  |  |  |  |  |  |  |  | √ |  |  |  |  |  |  |  |  |  |  |  |  |  |  |  |  |  |  |
| Jan 1994 |  |  |  |  |  | √ |  |  |  |  |  |  |  |  |  |  |  |  |  |  |  |  |  |  |  |  |  |  |  |  |  |
| James 1987 |  |  |  | √ |  |  |  |  |  |  |  |  |  |  |  |  |  |  |  |  |  |  |  |  |  |  |  |  |  |  |  |
| James 1989 |  |  |  | √ |  |  |  |  |  |  |  |  |  |  |  |  |  |  |  |  |  |  |  |  |  |  |  |  |  |  |  |
| James 1990 |  |  |  |  |  |  |  |  | √ |  |  |  |  |  |  |  |  |  |  |  |  |  |  |  |  |  |  |  |  |  |  |
| James 1998 |  |  |  | √ | √ |  |  |  |  |  |  |  |  |  |  |  | √ |  |  |  |  |  |  |  |  |  |  |  |  |  |  |
| Jean-Louis 1998 |  |  |  |  |  | √ |  |  |  |  |  |  |  |  |  |  |  |  |  |  |  |  |  |  |  |  |  |  |  |  |  |
| Jockovich 2000 |  |  |  |  |  |  |  |  |  |  |  |  |  |  |  |  | √ |  |  |  |  |  |  |  |  |  |  |  |  |  |  |
| Jorgensen 1998 |  |  |  |  |  |  |  |  |  |  |  |  |  |  |  |  | √ |  |  |  |  |  |  |  |  |  |  |  |  |  |  |
| Joo 1998 |  |  |  |  |  |  |  |  |  |  |  |  |  |  |  |  |  |  | √ |  |  |  |  |  |  |  |  |  |  |  |  |
| Jonghe 2014 |  |  |  |  |  |  | √ |  |  |  |  |  |  |  |  |  |  |  |  |  |  |  |  |  |  |  |  |  |  |  |  |
| Kain  2009 |  |  |  |  |  |  |  |  |  |  |  |  |  |  |  |  |  |  |  |  | √ |  |  |  |  |  |  |  |  |  |  |
| Kakizaki 2008 |  |  |  |  |  |  |  |  |  |  |  |  |  |  |  |  |  |  |  |  |  |  |  |  |  |  |  |  |  | √ |  |
| Kasper 2010 | √ |  |  |  |  |  |  |  |  |  | √ |  |  | √ |  |  |  |  |  |  |  |  |  |  |  |  |  |  |  |  |  |
| Kayumov 2001 |  |  |  |  |  |  |  |  | √ |  |  |  |  |  |  |  |  |  |  |  |  |  |  |  |  | √ |  |  |  |  |  |
| Kennedy 2008 |  |  |  |  | √ |  |  |  |  |  | √ |  |  | √ |  |  |  |  |  |  |  |  |  |  |  |  |  |  |  |  |  |
| Khalifa 2013 |  |  |  |  |  |  |  |  |  |  |  |  |  |  |  |  |  |  |  |  | √ |  |  |  |  |  |  |  |  |  |  |
| Khezri & Merate 2013 |  |  |  |  |  |  |  |  |  |  |  |  | √ |  |  |  |  |  |  |  |  |  |  |  |  |  |  |  |  |  |  |
| Kilic 1999 | √ |  |  |  |  |  |  |  |  |  |  |  |  |  |  |  |  |  | √ |  |  |  |  |  |  |  |  |  |  |  |  |
| Kloog 2011 |  |  |  |  |  |  |  |  |  |  |  |  |  |  |  |  |  |  |  |  |  |  |  |  |  |  |  |  |  | √ |  |
| Kohsaka 2011 |  |  |  |  |  |  |  |  |  |  |  |  |  |  |  | √ |  |  |  |  |  |  |  |  |  |  |  |  |  |  |  |
| Kripke 2006 |  |  |  |  |  |  |  |  |  |  |  | √ |  |  |  |  |  |  |  |  |  |  |  |  |  |  |  |  |  |  |  |
| Krauchi 1997 |  |  |  |  |  |  |  |  |  |  |  |  |  |  |  |  |  |  |  | √ |  |  |  |  |  |  |  |  |  |  |  |
| Kucukakin  2010 | √ |  |  |  |  |  |  |  |  |  |  |  |  |  |  |  |  |  |  |  |  |  |  |  |  |  |  |  |  |  |  |
| Kunz 2010 | √ |  |  |  |  |  |  |  | √ |  |  |  |  |  |  |  |  |  |  |  |  |  |  |  |  |  |  |  |  |  | √ |
| Lahteenmaki 2013 |  |  |  |  |  |  |  |  |  |  |  |  |  |  |  |  |  |  |  |  |  |  |  |  |  |  |  | √ |  |  |  |
| Laurant 1997 |  |  |  |  |  |  |  |  |  |  |  |  |  |  |  |  |  |  |  |  |  |  |  |  |  | √ |  |  |  |  |  |
| Lee 2004 |  |  |  |  |  |  |  |  |  |  |  |  |  |  |  |  |  |  | √ |  |  |  |  |  |  |  |  |  |  |  |  |
| Lee 2014 |  |  |  |  |  |  |  |  |  |  |  |  |  |  |  |  |  |  |  |  |  |  |  |  |  |  |  |  | √ |  |  |
| Leibenluft  1997 |  |  |  |  |  |  |  | √ |  |  |  |  |  |  |  |  |  |  |  |  |  |  |  |  |  |  |  |  |  |  |  |
| Lemoine 2007 |  |  |  |  |  |  |  |  | √ |  | √ |  |  | √ |  |  |  |  |  |  |  |  |  |  |  |  |  |  |  |  |  |
| Lewy 1998 |  |  |  |  |  |  |  | √ |  |  |  |  |  |  |  |  |  |  |  |  |  |  |  |  |  |  |  |  |  |  |  |
| Lewy 2006 |  |  |  |  |  |  |  | √ |  |  |  |  |  |  |  |  |  |  |  |  |  |  |  |  |  |  |  |  |  |  |  |
| Li 2010 |  |  |  |  |  |  |  |  |  |  |  |  |  |  |  |  |  |  |  |  |  |  |  |  |  |  |  |  |  | √ |  |
| Ling 1999 |  |  |  |  |  |  |  |  |  |  |  |  |  |  |  |  |  |  | √ |  |  |  |  |  |  |  |  |  |  |  |  |
| Lissoni 1990 |  |  |  |  |  |  |  |  |  |  |  |  |  |  |  |  |  |  |  |  |  |  |  | √ |  |  |  |  |  |  |  |
| Lissoni 1992 |  |  |  |  |  |  |  |  |  |  |  |  |  |  |  |  |  |  |  |  |  | √ |  | √ |  |  |  |  |  |  |  |
| Lissoni 1994 a |  |  |  |  |  |  |  |  |  |  |  |  |  |  |  |  |  |  |  |  |  | √ |  | √ |  |  |  |  |  |  |  |
| Lissoni 1994 b |  |  |  |  |  |  |  |  |  |  |  |  |  |  |  |  |  |  |  |  |  | √ |  |  |  |  |  |  |  |  |  |
| Lissoni 1995 |  |  |  |  |  |  |  |  |  |  |  |  |  |  |  |  |  |  |  |  |  | √ |  | √ |  |  |  |  |  |  |  |
| Lissoni 1996 a |  |  |  |  |  |  |  |  |  |  |  |  |  |  |  |  |  |  |  |  |  | √ |  | √ |  |  | √ |  |  |  |  |
| Lissoni 1996 b |  |  |  |  |  |  |  |  |  |  |  |  |  |  |  |  |  |  |  |  |  | √ |  | √ |  |  |  |  |  |  |  |
| Lissoni 1997 |  |  |  |  |  |  |  |  |  |  |  |  |  |  |  |  |  |  |  |  |  | √ |  | √ |  |  | √ |  |  |  |  |
| Lissoni 1999 |  |  |  |  |  |  |  |  |  |  |  |  |  |  |  |  |  |  |  |  |  | √ |  | √ |  |  | **√** |  |  |  |  |
| Lissoni 2000 |  |  |  |  |  |  |  |  |  |  |  |  |  |  |  |  |  |  |  |  |  | √ |  |  |  |  |  |  |  |  |  |
| Lissoni 2002 |  |  |  |  |  |  |  |  |  |  |  |  |  |  |  |  |  |  |  |  |  |  |  | √ |  |  |  |  |  |  |  |
| Lissoni 2003 |  |  |  |  |  |  |  |  |  |  |  |  |  |  |  |  |  |  |  |  |  | √ |  | √ |  |  | √ |  |  |  |  |
| Lissoni 2007 |  |  |  |  |  |  |  |  |  |  |  |  |  |  |  |  |  |  |  |  |  |  |  | √ |  |  | √ |  |  |  |  |
| Lissoni 2008 |  |  |  |  |  |  |  |  |  |  |  |  |  |  |  |  |  |  |  |  |  |  |  | √ |  |  |  |  |  |  |  |
| Liu 2004 |  |  |  |  |  |  |  |  |  |  |  |  |  |  |  |  |  |  |  |  |  |  |  |  |  |  |  |  | √ |  |  |
| Loo 2002a |  |  |  |  |  |  |  |  |  |  | √ |  |  |  |  |  |  |  |  |  |  |  |  |  |  |  |  |  |  |  |  |
| Lusardi 1994 |  |  |  |  |  |  |  |  |  | √ |  |  |  |  |  |  |  |  |  |  |  |  |  |  |  |  |  |  |  |  |  |
| Lusardi 2004 |  |  |  |  |  |  |  |  |  | √ |  |  |  |  |  |  |  |  |  |  |  |  |  |  |  |  |  |  |  |  |  |
| Luthringer 2009 |  |  |  |  |  |  |  |  | √ |  |  |  |  |  |  |  |  |  |  |  |  |  |  |  |  |  |  |  |  |  |  |
| Lu WZ 2005 |  |  |  |  |  |  |  |  |  |  |  | √ |  |  |  |  |  |  |  |  |  |  |  |  |  |  |  |  |  |  |  |
| McArthur 1998 |  |  |  |  |  | √ |  |  |  |  |  |  |  |  |  |  |  |  |  |  |  |  | √ |  |  |  |  |  |  |  |  |
| McElroy 2006 |  |  |  |  |  |  |  |  |  |  |  |  |  |  |  |  |  |  |  |  |  |  |  |  |  |  |  |  |  | √ |  |
| McElroy 2011 |  |  | √ |  |  |  |  |  |  |  |  |  |  |  |  | √ |  |  |  |  |  |  |  |  |  |  |  |  |  |  |  |
| Martinotti 2012 |  |  |  |  |  |  |  |  |  |  | √ |  |  |  |  |  |  |  |  |  |  |  |  |  |  |  |  |  |  |  |  |
| Mayer 2009 |  |  |  |  |  |  |  |  |  |  |  |  |  |  |  | √ |  | √ |  |  |  |  |  |  |  |  |  |  |  |  |  |
| Medeiros 2007 |  |  |  |  |  |  |  |  |  |  |  |  |  |  |  |  |  |  |  |  |  |  |  |  |  |  |  |  |  |  | √ |
| Montes 2003 |  |  |  | √ |  |  |  |  |  |  |  |  |  |  |  |  |  |  |  |  |  |  |  |  |  |  |  |  |  |  |  |
| MowafI & Ismail 2008 |  |  |  |  | √ |  |  |  |  |  |  |  | √ |  |  |  |  |  |  |  |  |  |  |  |  |  |  |  |  |  |  |
| Mundey 2005 | √ |  |  |  |  |  |  |  | √ |  |  |  |  |  |  |  |  |  |  |  |  |  |  |  |  | √ |  |  |  |  |  |
| Naguib & Samarkandi 1999 |  |  |  |  |  |  |  |  |  |  |  |  | √ |  |  |  |  |  |  |  |  |  |  |  |  |  |  |  |  |  |  |
| Naguib & Samarkandi 2000 | √ |  |  |  |  |  |  |  |  |  |  |  | √ |  |  |  |  |  |  |  |  |  |  |  |  |  |  |  |  |  |  |
| Naguib 2006 | √ |  |  |  |  |  |  |  |  |  |  |  | √ |  |  |  |  |  |  |  |  |  |  |  |  |  |  |  |  |  |  |
| Nagtegaal 1998 | √ |  |  |  |  |  |  |  | √ |  |  |  |  |  |  |  |  |  |  |  |  |  |  |  |  | √ |  |  |  |  |  |
| Nazzaro 2011 |  |  |  |  |  |  |  |  |  |  |  |  |  |  |  |  |  |  |  |  |  |  |  |  | √ |  |  |  |  |  |  |
| NCT00671567 2003 |  |  |  |  |  |  |  |  |  |  |  |  |  |  |  | √ |  |  |  |  |  |  |  |  |  |  |  |  |  |  |  |
| NCT00237497 2005 |  |  |  |  |  |  |  |  |  |  |  |  |  |  |  | √ |  |  |  |  |  |  |  |  |  |  |  |  |  |  |  |
| Nickkholgh  2011 | √ |  |  |  |  |  |  |  |  |  |  |  |  |  |  |  |  |  |  |  |  |  |  |  |  |  |  |  |  |  |  |
| O’Callaghan 1999 | √ |  |  |  |  | √ |  |  |  |  |  |  |  |  |  |  |  |  |  |  |  |  |  |  |  |  |  |  |  |  |  |
| O’Leary 2006 |  |  |  |  |  |  |  |  |  |  |  |  |  |  |  |  |  |  |  |  |  |  |  |  |  |  |  |  |  | √ |  |
| Ozcengiz  2011 |  |  | √ |  |  |  |  |  |  |  |  |  |  |  |  |  |  |  |  |  | √ |  |  |  |  |  |  |  |  |  |  |
| Pacchiarotti 2013 |  |  |  |  |  |  |  |  |  |  |  |  |  |  |  |  |  |  |  |  |  |  |  |  | √ |  |  |  |  |  |  |
| Park 2012 |  |  |  |  |  |  |  |  |  |  |  |  |  |  |  |  |  |  |  |  |  |  |  |  |  |  |  |  | √ |  |  |
| Peker 2000 | √ |  |  |  |  |  |  |  |  |  |  |  |  |  |  |  |  |  | √ |  |  |  |  |  |  |  |  |  |  |  |  |
| Pei 2002a |  |  |  |  |  |  |  |  |  |  |  |  |  |  |  |  |  |  | √ |  |  |  |  |  |  |  |  |  |  |  |  |
| Pei 2002b |  |  |  |  |  |  |  |  |  |  |  |  |  |  |  |  |  |  | √ |  |  |  |  |  |  |  |  |  |  |  |  |
| Pei 2003 |  |  |  |  |  |  |  |  |  |  |  |  |  |  |  |  |  |  | √ |  |  |  |  |  |  |  |  |  |  |  |  |
| Peles 2007 |  |  |  |  |  |  |  |  |  |  |  |  |  |  |  |  |  |  |  |  |  |  |  |  |  |  |  | √ |  |  |  |
| Pinheiro 2006 |  |  |  |  |  |  |  |  |  |  |  |  |  |  |  |  |  |  |  |  |  |  |  |  |  |  |  |  |  | √ |  |
| Quera-Salva 2002 |  |  |  |  |  |  |  |  |  |  |  |  |  |  |  |  | √ |  |  |  |  |  |  |  |  |  |  |  |  |  |  |
| Quera-Salva 2011 |  |  |  |  |  |  |  |  |  |  | √ |  |  | √ |  |  |  |  |  |  |  |  |  |  |  |  |  |  |  |  |  |
| Rahman 2010 |  |  |  |  |  |  |  |  |  |  |  | √ |  |  |  |  |  |  |  |  |  |  |  |  |  |  |  |  |  |  |  |
| Rechcinski 2010 |  |  |  |  |  |  |  |  |  | √ |  |  |  |  |  |  |  |  |  |  |  |  |  |  |  |  |  |  |  |  |  |
| Riemersma-van 2008 |  |  |  |  |  |  |  |  |  |  |  | √ |  |  |  |  |  |  |  |  |  |  |  |  |  |  |  |  |  |  |  |
| Rizzo 2010 |  |  |  |  |  |  |  |  |  |  |  |  |  |  |  |  |  |  |  |  |  |  |  |  | √ |  |  |  |  |  |  |
| Reid 1996 |  |  |  |  |  |  |  |  |  |  |  |  |  |  |  |  |  |  |  | √ |  |  |  |  |  |  |  |  |  |  |  |
| Roth 2006 |  |  |  |  |  |  |  |  |  |  |  |  |  |  |  | √ |  | √ |  |  |  |  |  |  |  |  |  |  |  |  |  |
| Roth 2007 |  |  |  |  |  |  |  |  |  |  |  |  |  |  |  | √ |  | √ |  |  |  |  |  |  |  |  |  |  |  |  |  |
| Schernhammer 2005 |  |  |  |  |  |  |  |  |  |  |  |  |  |  |  |  |  |  |  |  |  |  |  |  |  |  |  |  |  | √ |  |
| Schernhammer 2009 |  |  |  |  |  |  |  |  |  |  |  |  |  |  |  |  |  |  |  |  |  |  |  |  |  |  |  |  |  | √ |  |
| Schernhammer 2010 |  |  |  |  |  |  |  |  |  |  |  |  |  |  |  |  |  |  |  |  |  |  |  |  |  |  |  |  |  | √ |  |
| SadeghniiatHghighi 2008 |  |  |  |  |  |  |  |  |  |  |  |  |  |  |  |  | √ |  |  |  |  |  |  |  |  |  |  |  |  |  |  |
| Samarkandi 2005 |  |  |  |  |  |  |  |  |  |  |  |  |  |  |  |  |  |  |  |  | √ |  |  |  |  |  |  |  |  |  |  |
| Sastre-y-Hernandez 1982 |  |  |  |  |  |  |  |  |  |  |  |  |  |  |  |  | √ |  |  |  |  |  |  |  |  |  |  |  |  |  |  |
| Satoh and Mishima 2001 |  |  |  |  |  |  |  |  |  |  |  |  |  |  |  |  |  |  |  | √ |  |  |  |  |  |  |  |  |  |  |  |
| Schernhammer 2008 |  | √ |  |  |  |  |  |  |  |  |  |  |  |  |  |  |  |  |  |  |  |  |  |  |  |  |  |  |  | √ |  |
| Schernhammer 2009 |  | √ |  |  |  |  |  |  |  |  |  |  |  |  |  |  |  |  |  |  |  |  |  |  |  |  |  |  |  |  |  |
| Schernhammer 2010 |  | √ |  |  |  |  |  |  |  |  |  |  |  |  |  |  |  |  |  |  |  |  |  |  |  |  |  |  |  |  |  |
| Scheer 2004 |  | √ |  |  |  |  |  |  |  | √ |  |  |  |  |  |  |  |  |  |  |  |  |  |  |  |  |  |  |  |  |  |
| Schweitzer 2006 |  |  |  |  |  |  |  |  |  |  |  |  |  |  |  |  | √ |  |  |  |  |  |  |  |  |  |  |  |  |  |  |
| Schiaveto 2013 |  |  |  |  |  |  |  |  |  |  |  |  |  |  |  |  |  |  |  |  |  |  |  |  |  |  |  |  | √ |  |  |
| Serfaty 2002 |  |  |  |  |  | √ |  |  |  |  |  |  |  |  | √ |  |  |  |  |  |  |  |  |  |  |  |  |  |  |  | √ |
| Serfaty 2003 |  |  |  |  |  | √ |  |  |  |  |  |  |  |  |  |  |  |  |  |  |  |  |  |  |  |  |  |  |  |  |  |
| Serfaty 2010 |  |  |  |  |  |  |  | √ |  |  |  | √ |  |  |  |  |  |  |  |  |  |  |  |  |  |  |  |  |  |  |  |
| Shamir 2000 |  |  |  |  |  | √ |  |  |  |  |  |  |  |  |  |  |  |  |  |  |  |  |  |  |  |  |  |  |  |  |  |
| Shamir 2000 |  |  |  |  |  | √ |  |  |  |  |  |  |  |  |  |  |  |  |  |  |  |  |  |  |  |  |  |  |  |  |  |
| Sherer 1985 |  |  |  |  |  |  |  | √ |  |  |  |  |  |  |  |  |  |  |  |  |  |  |  |  |  |  |  |  |  |  |  |
| Shu 2013 |  |  |  | √ |  |  |  |  |  |  | √ |  |  |  |  |  |  |  |  |  |  |  |  |  |  |  |  |  |  |  |  |
| Sinha 2001 |  |  |  |  |  |  |  |  |  |  |  |  |  |  |  |  |  |  | √ |  |  |  |  |  |  |  |  |  |  |  |  |
| Singer 2003 |  |  |  |  |  | √ |  |  |  |  |  |  |  |  | √ |  |  |  | √ |  |  |  |  |  |  |  |  |  |  |  | √ |
| Smits 2001 |  |  |  | √ | √ |  |  |  | √ |  |  |  |  |  |  |  |  |  |  |  |  |  |  |  |  | √ |  |  |  |  |  |
| Smits 2003 |  |  |  |  | √ |  |  |  | √ |  |  |  |  |  |  |  |  |  |  |  |  |  |  |  |  | √ |  |  |  |  |  |
| Song |  |  |  |  |  |  |  |  |  |  |  | √ |  |  |  |  |  |  |  |  |  |  |  |  |  |  |  |  |  |  |  |
| Sultan 2010 |  |  |  |  |  |  | √ |  |  |  |  |  |  |  |  |  |  |  |  |  |  |  |  |  |  |  |  |  |  |  |  |
| Sun 2002 |  |  |  |  |  |  |  |  |  |  |  |  |  |  |  |  |  |  | √ |  |  |  |  |  |  |  |  |  |  |  |  |
| Tavukcu 2014 |  |  |  |  |  |  |  |  |  |  |  |  |  |  |  |  |  |  |  |  |  |  |  |  |  |  |  |  | √ |  |  |
| Torii 2004 |  |  |  |  |  |  |  |  |  |  |  |  |  |  |  |  |  |  | √ |  |  |  |  |  |  |  |  |  |  |  |  |
| Travis 2004 |  |  |  |  |  |  |  |  |  |  |  |  |  |  |  |  |  |  |  |  |  |  |  |  |  |  |  |  |  | √ |  |
| Turkistani  2007 |  | √ |  |  |  |  |  |  |  |  |  |  | √ |  |  |  |  |  |  |  |  |  |  |  |  |  |  |  |  |  |  |
| Uchiyama 2011 | √ |  |  |  |  |  |  |  |  |  |  |  |  |  |  | √ |  | √ |  |  |  |  |  |  |  |  |  |  |  |  |  |
| Uchiyama 2011 |  |  |  |  |  |  |  |  |  |  |  |  |  |  |  | √ |  | √ |  |  |  |  |  |  |  |  |  |  |  |  |  |
| Van den Heuve 1999 |  |  |  |  |  |  |  |  |  |  |  |  |  |  |  |  |  |  |  | √ |  |  |  |  |  |  |  |  |  |  |  |
| van Geijlswijk 2010 |  |  |  |  |  |  |  |  | √ |  |  |  |  |  |  |  |  |  |  |  |  |  |  |  |  |  |  |  |  |  |  |
| Van Wieringen 2001 |  |  |  |  |  | √ |  |  |  |  |  |  |  |  |  |  |  |  |  |  |  |  |  |  |  |  |  |  |  |  |  |
| van der Heijden 2007 |  |  |  |  |  |  |  |  |  |  |  |  |  |  |  |  |  |  |  |  |  |  |  |  |  | √ |  |  |  |  |  |
| Verkasalo2005 |  |  |  |  |  |  |  |  |  |  |  |  |  |  |  |  |  |  |  |  |  |  |  |  |  |  |  |  |  | √ |  |
| Vissers 2007 |  |  |  |  |  |  |  |  |  |  |  |  |  |  |  |  |  |  |  |  |  |  |  |  |  |  |  | √ |  |  |  |
| Wade 2007 |  |  |  |  |  |  |  |  | √ |  |  |  |  |  |  |  |  |  |  |  |  |  |  |  |  |  |  |  |  |  |  |
| Wade 2011 |  |  |  |  |  |  |  |  | √ |  |  |  |  |  |  |  |  |  |  |  |  |  |  |  |  |  |  |  |  |  |  |
| Wade 2014 |  |  |  |  |  |  |  |  |  |  |  |  |  |  |  |  |  |  |  |  |  |  |  |  |  |  |  |  |  |  | √ |
| Wang-Weigand 2011 |  |  |  |  |  |  |  |  |  |  |  |  |  |  |  | √ |  |  |  |  |  |  |  |  |  |  |  |  |  |  |  |
| Wasdell 2008 |  |  |  |  |  |  |  |  |  |  |  |  |  |  |  |  |  |  |  |  |  |  | √ |  |  |  |  |  |  |  |  |
| Waldhauser 1990 |  |  | √ |  |  |  |  |  |  |  |  |  |  |  |  |  |  |  |  |  |  |  |  |  |  |  |  |  |  |  |  |
| Weiss 2006 |  |  |  |  |  |  |  |  |  |  |  |  |  |  |  |  |  |  |  |  |  |  |  |  |  | √ |  |  |  |  |  |
| Williams 2002 |  |  |  | √ |  |  |  |  |  |  |  | √ |  |  |  |  |  |  |  |  |  |  |  |  |  |  |  |  |  |  |  |
| Wirojanan 2009 |  |  |  |  |  |  |  |  |  |  |  |  |  |  |  |  |  |  |  |  |  |  | √ |  |  |  |  |  |  |  |  |
| Wright 1998 |  |  |  |  |  |  |  |  |  |  |  |  |  |  |  |  | √ |  |  |  |  |  |  |  |  |  |  |  |  |  |  |
| Wright 2010 |  |  |  |  |  |  |  |  |  |  |  |  |  |  |  |  |  |  |  |  |  |  | √ |  |  |  |  |  |  |  |  |
| Wu 2013 |  |  |  |  |  |  |  |  |  |  |  |  |  |  |  |  |  |  |  |  |  |  |  |  |  |  |  |  |  | √ |  |
| Wurtman 1995 |  |  |  | √ |  |  |  |  |  |  |  |  |  |  |  |  |  |  |  |  |  |  |  |  |  |  |  |  |  |  |  |
| Yan 2001 |  |  |  |  |  |  |  |  |  |  |  |  |  |  |  |  |  |  |  |  |  |  |  |  |  |  | √ |  |  |  |  |
| Yan 2002 |  |  |  |  |  |  |  |  |  |  |  |  |  |  |  |  |  |  |  |  |  |  | √ |  |  |  |  |  |  |  |  |
| Yoon 2002 |  |  |  | √ |  |  |  |  |  |  |  |  |  |  |  |  | √ |  |  |  |  |  |  |  |  |  |  |  |  |  |  |
| Zammit 2007 |  |  |  |  |  |  |  |  |  |  |  |  |  |  |  | √ |  | √ |  |  |  |  |  |  |  |  |  |  |  |  |  |
| Zammit 2009 |  |  |  |  |  |  |  |  |  |  |  |  |  |  |  |  |  | √ |  |  |  |  |  |  |  |  |  |  |  |  |  |
| Zhdanova 1995 |  |  |  | √ |  |  |  |  |  |  |  |  |  |  |  |  |  |  |  |  |  |  |  |  |  |  |  |  |  |  |  |
| Zhdanova 1996 |  |  |  | √ |  |  |  |  |  |  |  |  |  |  |  |  |  |  |  |  |  |  |  |  |  |  |  |  |  |  |  |
| Zhdanova 2001 |  |  |  | √ |  |  |  |  | √ |  |  |  |  |  |  |  |  |  |  |  |  |  |  |  |  |  |  |  |  |  |  |
| Total number of studies (N) | 23 | 5 | 37 | 16 | 13 | 12 | 4 | 8 | 19 | 7 | 13 | 10 | 12 | 6 | 3 | 13 | 15 | 8 | 14 | 16 | 3 | 10 | 6 | 14 | 5 | 9 | 7 | 6 | 13 | 16 | 8 |

**Table 8** Footnote: RCT- randomised controlled trial; SR- systematic review.
