# Supplementary material for: Melatonin and health: an umbrella review of health outcomes and biological mechanisms of action
Source: BMC Med. 2018 Feb 5;16:18. doi: 10.1186/s12916-017-1000-8 (PMC5798185; doi:10.1186/s12916-017-1000-8)
Supplement: Supplementary file 5 — Summary of the adverse effects of MLT reported in the studies included (N = 11). (DOCX 24 kb) [file 12916_2017_1000_MOESM5_ESM.docx]

**Additional file 5: Table S5. Summary of the adverse effects of MLT reported in the included studies (N=11)**

| **Study (year) [Reference]** | **Adverse effect** |
| --- | --- |
| Armour (2004) [1] | Worsening of seizures and asthma |
| Bendz (2010) [2] | Transient headaches and dizziness, abdominal pain |
| Brigo (2016) [3] | Worsening of headache |
| Chaplin (2008) [4] | Headache, pharyngitis, back pain and asthenia |
| Hansen (2014) [5] | Mild daytime sleepiness, headaches, poor sleep, vivid dreams, fuzzy feelings |
| Kuriyama (2014) [6] | Headache, somnolence, fatigue, nasopharyngitis, upper respiratory infection, nausea, dizziness, diarrhea, dyspepsia, dysmenorrhea |
| Leger (2015) [7] | Headache, diarrhoea, dry mouth, alanine aminotransferase increased, somnolence, dizziness and nightmare/abnormal dreams |
| Liu (2012) [8] | Headache, nasopharyngitis and somnolence |
| Mills (2005) [9] | Autoimmune hepatitis (N=1), confusion (N=1), optic neuropathy (N =1), fragmented sleep (N=4), psychotic episode (N=1), nystagmus (N=1), seizures (N=4), headache (N=1) skin eruptions (N=2) |
| No authors listed (2015) [10] | Morning drowsiness, enuresis, headache, dizziness, diarrhoea, rash and hypothermia, slight transient headache and gastro-intestinal symptoms |
| Wang-Weigand (2009) [11] | Headache and somnolence |

**Additional file 5: Table S5.** Footnote: MLT-melatonin; n.m.- not mentioned

**References**

1. Armour D, Paton C: Melatonin in the treatment of insomnia in children and adolescents. *Psychiatr Bull* 2004, 28(6):222-224.

2. Bendz LM, Scates AC: Melatonin treatment for insomnia in pediatric patients with attention-deficit/hyperactivity disorder. *Ann Pharmacother* 2010, 44(1):185-191.

3. Brigo F, Igwe SC: Melatonin as add-on treatment for epilepsy. *Cochrane Database Syst Rev* 2016, 2016(3).

4. Chaplin SMM, Nutt DDMFFF: Melatonin (Circadin): a novel hypnotic for use in older patients. *Prescriber* 2008, 19(20):21-24.

5. Hansen MV, Danielsen AK, Hageman I, Rosenberg J, Gogenur I: The therapeutic or prophylactic effect of exogenous melatonin against depression and depressive symptoms: a systematic review and meta-analysis. *Eur Neuropsychopharmacol* 2014, 24(11):1719-1728.

6. Kuriyama A, Honda M, Hayashino Y: Ramelteon for the treatment of insomnia in adults: a systematic review and meta-analysis. *Sleep Med* 2014, 15(4):385-392.

7. Leger D, Quera-Salva MA, Vecchierini MF, Ogrizek P, Perry CA, Dressman MA: Safety profile of tasimelteon, a melatonin MT1 and MT2 receptor agonist: pooled safety analyses from six clinical studies. *Expert Opin Drug Saf* 2015, 14(11):1673-1685.

8. Liu J, Wang Ln: Ramelteon in the treatment of chronic insomnia: systematic review and meta-analysis. *Int J Clin Pract* 2012, 66(9):867-873.

9. Mills E, Wu P, Seely D, Guyatt G: Melatonin in the treatment of cancer: A systematic review of randomized controlled trials and meta-analysis. *J Pineal Res* 2005, 39(4):360-366.

10. Melatonin for sleep problems in children with neurodevelopmental disorders. *Drug Ther Bull* 2015, 53(10):117-120.

11. Wang-Weigand S, McCue M, Ogrinc F, Mini L: Effects of ramelteon 8 mg on objective sleep latency in adults with chronic insomnia on nights 1 and 2: pooled analysis. *Curr Med Res Opin* 2009, 25(5):1209-1213.
